# Supplementary material for: How does onchocerciasis-related skin and eye disease in Africa depend on cumulative exposure to infection and mass treatment?
Source: PLoS Negl Trop Dis. 2021 Jun 11;15(6):e0009489. doi: 10.1371/journal.pntd.0009489 (PMC8221783; doi:10.1371/journal.pntd.0009489)
Supplement: S1 Text — (PDF) [file pntd.0009489.s001.pdf]

S1 Text: Detailed description of methods, model, and results of sensitivity analyses

This document is a supplement to the following manuscript:

How does onchocerciasis-related skin and eye disease in Africa depend on cumulative exposure to infection and mass treatment?

Authors:

Natalie V.S. Vinkeles Melchers[1]*, Wilma A. Stolk[1], Michele E. Murdoch[2], Belén Pedrique[3], Marielle Kloek[1], Roel Bakker[1], Sake J. de Vlas[1], Luc E. Coffeng[1]*

Author affiliation:

1. Department of Public Health, Erasmus MC, University Medical Center Rotterdam, Rotterdam, The Netherlands.
2. Department of Dermatology, West Herts Hospitals NHS Trust, Watford General Hospital, Watford, Hertfordshire, UK.
3. Drugs for Neglected Diseases initiative (DNDi), Geneva, Switzerland

*l.coffeng@erasmusmc.nl(LEC); *n.vinkelesmelchers@erasmusmc.nl(NVSVM)

Table of Contents

This document 4

1. Model background 4

1.1 General structure of the model 4

1.2 Modelling transmission in ONCHOSIM..5

1.3 Disease framework within ONCHOSIM 5

1.4 Impact of ivermectin on disease 6

2. Additional information regarding data and model quantification 8

2.1 Onchocercal Skin Diseases (OSD) 8

Data 8

Stratification by endemicity 8

Quantification of regression rates for reversible onchocercal skin diseases (OSD) 10

2.2 Onchocercal eye disease (OED)..14

2.3 Calibration of parameters for disease processes 14

2.4 Input specifications 15

2.5 Annotated input file 19

2.5.1 Input file header 20

2.5.2 Simulation 20

2.5.3 Demography..22

2.5.4 Morbidity 23

2.5.5 Exposure...25

2.5.6 Immunity 26

2.5.7 Worm 27

2.5.8 Fly...28

2.5.9 Mass treatment 29

2.5.10 Vector control..31

3. Validation of the model with external data 32

4. Simulations 40

4.1 Detailed methods of simulating scenarios 40

4.2 Model-predicted evolution of age patterns in the prevalence of disease during MDA............41

5. Stochastic variation and sensitivity analysis...44

5.1 Stochastic variation of the main analysis 44
5.2 Re-quantification of the model using alternative biological assumptions 48
5.3 Results of the sensitivity analysis 54
6. References 68

This document

First, we briefly describe some general properties of the ONCHOSIM modelling framework (section 1). In section 2 of this document, we describe in more detail how the model has been quantified with which input specifications, probability distributions, functional relationships, and parameter values used in this study. We then describe the validation of the model with external data in section 3, followed by a more detailed description of the model features for running simulations in section 4. Section 5 shows results of the stochastic variation in the simulations, as well as results of the scenario and sensitivity analysis performed. Section 6 summarises the references used.

1. Model background

ONCHOSIM is an established mathematical model for simulating transmission and control of onchocerciasis in a dynamic population [1,2]. It was developed in the 1990's and thereafter has been used extensively to support decision-making of the Onchocerciasis Control Programme (OCP) in West Africa and later also for the African Programme for Onchocerciasis Control (APOC) [1–7]. ONCHOSIM is a disease-specific configuration of WORMSIM (WORMSIM is a generalised individual-based modelling framework for transmission and control of helminthic infections [8]). A detailed formal description of a previous version of the ONCHOSIM model (v2.58Ap9) including Java source code has been described elsewhere, including instructions for installing and running WORMSIM [4,9]. For the current study, ONCHOSIM version 2.76 was used (more details below) using Java version 8. ONCHOSIM is event-driven, which means that time progresses as a result of events (although monthly events are used for most processes). Model input parameters are specified in a structured XML-file, which can be automatically validated (see section 2.4).

1.1 General structure of the model

ONCHOSIM simulates a dynamic human population and the life histories of individual humans and worms within humans. Transmission of infection in human individuals is simulated through a cloud representing one central population of blackflies. ONCHOSIM combines two simulation techniques; i) stochastic microsimulation is used to calculate the life events of individual persons and their inhabitant parasites; and ii) a deterministic model is used to simulate the dynamics of infective material in the cloud (i.e. the vector population) [4]. Simulated humans are born and die, based on user-specified fertility and demographic life tables. The size and age-composition of the simulated human population depends on the specified fertility and survival rates, as well as an optionally

defined maximum population size. When this maximum population size is exceeded, a fraction (10%) of individuals is removed from the population at random to simulate out-migration.

1.2 Modelling transmission in ONCHOSIM

Pre-control endemicity levels can be reproduced by tuning the transmission parameters to data, as in previous ONCHOSIM models. Simulated humans are exposed to blackflies that transmit infection. Important transmission parameters are: exposure heterogeneity (k), peak age of relative exposure to blackflies (Exa) and relative biting rate (rbr), which are varied in order to calibrate the model for various transmission settings. The exposure heterogeneity is described by a gamma distribution with mean 1.0 and k (shape and rate) equal to 3.5, similar to previous simulation exercises with ONCHOSIM [4,9]. We also used the default assumption of Exa of 20 years as the peak age of relative exposure to blackflies [10–14]. The rbr-values correspond to infection levels, and can thus be tuned to represent the mean mf prevalence of an endemicity level or a continuous scale of infection prevalence. With the k set at 3.5, ONCHOSIM predicts that transmission of infection is unsustainable in areas of mf prevalences below 15% (hypoendemic areas) in the absence of migration of infected flies and/or humans. The prevalence of infection and disease in hypoendemic areas was therefore taken as a 0.10 fraction of that of mesoendemic areas, as in previous exercises [6]. The initial force of infection was fixed, similar to previous work [4].

Only a small percentage of L3 larvae successfully transmitted by the vector actually develop into male and female adult worms in the human host. Successfully transmitted L3 larvae take one year to develop into patent worms [15–17]. Patent female worms produce mf, but only if they are inseminated every three months by a patent male worm present in the same host. The lifespan of adult worms is on average 10 years (allowing for variation between worms, see S2 Table for details), whereas the longevity of mf within the host is nine months (i.e. a fixed duration) [1]. An individual's exposure to fly bites is determined by his/her age, sex, remaining life expectancy, and other undefined random factors (e.g. occupation, behaviour) [4,5].

1.3 Disease framework within ONCHOSIM

Previous versions of ONCHOSIM included a basic disease process that only accommodated chronic, irreversible symptoms (e.g. onchocercal eye disease [OED]) and could simulate only one symptom at a time. Acute symptoms like troublesome itch and other types of onchocercal skin disease (OSD) were modelled by means of add-on analyses using statistical models linking ONCHOSIM output on current

infection levels to the prevalence of symptoms (for GBD 2010 and 2013). Such statistical models may not always capture the impact of treatment on morbidity very well, especially for symptoms that correlate not only with current infection status, but also with history of infection. Therefore, we have developed and quantified a generalised module for morbidity within ONCHOSIM by which we can simultaneously model various clinical manifestations, while taking account of history of control, bioclimate, and excess mortality due to blindness. By incorporating disease dynamics within ONCHOSIM, these non-linearities are now captured explicitly. See the main text for details. See S1 Table for expanded model parameters, their interpretation and the mathematical characteristics as used in the model.

1.4 Impact of ivermectin on disease

Treatment rapidly clears mf from the skin and eye, through transportation of mf to regional lymph nodes where they are removed by the human immune system [18]. The impact of mass drug administration (MDA) is modelled by explicitly simulating individual host participation and the effects of drugs on mf and individual worms. Individual host participation to MDA is assumed to be either random (given age and sex) or a mix of random and systematic participation (some people are more inclined to participate than others). In addition, the fraction permanently excluded from MDA is user-defined. The probability of individual participation is determined by the user-defined overall treatment coverage and weights for variation in participation by age and sex. We assume a somewhat lower treatment uptake by children and women of reproductive age, to account for exclusion of pregnant women from treatment [1,2]. Ivermectin is assumed to clear 100% of the mf from the host, and to permanently reduce the capacity of female worms to produce mf by on average 34.9%. We further assume that treatment temporarily stops mf production altogether; mf production then gradually recovers over a period of 11 months on average [19]. The proportion of adult worms and prepatent worms killed by ivermectin is assumed to be zero.

S1 Table. Parameters used within the expanded disease module.

Parameter	Interpretation	Support	Function or distribution
$D_{ix}(t)$	Amount of tissue activation / damage with regard to condition x or x_k (a continuum of k mutually exclusive conditions caused by the same process, e.g. visual impairment and blindness) in individual i in month t.	[0,∞)	$\Delta D_{ix}(t) = S_{ix} \times d_i(t) - r_x \times D_{ix}(t)$
S_{ix}	Susceptibility of individual i to developing condition x or x_k.	[0,∞)	$\Gamma(\alpha, \beta)$, where $\alpha = \beta$ such that the mean of the distribution is one.†
r_x	Regression rate, which is the proportion of tissue activation / damage that regresses per month with regard to condition x or x_k.	[0,1)	Constant.
$d_i(t)$	Accrued damage in month t, defined as either the number of microfilariae that die or the number of adult female worms that are present in individual i in month t.	[0,∞)	$d_i(t)$ is simulated by ONCHOSIM
T_x	One or multiple thresholds for the amount of tissue activation / damage with regard to condition or a continuum of k conditions. If $T_{x_k} \leq D_{ix} < T_{x_{k+1}}$, then condition x_k is considered to be present in individual i.¥	(0,∞)	Constant
$R_{x,k}$	Reversibility of k-th stage of condition x in case the amount of tissue activation / damage has dropped below threshold $T_{x,k}$ after having initially exceeded it.	0 or 1	Constant

† Gamma distribution with shape α and rate β. For each condition x or x_k a separate gamma distribution is specified.

¥ In case of x_k representing a continuum of k conditions, these conditions are assumed to be mutually exclusive (e.g. visual impairment and blindness). In other words, as soon as $T_{x_k} \leq D_{ix} < T_{x_{k+1}}$, then conditions x_{k-1}, x_{k-2}, …, x_1, are no longer considered to be present in individual *i*.

2. Additional information regarding data and model quantification

2.1 Onchocercal Skin Diseases (OSD)

Data

We used the multi-country dataset by Murdoch et al. (2002) [20] to quantify our model. Demographic information available in the data included country of residence, district of residence, age and sex. We omitted data from two countries (Ghana and Cameroon), where the sampling technique was based on voluntarily participation through presentation at a central point for examination and treatment rather than random sampling (census). The data from the three remaining countries were based on random sampling, and included 24 villages with a total of 4,810 individuals. *O. volvulus* parasites in these locations are considered to be of the forest type (the rarely blinding type) and are mostly transmitted by *Simulium damnosum* and in some localities by *S. neavei*. Because data from individual villages contained a lot of noise in terms of age-patterns in nodule prevalence and morbidity prevalence, villages were grouped in endemicity categories (meso-, hyper-, and very hyperendemic), and then age-specific prevalence rates were aggregated over villages within each endemicity category. Age was categorised in the following groups: 0-2 years; 3-4 years; 5-9 years; 10-19 years; 20-29 years; 30-49 years; 50+ years. The infection metric in this dataset was the presence of palpable nodules, a proxy of active infection.

Stratification by endemicity

As ONCHOSIM up until this point could not predict the prevalence of nodules as a measure of infection, we needed to convert the prevalence of palpable nodules into mf prevalence in order to reproduce pre-control associations of infection and morbidity. We calibrated the model's transmission parameters in order to reproduce pre-control levels of mf prevalence in three different endemicity categories based on pre-control nodule prevalence in adult males (aged ≥20 years): mesoendemic (≥ 20% and <40%), hyperendemic (≥40% and <65%), and very hyperendemic (≥65%) villages. The conversion, conditional on the *k* and *Exa* transmission parameters, resulted in an average mf prevalence in the general population aged ≥5 years in mesoendemic areas of 47.9% (rbr = 0.305), in hyperendemic areas of 65.2% (rbr = 0.338), and in very hyperendemic areas of 83.3% (rbr = 0.508). With the K set at 3.5, ONCHOSIM predicts that transmission of infection is unsustainable in areas with mf prevalence below 15% (hypoendemic areas) in the absence of migration of infected flies and/or humans. We therefore assumed that infection and morbidity levels in the hypoendemic scenario are 10% of those in the mesoendemic scenario, as in previous modelling exercises [6].

For external validation, we looked at the pre-control association between the nodule prevalence in adult males (≥20 years) and the OCP-standardised (mean) mf prevalence in the general population aged ≥5 years within the model, and compared this to an earlier published association based on data from 148 African villages across former OCP and APOC-countries [21]. Our model output for the three different endemicity levels followed the published association accurately (S1 Fig).

S1 Fig: The model-estimated mean pre-control nodule prevalence translated into mf prevalence in a population of five years and older.

The red bullets represent the association between the model-predicted prevalence of nodule prevalence in adult males and the standardised mf prevalence for each of the endemicity categories. The dashed lines are the 95% confidence intervals around the predicted functional relationship, as published by Coffeng *et al.* [21].

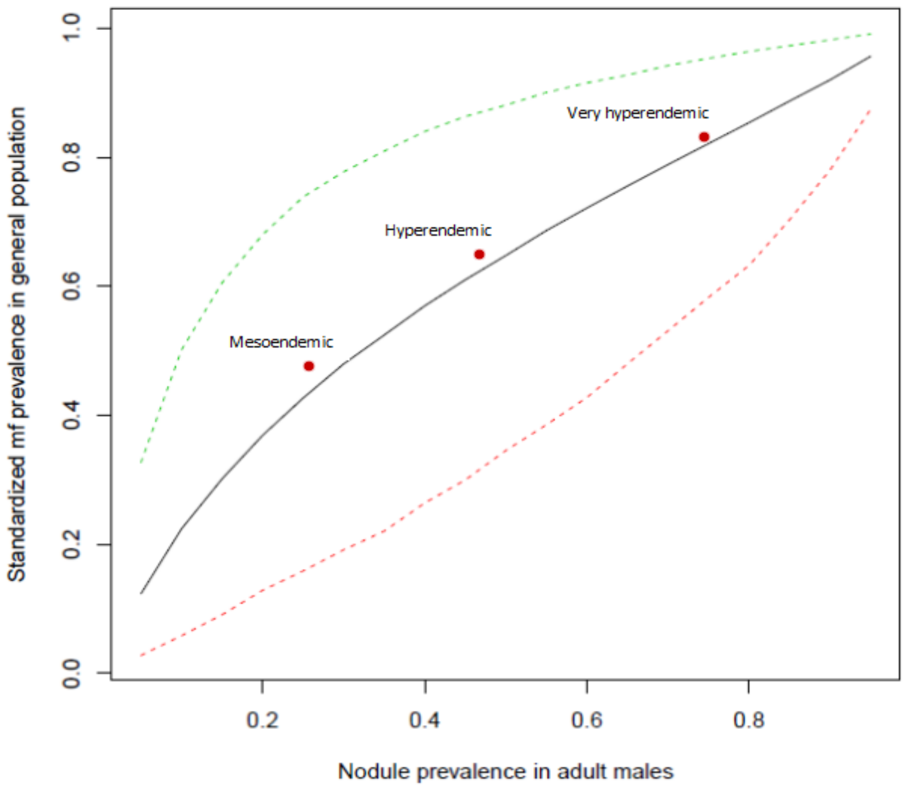

Quantification of regression rates for reversible onchocercal skin diseases (OSD)

The regression rates for acute, reversible clinical manifestations (severe itch, reactive skin disease (RSD), nodules) were quantified by comparing our model output to two cross-sectional data sources on prevalence of morbidity before and up to six years after initiation of MDA [22,23]. One data source showed a reduction in the prevalence of the aforementioned symptoms after one year of annual MDA (with 100% of all followed-up patients having received ivermectin) in predominantly mesoendemic areas [22]; the other data source showed a reduction of symptoms after five to six years (with treatment coverages ranging between 48% and 82%) in predominantly hyperendemic areas, and one mesoendemic area [23].

We performed multiple fit procedures using a grid search based on the sum of squared errors (SSE), where disease parameters for disease threshold and the variation in individual susceptibility to disease were fitted freely conditional on a range of regression rates (see section S2.4 for more details). We then ran simulations with each parameter set for hyperendemic areas where we applied a mean treatment coverage of 55% according to the data [23]. We plotted the various simulated regression lines against the data using the relative reduction in prevalence of morbidity from pre-control to five-six years of MDA for the various reversible clinical manifestations. Similarly, we ran simulations with each parameter set for mesoendemic areas with a treatment coverage of 87.5% (mean of 100% coverage from Brieger *et al.* [22] and 75% coverage from the mesoendemic area reported by Ozoh *et al.* [23]), after which the simulations from the various sets of regression rates were again visualised against the data from both data sources using the relative reduction in prevalence of morbidity from pre-control to one year or five-six years of MDA (S2-S4 Figs).

None of the regression rates reproduced a perfect reduction in prevalence of disease for both time points and endemicity levels simultaneously. The model could capture the relative reduction in prevalence of symptoms better for hyperendemic areas than for mesoendemic areas. Therefore, for each clinical manifestation, a range of regression rates that could best reproduce the longitudinal data were pre-selected for further free fitting of the three disease parameters.

S2 Fig. Model-predicted relative decline in prevalence of severe itch from pre-control to one or five years after initiation of MDA as compared to the data.

The different coloured lines represent the various chosen regression rates (and associated fitted values for damage threshold and variation in susceptibility), the black bullets represent the data with binomial 95% confidence intervals (95%CI) around it. The red dashed line represents the best regression rate for severe itch (regression rate of 0.015) using a sum of squared errors grid search. For hyperendemic areas, the MDA coverage was set at a mean of 55%, according to the data. For mesoendemic areas, the MDA coverage was set at a mean of 74%, based on available data [22,23].

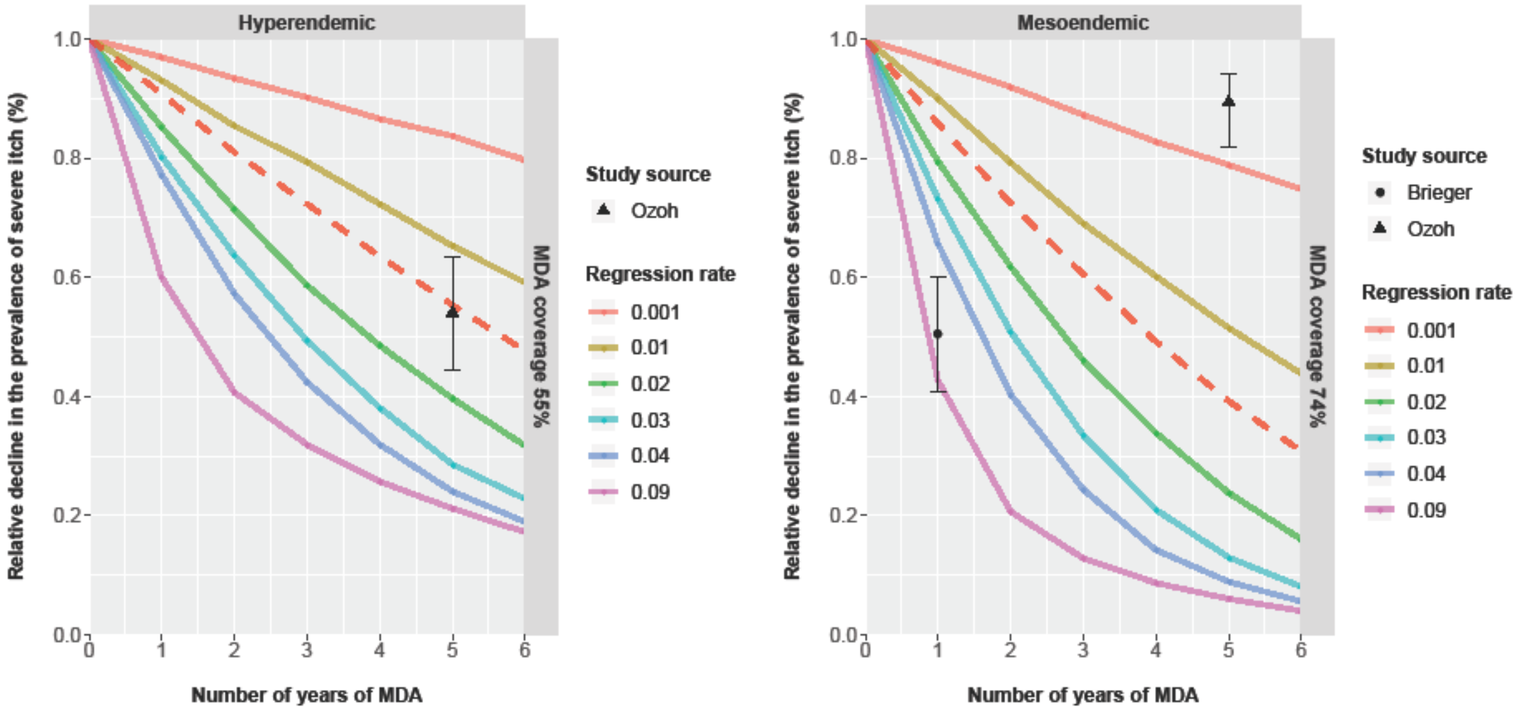

S3 Fig. Model-predicted relative decline in prevalence of RSD from pre-control to one- or five years after initiation of MDA as compared to the data.

The different coloured lines represent the various chosen regression rates (and associated fitted values for damage threshold and variation in susceptibility), the black bullets represent the data with binomial 95% confidence intervals (95%CI) around it. The red dashed line represents the best regression rate for RSD (regression rate of 0.030) using a sum of squared errors grid search. For hyperendemic areas, the MDA coverage was set at a mean of 52.7%. For mesoendemic areas, the MDA coverage was set at a mean of 87.5%, based on available data [22,23]. The regression lines of 0.04 and 0.05 are overlapping between zero and one or two years of MDA, and again after four years of MDA. Between one or two years of MDA and four years of MDA, the predicted relative decline in the prevalence of RSD is steeper with a regression rate of 0.05. This difference is difficult to see in the current format of the figure.

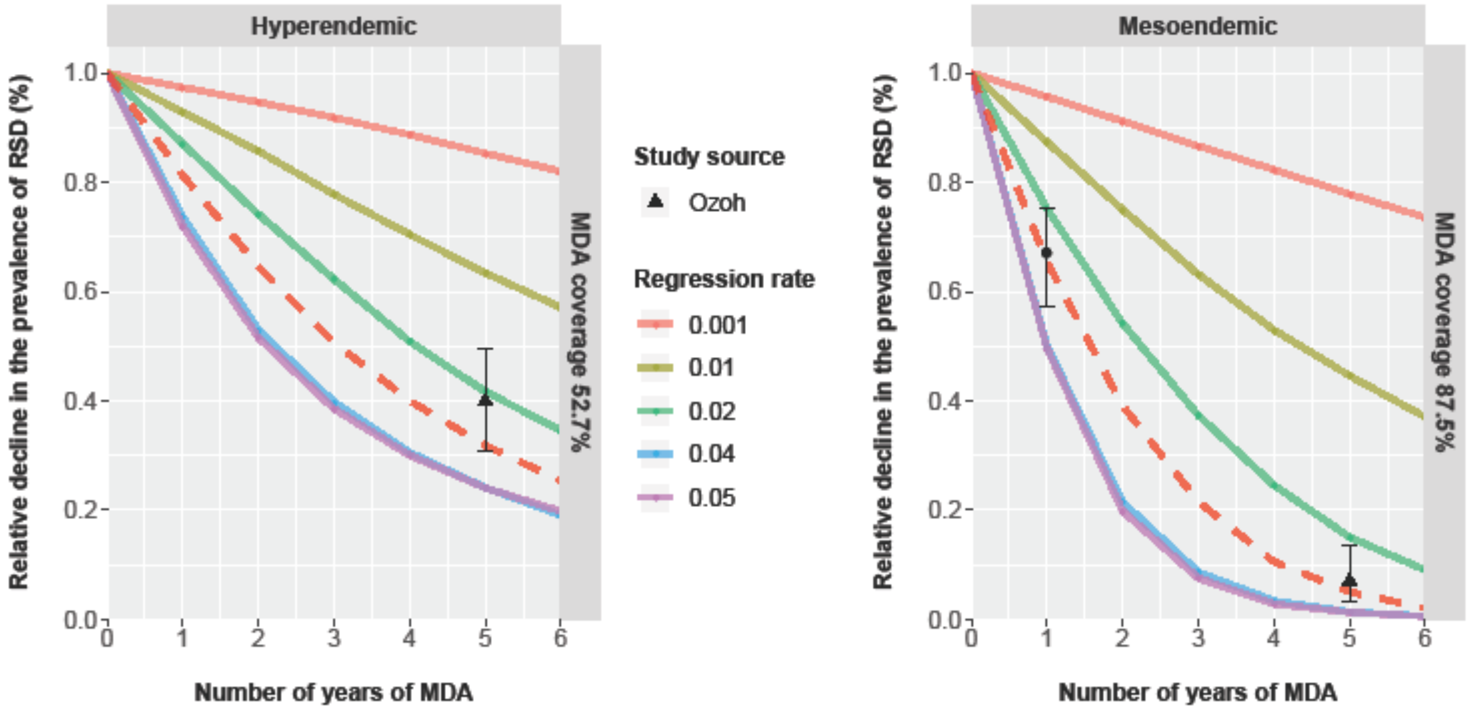

S4 Fig. Model-predicted relative decline in prevalence of palpable nodules from pre-control to five years after initiation of MDA as compared to the data.

The different coloured lines represent the various chosen regression rates (and associated fitted values for damage threshold and variation in susceptibility), the black bullet represents the data with binomial 95% confidence intervals (95%CI) around it. The red dashed line represents the best fitted regression rate for palpable nodules (regression rate of 0.28) using a sum of squared errors grid search. For hyperendemic areas, the MDA coverage was set at a mean of 55% [23]. No data were available for the impact of one year of MDA for the relative reduction in prevalence of palpable nodules, nor for mesoendemic areas. Above a regression rate of 0.20, the level of regression rate becomes irrelevant for the predicted relative decline in the prevalence of palpable nodules with the current MDA coverage (because changes in regression rate are compensated by different refitted values of damage threshold and individual variation in susceptibility). This can be seen as the regression rates for 0.20 to 0.40 are overlying each other.

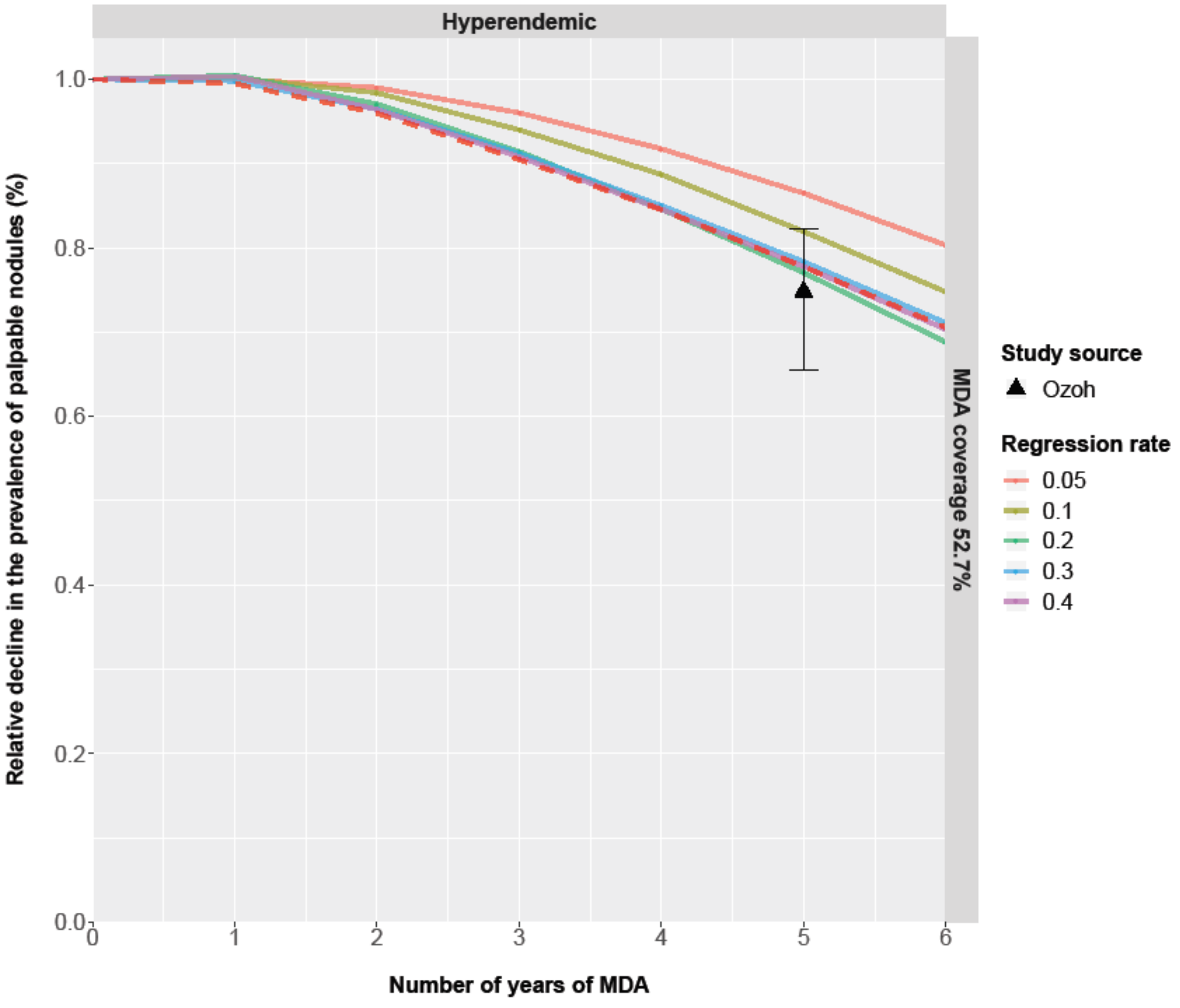

2.2 Onchocercal eye disease (OED)

There is no additional information concerning the data, stratification by endemicity, or model quantification of OED. All methods are described in the main manuscript, under "*Onchocercal eye disease (OED)*" in the Methods section.

2.3 Calibration of parameters for disease processes

We quantified the disease parameters for each clinical manifestation using the SSE grid search. The regression rate for irreversible clinical conditions was set to zero, and we used a range of regression rates for reversible skin clinical manifestation that followed longitudinal trends best [22,23] (see above). For each clinical manifestation, we calibrated the disease parameters, such that when we varied the rbr-values, the age patterns and differences in endemicity levels could be captured adequately. Parameters related to each clinical manifestation were quantified using a grid search using a sum of squared errors (SSE). The grid search measured the deviations predicted from the actual empirical age-specific prevalence of morbidity for each endemicity category by minimising the sum of squared residuals. The least squares finds the line of best model fit for a set of data, and visualises the data and size of the SSE in a three-dimensional grid for each combination of disease parameters. We used the SSE function as follows:

$$\text{SSE} = \text{Sum}(\text{y\_obs\_frac} - \text{y\_hat\_frac})\text{\^{}2 / weight}$$

Where:

y_obs_frac =	Observed prevalence of disease / 100
y_hat_frac =	Model-predicted prevalence of disease / 100
Weight =	Variance of prevalences over villages for an age group (standardisation weights)

The minimum and maximum values of the grid for each fit procedure are pre-defined by setting a range in values for each disease parameter, in which the model will run 100 simulations at a grid resolution of 11, combining unique sets of disease parameters within these bounds. At each step in the fit procedure, we zoomed-in on the minimum and maximum values of the disease parameters for the grid on the basis of the results of the area on the grid with the smallest SSE of the former fit procedure (on average ~15 steps for further zooming-in of the SSE grid). This resulted in a specific set of disease parameters per clinical manifestation with the smallest SSE, representing the best model fit that could reproduce pre-control disease prevalence over age patterns for the three endemicity levels (OSD) or range in infection intensity (OED). This exercise was performed for each

clinical condition separately. The final input specifications and disease parameters for each clinical manifestation are presented in S3 Table.

2.4 Input specifications

S2 Table. WORMSIM quantification used to simulate onchocerciasis transmission.

**Parameter**			**Source**
**Human demography**			
Cumulative survival, by age<br>Male and female survival are considered to be the same			[24]
	Age	Survival rate	
	0	1.000	
	5	0.804	
	10	0.772	
	15	0.760	
	20	0.740	
	30	0.686	
	50	0.509	
	90	0.000	
Fertility rate per woman, by age			[24]
	Age group	Fertility rate	
	0–15	0.000	
	16 – 19	0.109	
	20–29	0.300	
	30-49	0.119	
	50+	0.000	
Population trimming			
	10% if population size exceeds 440.		Assumption
**Transmission of infection**			
General transmission parameters			
Relative biting rate (rbr)	Were set at 0.305 (mesoendemic), 0.338 (hyperendemic), 0.508 (very hyperendemic) for parameter fitting.		
Overall heterogeneity of exposure K of the human hosts to central reservoir of infection	K = 3.5		
Seasonal variation in transmission (mbr)	104%, 91%, 58%, 75%, 75%, 66%, 102%, 133%, 117%, 128%, 146%, and 105% times the average monthly biting rate (January–December)		[25]
Transmission probability (v), i.e. the probability that a L1 larva in the blackfly successfully develops into a L3 larva that is capable of infecting a human host	v = 0.07345; see reference for the derivation of this value, given parameters for fly biology and development of larvae within the fly.		[5]
Success ratio (sr) and Zoophily (z)	Some previous versions of ONCHOSIM required input on success ratio (sr) and zoophily rate (z). In version 2.76 they are hard-coded in the computer code and not modifiable, but the values are the same as reported elsewhere: sr = 0.31% [10,26] and zoophily = 0.04 [2].		
Individual relative exposure to flies			

Parameter		Source
Relative exposure by age and sex (Exa)	Zero at birth, linearly increasing between ages 0–20 from 0 to 1.0 for men and from 0 to 0.7 for women, and then constant from the age of 20 years onwards	[26]
Variation due to personal factors (fixed throughout life) (α_{Exi})	Gamma distribution with mean 1.0 and shape equal to 3.5	[26], Unpublished data from OCP
Host immunity to incoming infections		
Average impact of host immunity (α_{Imm})	Assumed irrelevant for onchocerciasis, hence α_{Imm}= 0; i.e. no effect of immunity on incoming infections	Assumption
Immunological memory (β_{Imm})	Irrelevant given that α_{Imm}= 0	Assumption
**Life history and productivity of the parasite in the human host**		
Average worm lifespan (Tl)	10 years	[15]
Variation in worm lifespan	Weibull distribution with shape 3.8.	Assumption [15]
Prepatent period (pp)	1 year	[15], which refers to [16,17]
Age-dependent microfilaria production capacity (duration since patency) (R(a))	R(a) = 1 for 0 ≤ a < 5<br>R(a) = 1-((a-5)/15) for 5 ≤ a < 20<br>R(a) = 0 for a > 20	[15], which refers to [27,28]
Longevity of microfilariae within host (Tm)	9 months (fixed)	[26]
Mating cycle (rc)	3 months	[26], which refers to [29,30]
Male potential (pot)	100 female worms.	[26]
Density-dependent female worm reproductive capacity		
Worm contribution to host load of infective material (0(.))	7.6 mf/worm	[26]
**Morbidity**		
Disease threshold (Elc)	See Table 3	
Regression rate	See Table 3	
Individual variation to susceptibility to disease (shape parameter)	See Table 3	
Reduction in remaining life expectancy due to blindness	50%	[31,32]
**Infection dynamics in the cloud**		
Uptake of infectious material by vector (uptake curve U(.))	Exponential saturating function:<br>$f(x) = a(1 - e^{-bx})(1 + e^{-cx})$<br>with parameters a = 1.2, b = 0.0213, and c = 0.0861.	[33], which refers to [34,35]
**Mass treatment coverage**		
Coverage (C_w)	User-defined, varied between simulations between 60%-80%, as explained in the text.	
Proportion of individuals who never	User-defined, varied between simulations between 0%-5%, as explained in the text.	

Parameter				Source
participate in mass treatment				
Proportion of treated people in whom treatment is completely ineffective (randomly selected from all treated)	0%			
Weights for relative participation (Cr(k,s)) by age and sex				Based on unpublished OCP data
	age-group	cr(k,males)	cr(k,females)	
	0-4	0	0	
	5-9	0.75	0.5	
	10-14	0.8	0.7	
	15-19	0.8	0.74	
	20-29	0.7	0.65	
	30-49	0.75	0.7	
	50+	0.8	0.75	
**Drug treatment**				
Proportion of microfilariae cleared from host	100%			[19]
Duration of temporary reduction in female reproductive capacity (Tr_0), average	11 months			[19]
Permanent reduction in female worm reproductive capacity (d_0), average	34.9%			[19]
Proportion of adult worms killed (m_0)	0%			[19]
Relative effectiveness (v)	Weibull distribution with mean 1 and shape 2			[19]
**Vector control**				
Timing	Not used.			
Coverage	Not used.			
**Surveys and diagnostics**				
Nr. of skin snips taken in surveys	2			Assumption
Dispersal factor for worm contribution to measured density of infective material (d)	Exponential distribution with mean 1			[15]
Variability in measured host load of infective material	Poisson distribution with mean ss(t)			[15]

S3 Table. Final values of the free disease parameters in the model. Disease thresholds are presented x1000.

Clinical manifestation	Individual variation in susceptibility	Disease threshold (x1000)	Regression rate per month	Root Mean Square Error
**Onchocercal Skin Disease**				
Severe itch	0.316	0.255	0.015	4.8
Reactive skin disease	0.425	0.210	0.030	4.7
Depigmentation*				
Mild depigmentation	0.246	2.35	¥	3.9
Severe depigmentation	0.246	4.30	¥	3.8
Atrophy	0.279	11.3	¥	0.2
Hanging groin	0.857	21.4	¥	0.5
Onchocercal palpable nodules#	1.816	0.012	0.278	8.6
**Onchocercal Eye Disease**				
**Savanna areas***				
Visual impairment	1.0	1.65	¥	8.1
Blindness	1.0	3.05	¥	6.3
**Forest areas***				
Visual impairment	1.0	10.5	¥	1.9
Blindness	1.0	12.5	¥	1.8

Note: * The parameters for individual variation in susceptibility to multi-stage diseases (depigmentation and onchocercal eye disease) were assumed to be the same for both disease stages. We fitted the disease parameters for savanna versus forest areas also in parallel as the biological process of tissue damage is the same. The fitted individual susceptibility variability was 1.0902, which we rounded off to 1.0.

Tissue damage leading to palpable nodules within an individual is triggered by the presence of adult patent female worms. This is in contrast with all other clinical manifestations which are triggered by the death of mf and the release of Wolbachia which induces inflammatory reactions.

¥ These regression rates were all pre-set to zero, as these clinical manifestations were assumed to be irreversible (chronic).

2.5 Annotated input file

The ONCHOSIM inputfile is an XML file that can be edited with any text editor or alternatively, with an XML editor (such as Oxygen XML Editor). The advantage of using the XML format is that any input file can be validated against an XML Schema (a formal specification of the grammar used in the specific XML dialect used for the WORMSIM input file).

A copy of an annotated input file is included below, showing the input assumptions as used in this study to simulate onchocerciasis transmission with WORMSIM version 2.76. The documentation is split into fragments that cover the different elements of the input file (gray-shaded boxes). Together, these fragments constitute a complete input file. The following elements are distinguished:

- Inputfile header
- Simulation
- Demography
- Morbidity
- Exposure
- Immunity
- Worm
- Fly
- Mass.treatment
- Vector.control

Meaning of the formatting of the input files: Text formatted in green as **<!—- this is a comment -->** denotes a comment. Grouping name tags for sets of input parameters are displayed in **blue**, while **red** indicates the specific parameters for which input is to be given. The actual inputs are found in the quotation marks, formatted in **purple**.

2.5.1 Input file header

```
<?xml version="1.0" encoding="UTF-8"?>
<wormsim.inputfile xmlns:xsi="http://www.w3.org/2001/XMLSchema-instance"
                   xsi:noNamespaceSchemaLocation="wormsim.xsd" >
<!-- Input file for ONCHOSIM: -->
<!-- Wormsim v2.76 -->
<!-- Authors: Natalie Vinkeles Melchers et al. -->
```

2.5.2 Simulation

The <simulation> element specifies the timing of surveys (i.e. output moments), the number of skin snips taken at each survey and the age classes for output and the duration of the warming-up period (also called burn-in period), as described elsewhere [4,9]. A simulation always starts with an uninfected human population. To trigger the transmission, one can introduce parasites in the initial population by letting an initial force-of-infection act upon it during a given period preceding the actual simulation (see subsection on exposure for further information on the initial force-of-infection). The actual simulation starts with a long warming-up period (200 years), which is used to make sure that both the human population composition and the parasite population are in a dynamic equilibrium before the start of the surveillance and introduction of interventions. The level of the dynamic equilibrium depends on the assumptions regarding the transmission dynamics and chance effects. The warming-up period does not always result in a stable endemic situation. Especially when biting rates are low, transmission of infection may fade out by chance.

```
<!-- general settings for simulation and simulation output -->
<simulation>
<!-- number of skin snip taken per person -->
<!-- and whether output should be provided at -->
<!-- individual level (true) or not(“false”) -->
<surveillance nr.skin-snips="2" individual-output = "false">
<!-- timing of surveys -->
<!-- month 0 represents January 1st -->
<!-- see note regarding "delay" below -->
<start year="1995" month="0" delay="-2"/>
<stop year="2030" month="1"/>
<interval years="0" months="1"/>
<!-- upper bounds of age categories in output -->
<age.classes>
<age.class age.limit="2"/>
<age.class age.limit="5"/>
<age.class age.limit="10"/>
<age.class age.limit="20"/>
<age.class age.limit="30"/>
<age.class age.limit="50"/>
<age.class age.limit="90"/>
</age.classes>
</surveillance>
<warming.up duration="200"/>
<!-- upper bounds and weights of reference population for -->
<!-- age and sex-standardized output (OCP standard pop) -->
<standard.population>
<age.group age.limit="5" n.males="1401" n.females="1353"/>
<age.group age.limit="10" n.males="1769" n.females="1507"/>
<age.group age.limit="15" n.males="1739" n.females="1465"/>
<age.group age.limit="20" n.males="1085" n.females="921"/>
<age.group age.limit="30" n.males="1409" n.females="1738"/>
<age.group age.limit="50" n.males="2388" n.females="2821"/>
<age.group age.limit="90" n.males="1208" n.females="1237"/>
</standard.population>
</simulation>
```

2.5.3 Demography

The <demography> element defines life tables for the male and female population, the maximum population size (above which random persons will be removed), a fertility table, and the initial population size and age distribution. See comments below.

```
<!-- demographic parameters of simulated population -->
<demography>
<!-- whenever the simulated population size exceeds the -->
<!-- the specified maximum, a random fraction (10%) is removed -->
<!-- see note regarding "delay" below -->
<the.reaper max.population.size="440" reap="0.1" delay="-3"/>
<!-- survival represents cumulative survival probability -->
<!-- and is determined by for unspecified ages by linear -->
<!-- interpolation of values for specified age limits -->
<life.table>
<survival age.limit="5" male.survival="0.804" female.survival="0.804"/>
<survival age.limit="10" male.survival="0.772" female.survival="0.772"/>
<survival age.limit="15" male.survival="0.760" female.survival="0.760"/>
<survival age.limit="20" male.survival="0.740" female.survival="0.740"/>
<survival age.limit="30" male.survival="0.686" female.survival="0.686"/>
<survival age.limit="50" male.survival="0.509" female.survival="0.509"/>
<survival age.limit="90" male.survival="0.000" female.survival="0.000"/>
</life.table>
<!-- rates represent probabilities for women to give birth -->
<!-- to one child in some year, given a woman's age -->
<!-- rates are assumed constant within each age category -->
<!-- and ages limits represent upper bounds of categories -->
<!-- see note regarding "delay" below -->
<fertility.table delay="-4">
<fertility age.limit="5" birth.rate="0.000"/>
<fertility age.limit="10" birth.rate="0.000"/>
<fertility age.limit="15" birth.rate="0.000"/>
<fertility age.limit="20" birth.rate="0.109"/>
<fertility age.limit="30" birth.rate="0.300"/>
<fertility age.limit="50" birth.rate="0.119"/>
<fertility age.limit="90" birth.rate="0.000"/>
</fertility.table>
<!-- population size to start simulation with -->
<initial.population>
<age.group age.limit="5" n.males="4" n.females="4"/>
<age.group age.limit="10" n.males="5" n.females="5"/>
<age.group age.limit="15" n.males="3" n.females="3"/>
<age.group age.limit="20" n.males="3" n.females="3"/>
<age.group age.limit="30" n.males="4" n.females="4"/>
<age.group age.limit="50" n.males="6" n.females="6"/>
<age.group age.limit="90" n.males="4" n.females="4"/>
</initial.population>
</demography>
```

2.5.4 Morbidity

The <morbidity> element defines the parameters for development of morbidity, in this case various subtypes of OSD (depigmentation (multi-stage), skin atrophy, hanging groin, nodules, reactive skin disease, severe itch), and OED (visual impairment, blindness). This morbidity module is a refined version of the original morbidity module in ONCHOSIM in order to include multi-stage diseases and to vary the regression rate and the susceptibility parameter shape. As in the original version [1,2,4,6], morbidity is assumed to result from damage induced by mf that accumulates over time. The refined model allows simulation of different sequential stages of this disease (such as visual impairment which can later progress into blindness) and allows for regression of mf-induced damage. Reaching a certain disease stage can lead to a reduction in remaining life expectancy for blindness only. The parameter values were chosen in such a way that model-predicted patterns of morbidity by age and sex are similar to those predicted with the original model.

```
<!—In the case of multi-stage diseases (depigmentation, onchocercal eye
disease); -->
<!-- when a person's cumulative exposure to mf exceeds the first -->
<!-- and second threshold, respectively, a person is considered -->
<!-- to have mild depigmentation or severe depigmentation, -->
<!-- or visual impairment or blindness . Individual variation in -->
<!-- individual variation in susceptibility is modeled by letting -->
<!-- the progression rate vary between individuals, assuming -->
<!—a Weibull distribution with a user-specified shape parameter -->

<!-- parameters for development of subtypes of OSD -->

<disease.processes>
<disease.process name="VisionLoss" cause="mf" susceptibility.shape.param="1.0">
<regression.rate fun.nr="1" a="0"  b="0" c="-1"/>
<disease.stage name="stage-zero" treshold="0"/>
<disease.stage name="stage-one" treshold="1650"> <!—Savanna areas -->
<symptom name="vi"/>
<disease.stage name=" stage-two “treshold ="3050">
<symptom name=" blind "/>
</disease.stage>
</disease.process>

<disease.process name="DPM" cause="mf" susceptibility.shape.param="0.2461">
<regression.rate fun.nr="1" a="0"  b="0" c="-1"/>
<disease.stage name=" stage-zero " treshold="0"/>
<disease.stage name=" stage-one " treshold="2349.7">
<symptom name="dpmStage1"/>
</disease.stage>
<disease.stage name=" stage-two" treshold="4307.8">
<symptom name="dpmStage2"/>
</disease.stage>
</disease.process>
```

```
<disease.process name="Atrophy" cause="mf" susceptibility.shape.param="0.2786">
<regression.rate fun.nr="1" a="0"  b="0" c="-1"/>
<disease.stage name="stage-zero" treshold="0"/>
<disease.stage name="stage-one" treshold="11254.8">
<symptom name="atrophy"/>
</disease.stage>
</disease.process>
<disease.process name="HG" cause="mf" susceptibility.shape.param="0.8573">
<regression.rate fun.nr="1" a="0"  b="0" c="-1"/>
<disease.stage name="stage-zero" treshold="0"/>
<disease.stage name="stage-one" treshold="21389.6">
<symptom name="hg"/>
</disease.stage>
</disease.process>
<disease.process name="Nodules" cause="patent-F-worms"
susceptibility.shape.param="1.8162">
<regression.rate fun.nr="1" a="0.278"  b="0" c="-1"/>
<disease.stage name="stage-zero" treshold="0"/>
<disease.stage name="stage-one" treshold="11.7">
<symptom name="nodules"/>
</disease.stage>
</disease.process>
<disease.process name="RSD" cause="mf" susceptibility.shape.param="0.42513">
<regression.rate fun.nr="1" a="0.030"  b="0" c="-1"/>
<disease.stage name="stage-zero" treshold="0"/>
<disease.stage name="stage-one" treshold="209.1">
<symptom name="rsd"/>
</disease.stage>
</disease.process>
<disease.process name="sevItch" cause="mf" susceptibility.shape.param="0.3162">
<regression.rate fun.nr="1" a="0.015"  b="0" c="-1"/>
<disease.stage name="stage-zero" treshold="0"/>
<disease.stage name="stage-one" treshold="255.5">
<symptom name="sevItch"/>
</disease.stage>
</disease.process>
</disease.processes>

<!-- Upon turning blind, the life-expectancy of a person,  is reduced by a
variable fraction: on average 50%, and uniformly distributed between 0% and
100%. Visual impairment does not influence the remaining life expectancy. -->
<symptom.defs>
<symptom.def name="vi " irreversible="false">
<pct-life-expectancy-reduction dist.nr="1" min = “0“ max = “100“ mean="0" />
</symptom.def>
<symptom.def name="blind" irreversible="true">
<pct-life-expectancy-reduction dist.nr="1" min = “0“ max = “100“ mean="50" />
</symptom.def>
<symptom.def name="dpmStage1">
<pct-life-expectancy-reduction dist.nr="1" min = “0“ max = “100“ mean="0" />
</symptom.def>
<symptom.def name="dpmStage2">
<pct-life-expectancy-reduction dist.nr="1" min = “0“ max = “100“ mean="0" />
</symptom.def>
```

```
<symptom.def name="atrophy">
<pct-life-expectancy-reduction dist.nr="1" min = “0“ max = “100“ mean="0" />
</symptom.def>
<symptom.def name="hg">
<pct-life-expectancy-reduction dist.nr="1" min = “0“ max = “100“ mean="0" />
</symptom.def>
<symptom.def name="nodules">
<pct-life-expectancy-reduction dist.nr="1" min = “0“ max = “100“ mean="0" />
</symptom.def>
<symptom.def name="rsd">
<pct-life-expectancy-reduction dist.nr="1" min = “0“ max = “100“ mean="0" />
</symptom.def>
<symptom.def name="sevItch">
<pct-life-expectancy-reduction dist.nr="1" min = “0“ max = “100“ mean="0" />
</symptom.def>
```

2.5.5 Exposure

The <exposure> element defines the parameters for the exposure of humans to a vector and thereby the contribution of humans to the vector cloud. This has been described elsewhere [4,9].

```
<!-- parameters for exposure to fly bites
<!-- N.B. in WORMSIM we only describe fly bites on humans, -->
<exposure>
<!-- initial force of infection to introduce infection into the simulated
population at the start -->
<!-- of the warming up period; duration in years -->
<initial.foi duration="7.5" foi="4"/>
<!-- parameters for individual exposure to fly bites,  depending on gender,
age, and personal
<!-- factors -->
<male>
<!-- age-dependent exposure, relative to mean exp of adult males, assuming a
linear increase  <!-- between age 0 and 20, after which exposure is 1.0 -->
<exposure.function fun.nr="1" a="0.05" c="1"/>
<!-- individual variation in exposure related to, e.g. occupation and
attractiveness to flies -->
<!-- assuming a gamma distribution with mean one and variation 1/p1 -->
<!-- (shape and rate p1) truncated by "min" and "max" -->
<exposure.index dist.nr="4" min="0" max="20" p1="4.283"/>
</male>
<female>
<!-- age-dependent exposure, relative to mean exposure of adult males, assuming
<!--a linear increase between age 0 and 20, after which exposure -->
<!-- is 0.70 of the level in males -->
<exposure.function fun.nr="1" a="0.035" c="0.7"/>
<!-- individual variation in exposure related to, e.g. occupation and
attractiveness to -->
<!-- flies, assuming a gamma distribution with mean one and variation 1/p1 -->
<!-- (shape and rate p1), truncated by "min" and "max" -->
<exposure.index dist.nr="4" min="0" max="20" p1="4.283"/>
</female>
</exposure>
```

2.5.6 Immunity

The <immunity> element defines the (optional) development of host immunity. Immunity is not considered in the current model.

```
<!-- parameters related to development of host immunity -->
<!-- these are currently set such that no immunity develops -->
<immunity>
<male alpha="0" beta="1">
<immunity.function fun.nr="0" a="1"/>
<immunity.index dist.nr="0" min="0" max="20"/>
</male>
<female alpha="0" beta="1">
<immunity.function fun.nr="0" a="1"/>
<immunity.index dist.nr="0" min="0" max="20"/>
</female>
</immunity>
```

2.5.7 Worm

The <worm> element defines parameters for worm lifespan, prepatent period, mating between male (M) and female (F) worms, age-dependent production of microfilaria, mf density per worm and skin dispersal.

```
<!-- parameters for worm survival and mf production -->
<!-- mf lifespan in months, see note regarding "delay" below -->
<worm mf-lifespan="9" monthly.event.delay="+1">
<!-- worm lifespan in years, allowing for variation between worms,
assuming a -->
<!-- Weibull distribution with mean 10 and shape 3.76, bounded by "min"
and "max" -->
<lifespan dist.nr="3" min="0" max="50" mean="10" p1="3.76"/>
<!-- pre-patent during which worms do not produce mf and -->
<!-- are not affected by ivermectin -->
<prepatent.period dist.nr="0" mean="1"/>
<!-- number of months a female can produce mf with one insemination, and
-->
<!-- number of females one male worm can inseminate per month -->
<!-- if there are more female worms than the total male potential, every
female has -->
<!-- a probability of being inseminated equal to N_mw/N_fm*male.potential
-->
<mating cycle="3" male.potential="100"/>
<!-- mf production by female worms as function of worm -->
<!-- age minus pre-patent period; mf production at unspecified ages is
<!--determined by linear interpolation -->
<age.dependent.mf-production>
<mf-production age.limit="0" production="1"/>
<mf-production age.limit="5" production="1"/>
<mf-production age.limit="20" production="0"/>
</age.dependent.mf-production>
<!-- expected N_mf per worm in skin snip as product of number of mf
contributed -->
 <!-- per fully fecund worm and random dispersal factor representing the
distance -->
<!-- between a worm and site of skin snip, assuming an exponential
distribution, -->
[illegible]
```

2.5.8 Fly

The <fly> element defines parameters that determine the successful uptake and development of L1 larvae into infective L3 larvae and also determines the fly biting rate.

```
<!-- probability that an mf taken up by a fly bite develops into an L3 and is -->
<!-- transmitted to another human (taking account of the fly's gonotropic cycle, -->
<!-- survival, and duration and probability of an ingested mf developing -->
<!-- into an infective L3 and surviving up to the point of -->

<!-- transmission -->
<fly transmission.probability="0.07345">
<!-- functional relation between uptake of mf and mf density in the skin, assuming --
>
 <!-- exponential saturation to maximum level a with initial slope b and shape c -->
<L1-uptake fun.nr="3" a="1.2" b="0.0213" c="0.0861"/>
<!-- seasonal pattern in monthly biting rates (mbr), as observed in Asubende, Ghana -
->
<!-- in the simulation, actual biting rates for an individual are calculated as -->
<!-- product of monthly biting rate in Asubende, a factor representing -->
<!-- the mean exposure in adult males in the simulated -->
<!-- village relative to Asubende ("relative biting rate"), and all other factors -->
<!-- related to gender, age, and individual variation in exposure -->
<!-- to produce some desired endemicity level in the simulation, adjust the -->
<!-- relative biting rate such that mf prevalence or density (distribution) in -->
<!-- the population (output at the desired time point) equals the desired value -->
<!-- N.B. individual variation in exposure to fly bites also determined mean -->
<!-- and distribution of simulated infection levels -->
<!-- rbr = 0.305 for CMFL 5-->
<!-- rbr = 0.329 for CMFL 10-->
<!-- rbr = 0.457 for CMFL 30-->
<!-- rbr = 0.586 for CMFL 55-->
<!-- rbr = 0.720 for CMFL 80-->
<monthly.biting.rates relative.biting.rate="0.305">
<mbr month="1" rate="2670"/>
<mbr month="2" rate="2350"/>
<mbr month="3" rate="1500"/>
<mbr month="4" rate="1920"/>
<mbr month="5" rate="1940"/>
<mbr month="6" rate="1690"/>
<mbr month="7" rate="2630"/>
<mbr month="8" rate="3410"/>
<mbr month="9" rate="3010"/>
<mbr month="10" rate="3290"/>
<mbr month="11" rate="3750"/>
<mbr month="12" rate="2690"/>
</monthly.biting.rates>
</fly>
```

2.5.9 Mass treatment

The <mass.treatment> element defines parameters for the timing of mass treatment rounds, individual compliance (permanent, temporary and age dependent), and effects of employed drugs on mature worms, mf production by female worms and on mf.

```
<!-- parameters for mass treatment -->
<!-- The statement v58=“true” indicates that the mechanisms employed here is -->
<!-- the same as in the previously published WORMSIM variant 2.58Ap9-->
<mass.treatment v58="true">
<compliance.options>
<!-- random fraction of population permanently not eligible for treatment due -->
<!-- to chronic illness and random fraction of population in which ivermectin -->
<!-- does not work due to diarrhoe (temporary effect) -->
<compliance name="default" fraction.excluded="0" fraction.malabsorption="0"
compliance.model="0">
<!-- weights for age and sex-specific compliance, given some expected overall -->
<!-- coverage in the eligible population; weights are constant within age groups -->
<age.and.sex.specific.compliance age.limit="5" male.compliance="1"
female.compliance="1"/>
<age.and.sex.specific.compliance age.limit="10" male.compliance="1"
female.compliance="1"/>
<age.and.sex.specific.compliance age.limit="15" male.compliance="1"
female.compliance="1"/>
<age.and.sex.specific.compliance age.limit="20" male.compliance="1"
female.compliance="1"/>
<age.and.sex.specific.compliance age.limit="30" male.compliance="1"
female.compliance="0.65"/>
<age.and.sex.specific.compliance age.limit="50" male.compliance="1"
female.compliance="1"/>
<age.and.sex.specific.compliance age.limit="90" male.compliance="1"
female.compliance="1"/>
</compliance>

</compliance.options>
<treatment.rounds>
<!-- Timing of individual mass treatment rounds (one line per mass treatment round),
-->
<!-- specifying year, month (0 represents January 1st), and population -->
<!-- coverage (fraction of total village population including those not eligible for
treatment) -->
<!-- Varying between simulated scenarios, see note regarding "delay" below -->

<treatment.round year="2000" month="0" drug="ivermectin" coverage="0.7" delay="-1"/>
<treatment.round year="2001" month="0" drug="ivermectin" coverage="0.7" delay="-1"/>
<treatment.round year="2002" month="0" drug="ivermectin" coverage="0.7" delay="-1"/>
<treatment.round year="2003" month="0" drug="ivermectin" coverage="0.7" delay="-1"/>
<treatment.round year="2004" month="0" drug="ivermectin" coverage="0.7" delay="-1"/>
<treatment.round year="2005" month="0" drug="ivermectin" coverage="0.7" delay="-1"/>
<treatment.round year="2006" month="0" drug="ivermectin" coverage="0.7" delay="-1"/>
```

```xml
<treatment.round year="2007" month="0" drug="ivermectin" coverage="0.7" delay="-1"/>
<treatment.round year="2008" month="0" drug="ivermectin" coverage="0.7" delay="-1"/>
<treatment.round year="2009" month="0" drug="ivermectin" coverage="0.7" delay="-1"/>
<treatment.round year="2010" month="0" drug="ivermectin" coverage="0.7" delay="-1"/>
<treatment.round year="2011" month="0" drug="ivermectin" coverage="0.7" delay="-1"/>
<treatment.round year="2012" month="0" drug="ivermectin" coverage="0.7" delay="-1"/>
<treatment.round year="2013" month="0" drug="ivermectin" coverage="0.7" delay="-1"/>
<treatment.round year="2014" month="0" drug="ivermectin" coverage="0.7" delay="-1"/>
<treatment.round year="2015" month="0" drug="ivermectin" coverage="0.7" delay="-1"/>
<treatment.round year="2016" month="0" drug="ivermectin" coverage="0.7" delay="-1"/>
<treatment.round year="2017" month="0" drug="ivermectin" coverage="0.7" delay="-1"/>
<treatment.round year="2018" month="0" drug="ivermectin" coverage="0.7" delay="-1"/>
<treatment.round year="2019" month="0" drug="ivermectin" coverage="0.7" delay="-1"/>
<treatment.round year="2020" month="0" drug="ivermectin" coverage="0.7" delay="-1"/>
<treatment.round year="2021" month="0" drug="ivermectin" coverage="0.7" delay="-1"/>
<treatment.round year="2022" month="0" drug="ivermectin" coverage="0.7" delay="-1"/>
<treatment.round year="2023" month="0" drug="ivermectin" coverage="0.7" delay="-1"/>
<treatment.round year="2024" month="0" drug="ivermectin" coverage="0.7" delay="-1"/>
<treatment.round year="2025" month="0" drug="ivermectin" coverage="0.7" delay="-1"/>
<treatment.round year="2026" month="0" drug="ivermectin" coverage="0.7" delay="-1"/>
<treatment.round year="2027" month="0" drug="ivermectin" coverage="0.7" delay="-1"/>
<treatment.round year="2028" month="0" drug="ivermectin" coverage="0.7" delay="-1"/>
<treatment.round year="2029" month="0" drug="ivermectin" coverage="0.7" delay="-1"/>
<treatment.round year="2030" month="0" drug="ivermectin" coverage="0.7" delay="-1"/>
<treatment.round year="2031" month="0" drug="ivermectin" coverage="0.7" delay="-1"/>
<treatment.round year="2032" month="0" drug="ivermectin" coverage="0.7" delay="-1"/>
<treatment.round year="2033" month="0" drug="ivermectin" coverage="0.7" delay="-1"/>
<treatment.round year="2034" month="0" drug="ivermectin" coverage="0.7" delay="-1"/>
<treatment.round year="2035" month="0" drug="ivermectin" coverage="0.7" delay="-1"/>
</treatment.rounds>

<v58.drugs>
<!-- ivermectin efficacy, specified according to mechanisms version 2.58, -->
<!-- as permanent reduction in worm capacity to produce mf (cumulative effects -->
<!-- allowed), pattern of how mf production recovers over time (to a new, reduced
<!-- maximum level) and fraction of mf surviving each treatment -->

<v58.drug name="ivermectin" compliance="default" include.prepatent.worms="true">
<v58.treatment.effects permanent.reduction.mf-production="0.349"
period.of.recovery="0.875"
shape.parameter.recovery.function="1.483"
fraction.killed="0">
<fraction.mf.surviving dist.nr="0" mean="0.0"/>
<!-- variability in treatment effects (relative to mean, expected effect), -->
<!-- assuming a Weibull distribution with mean one and shape "p1" -->
<treatment.effect.variability dist.nr="3" mean="1.0" p1="2"/>
</v58.treatment.effects>
</v58.drug>
</v58.drugs>
</mass.treatment>
```

2.5.10 Vector control

The <vector.control> element defines parameters for setting the effectivity of vector control during periods of vector control, where effectivity is expressed as the reduction (fraction) in black fly density.

```
<!—Vector control is not used in this study, but if required the start and
end year of vector control as well as the efficacy of vector control could be
adapted. -->
<vector.control>
<period start.year="2150" stop.year="2160" effectivity="0.95"/>
<period start.year="2165" stop.year="2170" effectivity="0.95"/>
</vector.control>
</wormsim.inputfile>
<!-- In ONCHOSIM, some events may be scheduled at the same time. The
attribute -->
<!-- "delay" specifies at what time an event takes place, relative to other
events -->
```

3. Validation of the model with external data

After the final selection of disease parameters (S3 Table), we validated the model-predicted prevalence of morbidity against external data:

- Ecological association of our pre-control model predictions against the multi-country data;
- Age-specific pre-control prevalence of clinical manifestations using external data;
- Model-predicted concurrency between clinical manifestations versus observed concurrency (data);
- Longitudinal trends in the prevalence of morbidity with MDA.

Ecological association of our pre-control model predictions against external data

For internal validation of our model, we simulated the ecological association between the prevalence of palpable nodules and skin morbidity at the community-level, and plotted this against pre-control field data [20,22,23,36] (Fig 3, main manuscript). We did not fit the model using the ecological association (as the data were not yet available at the time of fitting), but it provides a clear overview of how our model performs at an ecological level at other geographical sites. Only the model-predictions for atrophy and hanging groin have inferior performance in the specific setting of Kaduna, Nigeria (savanna area) [36].

Age-specific pre-control prevalence of clinical manifestations using external data

For external validation of our pre-control model predictions, which were based on data from forest and mixed forest-savanna bioclimate [20], we also compared our model predictions to data from a locality with a savanna bioclimate (Kaduna, Nigeria) [36]. S5 Fig shows that the fit of the model predictions to the available external data is mediocre. Only the model predictions for depigmentation seem to reasonably agree with the Kaduna data. In contrast, prevalences of hanging groin and atrophy were much higher in Kaduna (up to 45% of the female population with atrophy, and 16% of females with hanging groin) than predicted by our model. Differences between these datasets from two different sets of bioclimates may be due to detection variances in the clinical team, but could potentially also be due to differences in morbidity expression between forest and savanna parasite species. With regards to itch, it is important to note that we quantified our model for the prevalence of severe itch, which is more specific to *O. volvulus* infection, whereas the Kaduna data only included “any itch”, regardless of severity, which explains the overall higher prevalences of itch in Kaduna and the absence of an increase with age in those data.

S5 Fig. Model-predicted age-stratified prevalence of morbidity against observed data per endemicity level. The data contained pre-control information from 36 villages in Kaduna (Nigeria) with a nodule prevalence in adult males between 7.3% and 66.7% [36]. The coloured lines represent the model-predicted age-specific pre-control prevalence of morbidity for meso- and hyperendemic areas, whereas the bullets are the observed data points by age. Model-predicted prevalence of severe itch was plotted against the observed prevalence of any itch due to absence of data on severe itch in the data from Murdoch *et al.* 2017 [36] (upper right panel). Troublesome itch was defined as any form of itching with or without insomnia, severe itching was defined as itching with insomnia. The prevalence of atrophy was plotted up to 54 years of age omitting cases of atrophy for individuals 50+ (as in the data) (lower left panel). Note the varying values on the y-axis.

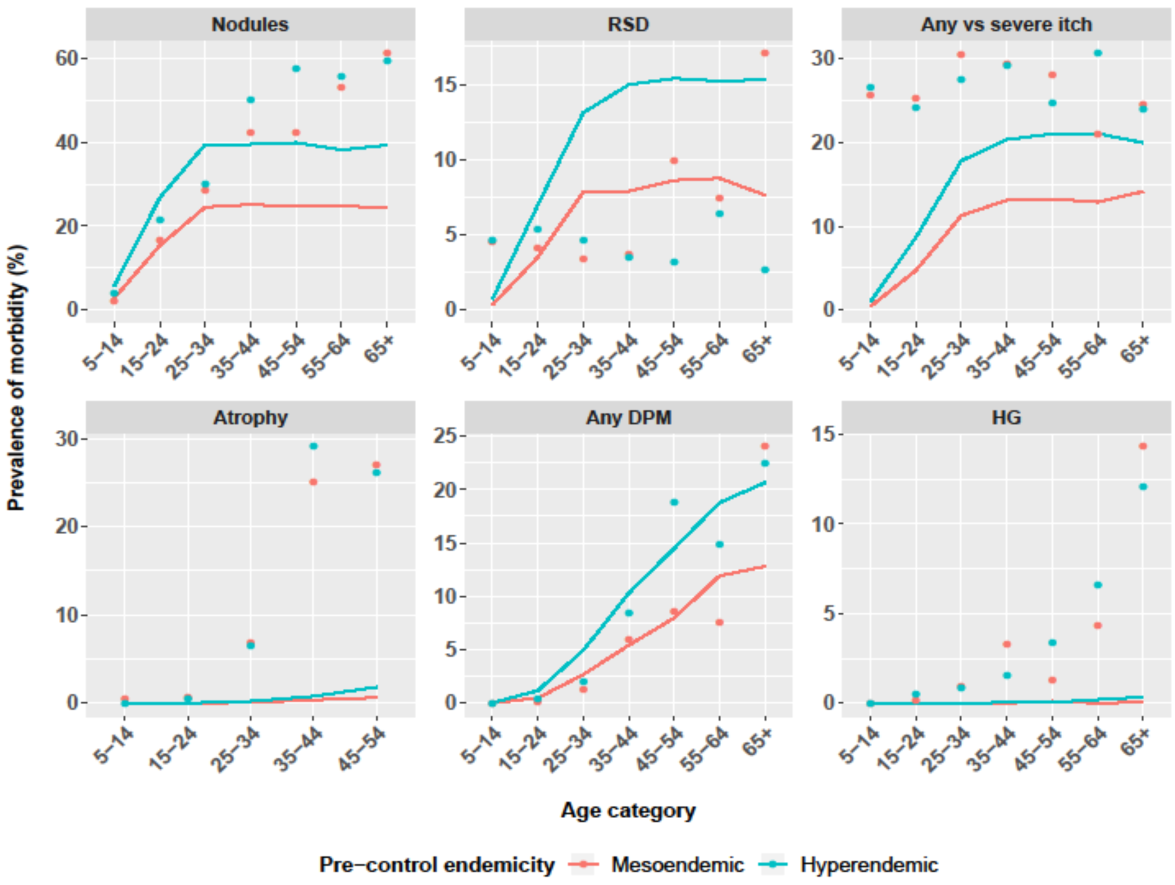

Model-predicted concurrence between clinical manifestations versus observed concurrence

To avoid overestimation of the disease burden of onchocerciasis, burden estimates should account for concurrence of clinical conditions for which the burden is exerted through similar mechanisms [37,38]. For example, many clinical conditions of onchocercal skin disease can be considered to exert a burden (partly) through stigma and low self-esteem due to disfigurement. With this in mind, we validated ONCHOSIM predictions for concurrence of various (groups of) symptom against published data from three population-based epidemiological studies: Murdoch *et al.* 2017 [36], Murdoch *et al.* 2002 [20], and Coffeng *et al.* 2012 [37].

The three population-based studies were summarised in terms of prevalence of a single clinical manifestation (condition without any concurrence of another condition, here referred to as "single condition") and groups of concurring clinical manifestations, meaning the prevalence of at least one symptom within a group of symptoms (here referred to as "concurrent conditions"). Prevalence of single condition or concurrent conditions was calculated after stratifying the data within each study into four endemicity levels based on the village-level prevalence of infection: hypoendemic, mesoendemic, hyperendemic, and very hyperendemic areas, in accordance with our aforementioned endemicity stratifications (section 2.1). The data from Murdoch *et al.* 2002 [20] consisted of 24 villages with in total 4,810 individuals from three countries in Africa (section 2.1), where endemicity varied from hypoendemic to very hyperendemic areas. The data reported by Murdoch *et al.* 2017 [36] consisted of morbidity prevalences as measured in 6,790 individuals across 34 communities from Kaduna State, Nigeria, where endemicity varied from hypoendemic to very hyperendemic areas. Coffeng *et al.* analysed the occurrence between itch, RSD and depigmentation, as well as co-morbidity of depigmentation and visual impairment in mesoendemic areas in Cameroon [37]. We stratified the various subtypes of clinical manifestations into reversible (i.e. severe itch and RSD, palpable nodules) and irreversible (i.e. depigmentation and hanging groin, atrophy, and vision loss) clinical conditions. However, concurrence of vision loss and onchocercal skin conditions was difficult to assess as vision loss was very rare in the one dataset that contained information on it [37] . In addition, the overlap between onchocercal skin diseases and vision loss is regarded to be of low relevance, as both types of conditions involve different mechanisms of imposing a burden on the individual, namely stigmatisation and discomfort due to skin diseases, and vision loss [37]. We also analysed single conditions exclusively without concurrence with other conditions, and groups of concurrent conditions (S8 Fig).

Using ONCHOSIM, we ran 500 simulations and extracted the pre-control prevalence of each single skin condition as well as vision loss, and each combination of concurrent conditions possible with the available data. We assessed the following combinations of concurrent conditions: any itch and RSD; any itch, RSD and any depigmentation; any depigmentation, hanging groin and atrophy; any onchocercal skin disease including palpable nodules; any onchocercal skin disease without palpable nodules. We compared the model-predicted pre-control prevalence of each single condition and the concurrence of clinical manifestations with the pre-control prevalence of the same (groups of) conditions as reported in the data. As the model-predictions for hypoendemic areas are directly deduced from mesoendemic areas (see section 4.1), we have focussed our model validation on meso-, hyper-, and very hyperendemic areas. S7 and S8 Figs show that the performance of the disease module to reproduce observed patterns in concurrency of disease was quite good, especially in meso- and hyperendemic areas. The dashed diagonal line represents 'perfect' conformity between the observed prevalence of (groups of) clinical manifestations according to the data, with the predicted prevalence of the same (groups of) clinical manifestations according to ONCHOSIM simulations. Each bullet thus represents a data point with an observed morbidity prevalence (x-axis), and the corresponding model-predicted prevalence (y-axis).

For very hyperendemic areas, there was some discordance in the model-predicted prevalence of morbidity and the observed prevalence. Some deviation was found in the prevalence of palpable nodules as a single condition with the data from Kaduna (Nigeria) [36] who observed a 11.1% pre-control prevalence in very hyperendemic areas (upper left point in the last panel of S6 and S7 Figs), whereas we predicted a palpable nodule prevalence as a single condition of 55.7% across very hyperendemic areas. On the other hand, our model was quantified using the data from Murdoch *et al.* 2002 [20], who reported a palpable nodule prevalence as a single condition of 56.2%. So here we find a nice match with the data and the model-predictions. We also somewhat underestimated the community-based prevalence of other (groups of) clinical manifestations (i.e. nodules without concurrence, co-morbidity of all skin diseases, RSD without concurrence, co-morbidity of RSD and severe itch) in very hyperendemic areas as compared to the Kaduna (Nigeria) dataset [36] (light blue coloured bullets above the dashed line in the last panel of S7 Fig), whereas the data points from Murdoch *et al.* 2002 [20] are overall nicely located on the dashed line. This is as expected in line with the data that were used for the quantification. Considering the data by Coffeng *et al.* [37] were quite in line with our model-predicted prevalences across mesoendemic areas for vision loss without concurrence, for co-morbidity for irreversible skin conditions (hanging groin, any depigmentation,

and atrophy), and severe itch without concurrence. We overestimated the prevalence of co-morbidity of severe itch and RSD (10.5%), and co-morbidity of severe itch, RSD and any depigmentation (12.5%) as compared to the data by Coffeng *et al.* [37] (0.05% and 2.1%, respectively). Our model-predictions underestimated the prevalence of RSD without concurrence (we predicted 7.6% versus 4.8% by Coffeng *et al.*), and any depigmentation without concurrence (we predicted 3.1% versus 14.9% by Coffeng *et al.*).

We also compared the available prevalence data for the various (groups) of morbidity across hypoendemic areas with our model-predicted prevalence of skin morbidity in those areas. We found that our model-predictions for the prevalence of skin morbidity across hypoendemic areas using a scaling factor of 10% as compared to the prevalence in mesoendemic areas consistently underestimates the observed morbidity prevalences across hypoendemic areas. Most likely, the relative difference in onchocercal morbidity prevalence between hypoendemic versus mesoendemic areas is different from the relative difference in prevalence in infection and may even vary between clinical conditions.

S6 Fig. Model-predicted pre-control prevalence versus observed prevalence of single or concurring clinical manifestations, stratified by data source.

The bullets represent the data points of the comparison between pre-control model-predicted prevalence of single conditions and groups of concurrent conditions and prevalences as reported by three population-based studies (difference in colours of bullets) [20,36,37]. The dashed line represents the 'perfect' association between the pre-control model-predictions and the data. For more information on the comparison between reversible and irreversible clinical manifestations, please see S8 Fig.

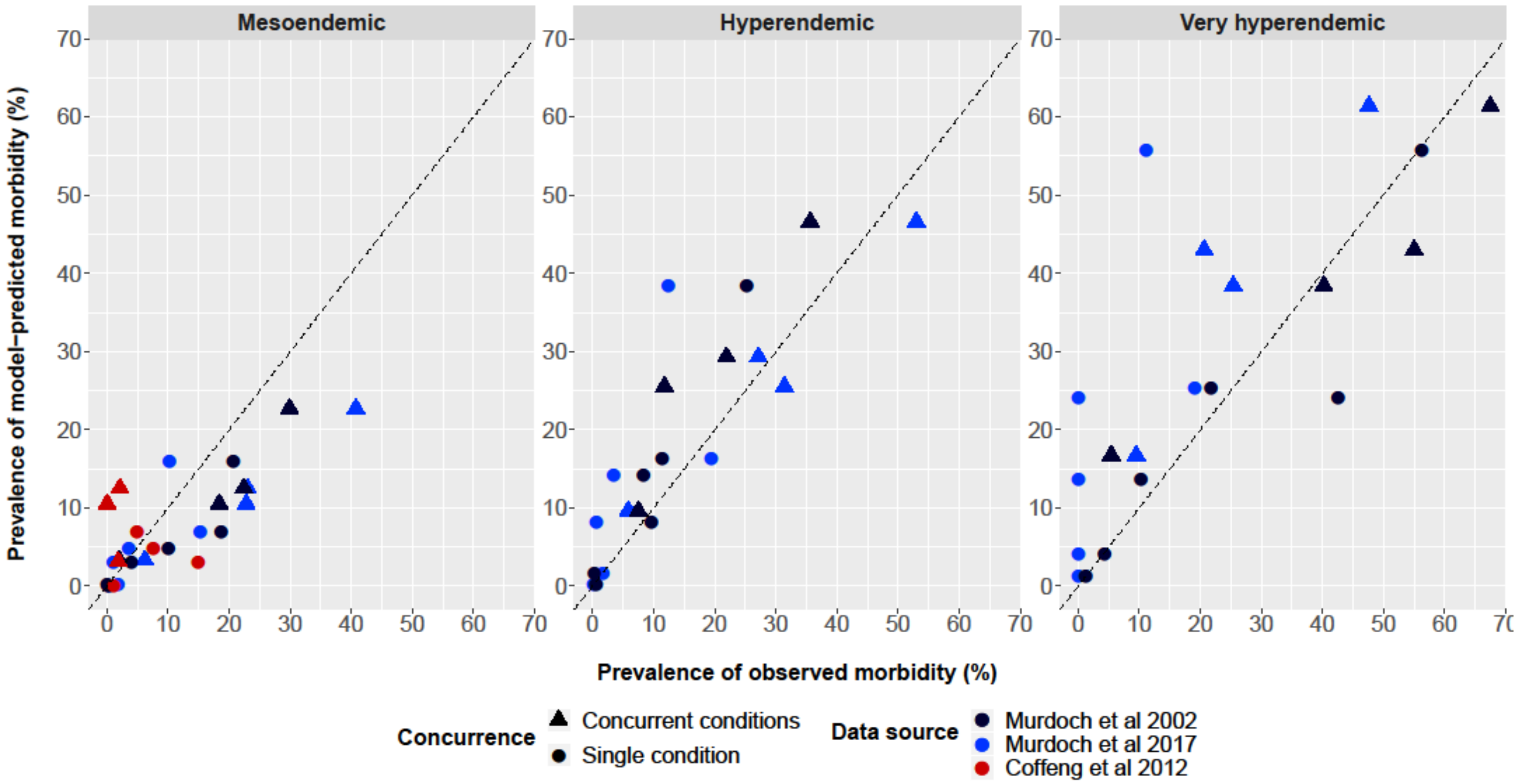

S7 Fig. Model-predicted pre-control prevalence versus observed prevalence of single or concurring clinical manifestations, stratified by reversible and irreversible clinical conditions.

The bullets represent the data points of the comparison between pre-control model-predicted and observed prevalence of single morbidities without concurrence and co-morbidity for reversible versus irreversible morbidity (difference in colours of bullets). The dashed line represents the 'perfect' association between the pre-control model-predictions and the data. Irreversible (chronic) clinical manifestations are defined as any depigmentation, hanging groin, atrophy, and vision loss; reversible (acute) clinical manifestations are defined as severe itch, reactive skin disease and palpable nodules. For more information on the data sources used per bullet, please see S6 Fig.

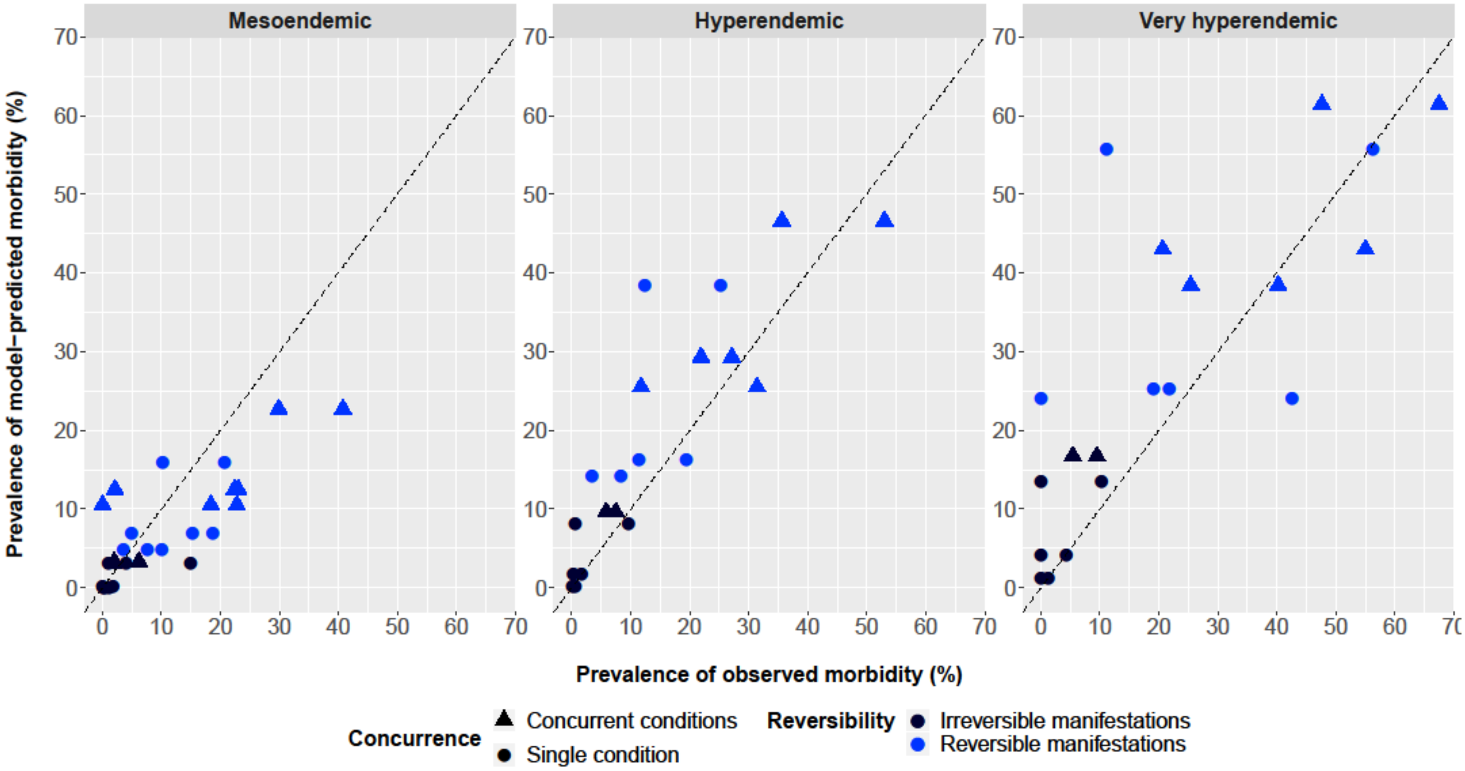

Longitudinal trends in the prevalence of morbidity with MDA

We validated the model-predicted impact of annual MDA over time since the start of mass treatment on the prevalence of morbidity against observed longitudinal data for four clinical manifestations for which data was available (i.e. palpable nodules, RSD, severe itch, and any depigmentation), using two external data sources [22,23]. Our model reproduces the impact of MDA over time quite well, with slight underestimations in pre-control prevalence of RSD and any depigmentation at lower MDA coverages (S8 Fig). When setting the transmission parameters such that it could reproduce the pre-control prevalence of palpable nodules, it consequently also reduced the pre-control prevalence of RSD and depigmentation.

S8 Fig. Longitudinal trends in the model-predicted decline in prevalence of morbidity over time (up to six years) since start of MDA versus observed data.

The blue bullets are the data points from various data sources [22,23] with estimated 95% confidence intervals around the point estimates. The red lines are our model predictions for the respective clinical manifestations (row panels) and treatment coverages (column panels). Please note the varying y-axis per panel.

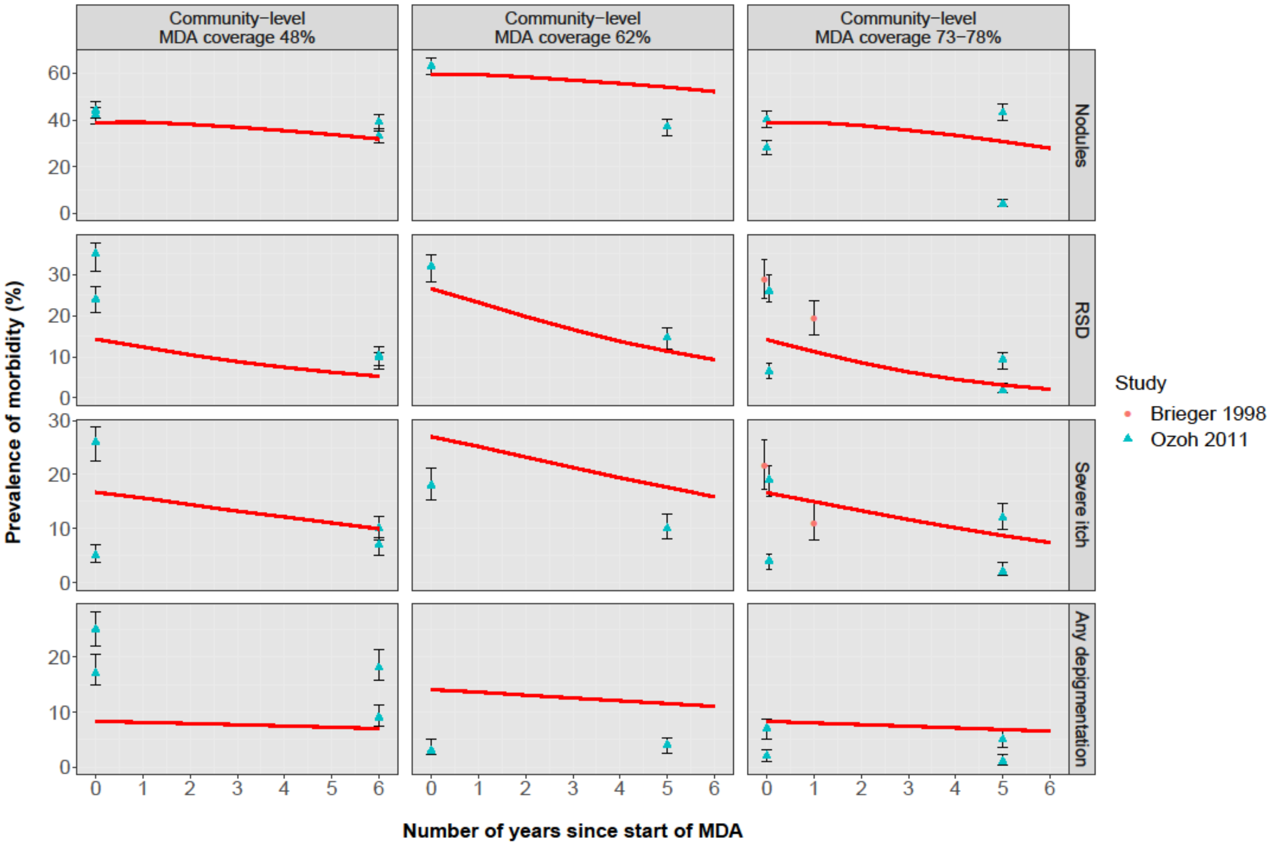

4. Simulations

4.1 Detailed methods of simulating scenarios

After quantification of the model, we simulated trends in infection and morbidity over time for several programmatic scenarios. As ONCHOSIM is a stochastic model, repeated simulations with the same parameter values will lead to slightly different outputs. For clinical manifestations with low baseline prevalences, it is likely that the simulations may result in very little or no morbidity prevalence over time. No stable infection can be simulated for areas with too low mf prevalences (hypoendemic areas) as it is assumed that transmission is not sustained. We therefore performed 750 repeated runs per scenario and took the mean over repeated runs to represent the scenario outcome. Still, in 8% of the simulations in mesoendemic areas, infection did not lead to a stable endemic situation and infection levels withered (especially in clinical manifestations of very low pre-control prevalence: skin atrophy, hanging groin, and OED in forest areas). Therefore, simulations with a pre-control mf prevalence of below 15% were excluded from the scenario means of each endemicity strata. To obtain estimated infection and morbidity prevalences in hypoendemic areas, we multiplied the simulated corresponding prevalence in mesoendemic areas with a ratio of 0.1. For hyper- and very hyperendemic scenarios, endemic equilibrium was achieved in all simulations. We simulated a dynamic population in a typical medium-sized African rural community, assuming a maximum of 440 people. We performed simulations for scenarios pertaining to pre-control endemicity, onchocerciasis type (savanna vs. forest in the case of eye disease) and history of MDA (annual vs. semi-annual, therapeutic coverage of 60%, 70%, and 80%) over a 30 years' timeframe.

For the simulation of scenarios, we applied the mean mf prevalence per endemicity strata such as reported by Prost *et al.* [39]. The mean infection prevalences that are applied to define endemicity levels as used in the simulations are somewhat different than those used during the model calibration and quantification (section 2.2). This is because during model calibration and quantification, we defined endemicity levels on the basis of the available data (equal distribution of villages in each endemicity level). During the simulations, we applied internationally defined endemicity levels, such as those defined by Prost *et al.* [39], as our model results of simulations are easier interpretable.

Prost *et al.* [39] defined hypoendemic areas as areas with a mean mf prevalence (all ages) of <35%; mesoendemic areas with a mean mf prevalence between ≥35% and <60%; hyperendemic areas with a mean mf prevalence between ≥60% and <75%; and very hyperendemic with a mean mf prevalence of ≥75%. In our simulations, we used a rbr-value of 0.316 for mesoendemic areas that corresponds

to a mean mf prevalence among the general population (all ages) of 47.5%; for hyperendemic areas, we used a rbr-value of 0.464 to reproduce a mean mf prevalence (all ages) of 67.4%; for very hyperendemic areas, a rbr-value of 0.772 could reproduce a mean mf prevalence (all ages) of 77.5%. As ONCHOSIM predicts unsustainable transmission of infection in the absence of migration of infected flies and/or humans in hypoendemic areas (mf prevalences below 15%), we assumed again that infection and morbidity levels in the hypoendemic scenario are 10% of those in the mesoendemic scenario, as in previous modelling exercises [8].

4.2 Model-predicted evolution of age patterns in the prevalence of disease during MDA.

S9 Fig shows the age-stratified prevalence of subtypes of OSD, and how the number of years since the start of annual MDA (70% coverage) impacts this prevalence. Without any MDA (zero time since start MDA), the prevalence of reversible conditions (nodules, RSD, severe itch) is predicted to increase rapidly at young ages (from five to 19 years of age onwards, dependent on the pre-control endemicity level). After a minimum of five years of MDA, the age-stratified prevalence of reversible skin diseases is already expected to decline over all age groups. This effect increases with a longer duration of annual MDA, reducing reversible skin diseases after >20 years of MDA to negligible prevalence levels in all age groups.

The prevalence of irreversible conditions (atrophy, depigmentation, hanging groin, and OED) in the absence of MDA is predicted to increase more slowly and linearly with age than reversible conditions, particularly among older age groups (from ~20 years old onwards, depending on the subtype of OSD and bioclimate for OED, as well as pre-control endemicity) (S9 and S10 Figs). Since the implementation of annual MDA, the prevalence of irreversible diseases is predicted to mostly shift to lower prevalence levels with age, but morbidity remains prevalent among older age groups.

S9 Fig. The impact of MDA since the start of treatment with ivermectin on the age-stratified prevalence of subtypes of OSD by endemicity.

The column-panels (and colours) represent the different endemicity levels, and the row panels the various subtypes of OSD. The different types of dashed lines represent the impact of different years of MDA since the start of treatment on the age-stratified prevalence of morbidity. Please note the different scales for the Y-axes in the row panels. Abbreviations: sev. itch = severe itch; RSD = reactive skin disease; DPM = depigmentation; HG = hanging groin.

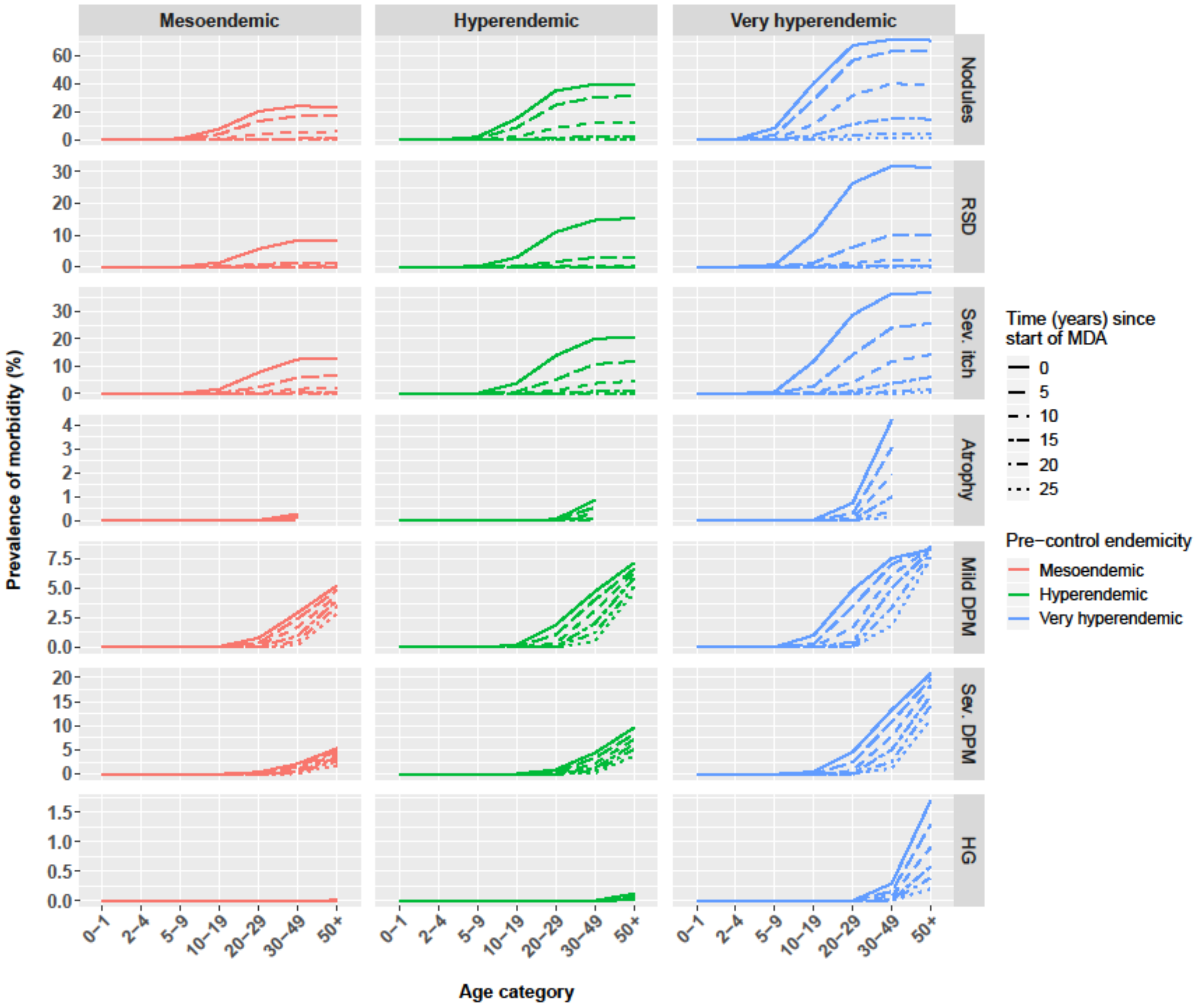

S10 Fig. The impact of MDA since the start of treatment with ivermectin on the age-stratified prevalence of subtypes of onchocercal eye disease (OED) by endemicity.

The column-panels (and colours) represent the different endemicity levels, and the row panels the various subtypes of OED. The different types of dashed lines represent the impact of different years of MDA since the start of treatment on the age-stratified prevalence of morbidity. Please note the different scales for the Y-axes in the row panels. Abbreviations: VI = visual impairment.

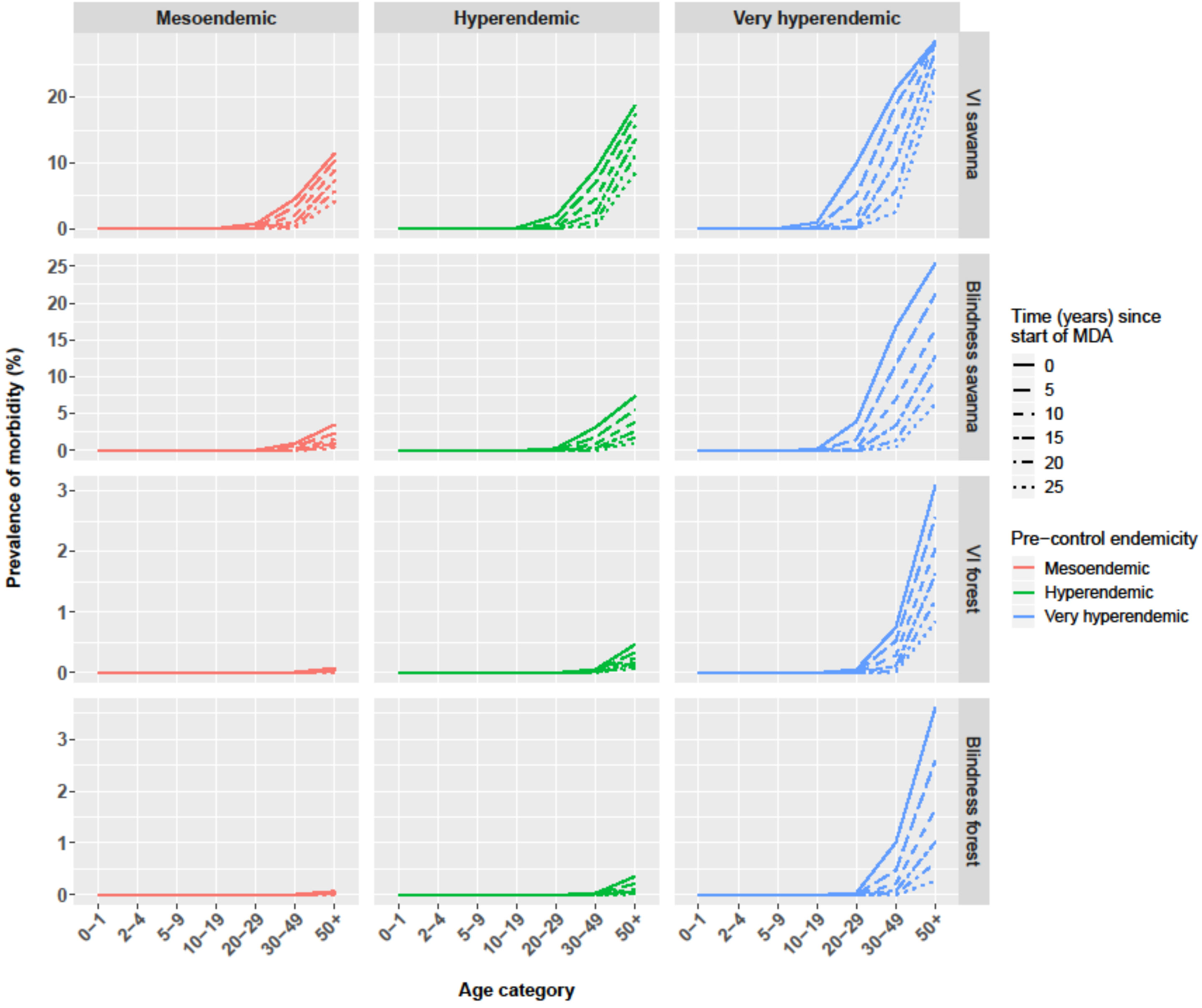

5. Stochastic variation and sensitivity analysis

We assessed how various assumptions inherent to the model influence the prevalence of infection and morbidity over time through univariate sensitivity analyses. We assessed the impact of these different assumptions on various types of infection matrices, namely community-based mf prevalence (all ages) in the general population, community-based mf prevalence for individuals aged five years and older, the adult worm prevalence in the general population, and community microfilarial load (CMFL). CMFL is a measure of intensity of infection in the community; it is defined as the geometric mean number of mf per skin snip among adults aged 20 years and more [40]. We also assessed the impact of these different assumptions on various subtypes of OSD, i.e. mild and severe depigmentation, skin atrophy, hanging groin, palpable nodules, reactive skin disease, and severe itch, as well as several stages of OED, i.e. visual impairment, blindness. Section 5.1 shows the stochastic variation of the trends in the prevalence of infection and clinical manifestations over time using the baseline assumptions (annual MDA with 70% treatment coverage and 50% excess mortality due to blindness). Section 5.2 explains how the alternative assumptions regarding a different regression rate for OED and excess mortality due to blindness change after refitting of disease thresholds only. Section 5.3 shows the results of the simulations after re-quantification of the model with alternative biological assumptions, as well as the impact of various MDA coverages on infection and morbidity prevalence, the treatment frequency (annual versus semi-annual MDA), and the proportion of the population that systematically non-participate in MDA programmes.

5.1 Stochastic variation of the main analysis

The stochastic variation of the main analysis for the various clinical manifestations is presented in S11-S13 Figs. We used the mean predicted trend in prevalence of morbidity over time for the main analysis.

S11 Fig. Stochastic variation of the model output of various types of infection matrices for the scenario of annual MDA with 70% treatment coverage.

Stochastic variation in the prevalence of infection due to *O. volvulus* by endemicity and time since start of MDA in years, with annual treatment coverage of 70%. The predicted trends are based on 750 simulations. For the first three types of infection matrices, the level of infection is the community-based prevalence in the population. For the last type of infection metric, the level of infection is the geometric mean number of mf per person (Community Microfilarial Load [CMFL]). <u>Abbreviations</u>: Mf prev. = Microfilariae prevalence, Mf prev. 5+ = Mf prevalence in age groups ≥5 years, Worm prev. = Adult worm prevalence, CMFL = Community microfilarial load.

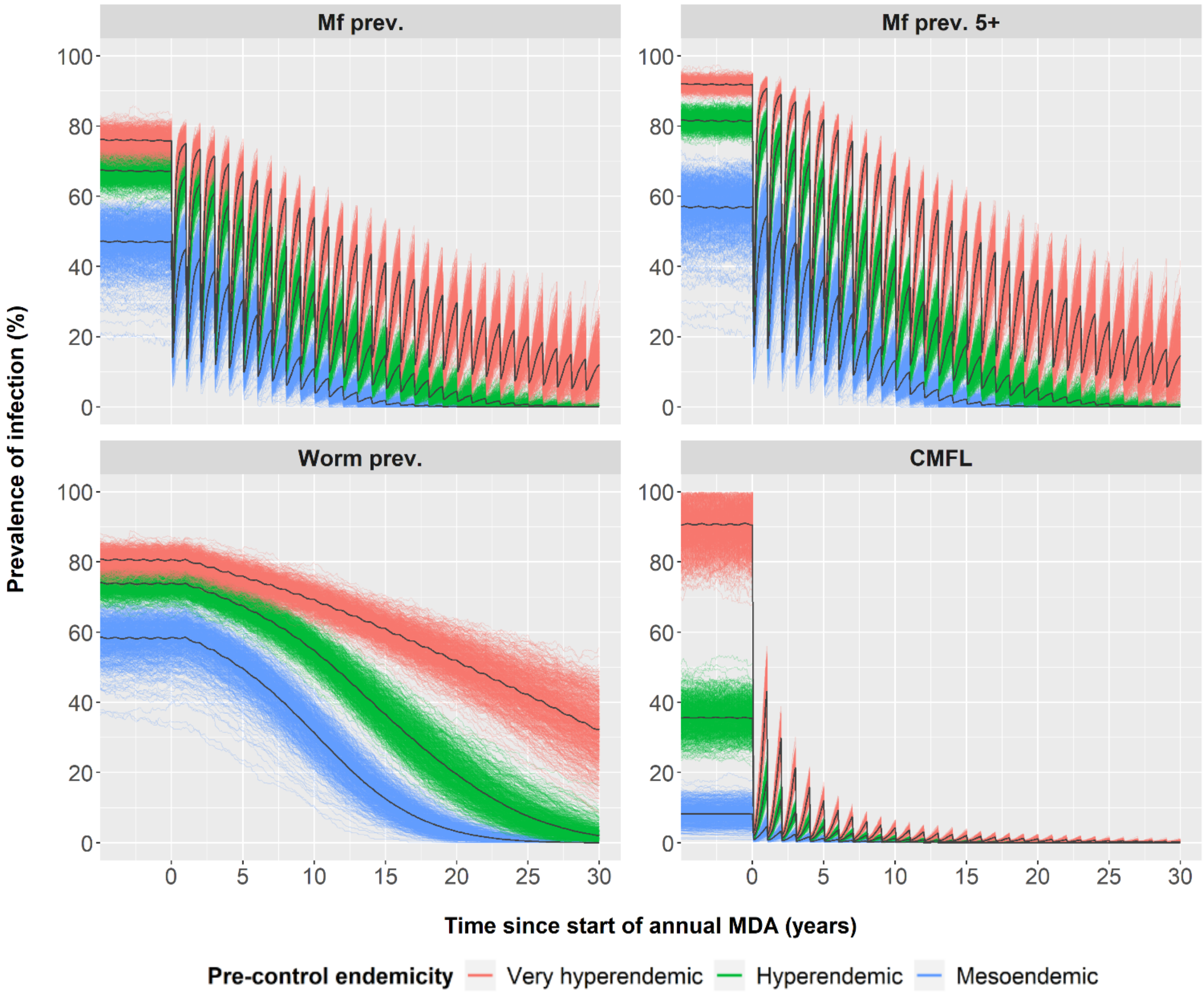

S12 Fig. Stochastic variation of the model output of various subtypes of onchocercal skin disease for the scenario of annual MDA with 70% treatment coverage.

Stochastic variation in the prevalence of mf and morbidity due to OSD by endemicity strata and time since start of MDA in years, with annual treatment coverage of 70%. There is a low predicted morbidity prevalence of atrophy and hanging groin, especially in meso- and hyperendemic areas over time, due to the very low baseline prevalences. The predicted trends are based on 750 simulations.

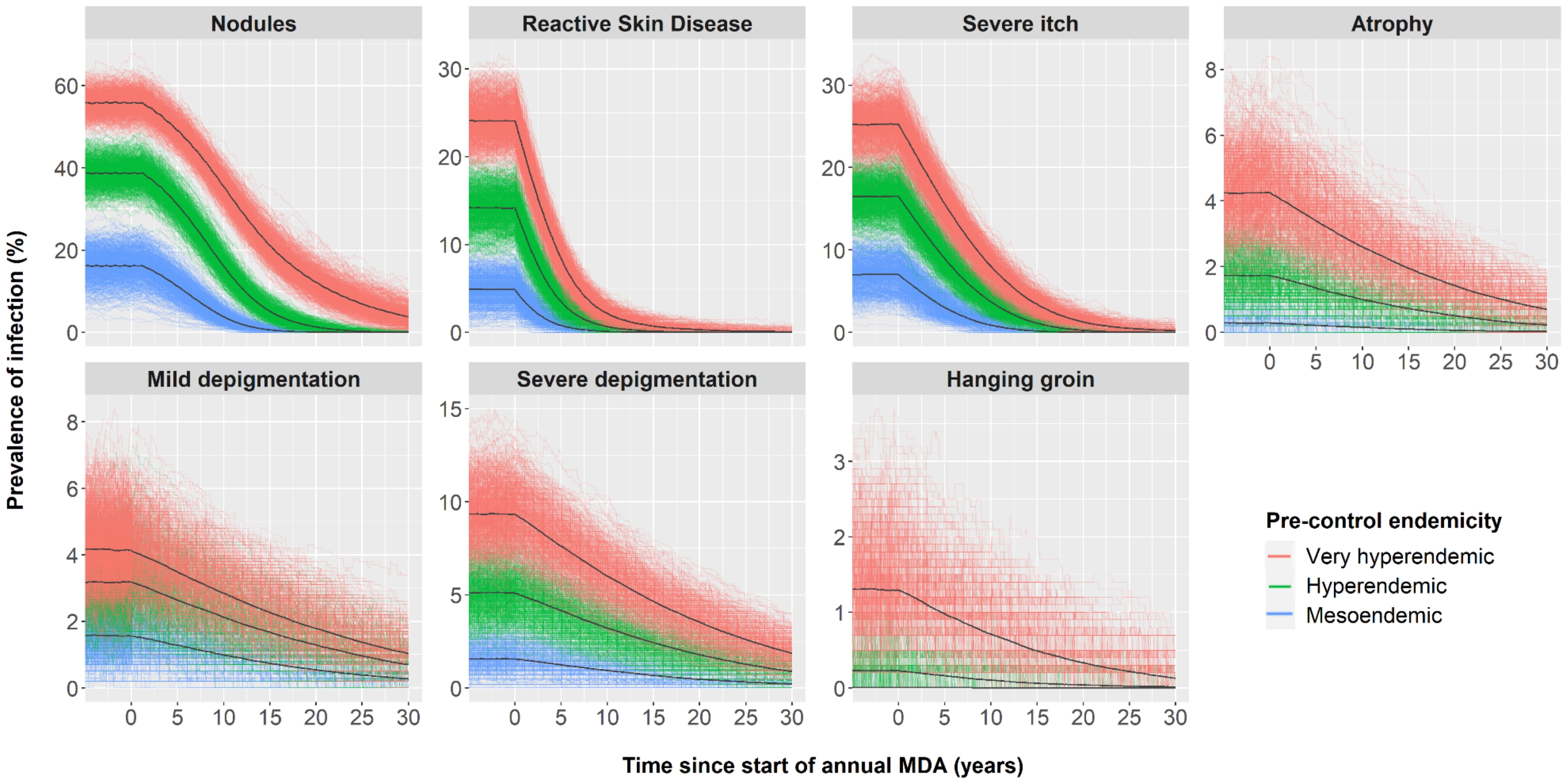

S13 Fig. Stochastic variation of the model output of onchocercal eye disease (OED) due to onchocerciasis (savanna and forest and mixed forest-savanna areas) for the scenario of annual MDA with 70% treatment coverage.

Stochastic variation in the prevalence of mf and morbidity due to eye disease by endemicity strata and time since start of MDA in years, with an annual treatment coverage of 70%. There is a low predicted morbidity prevalence of OED in forest areas due to the very low baseline prevalences, especially in meso- and hyperendemic areas. The predicted trends are based on 750 simulations.

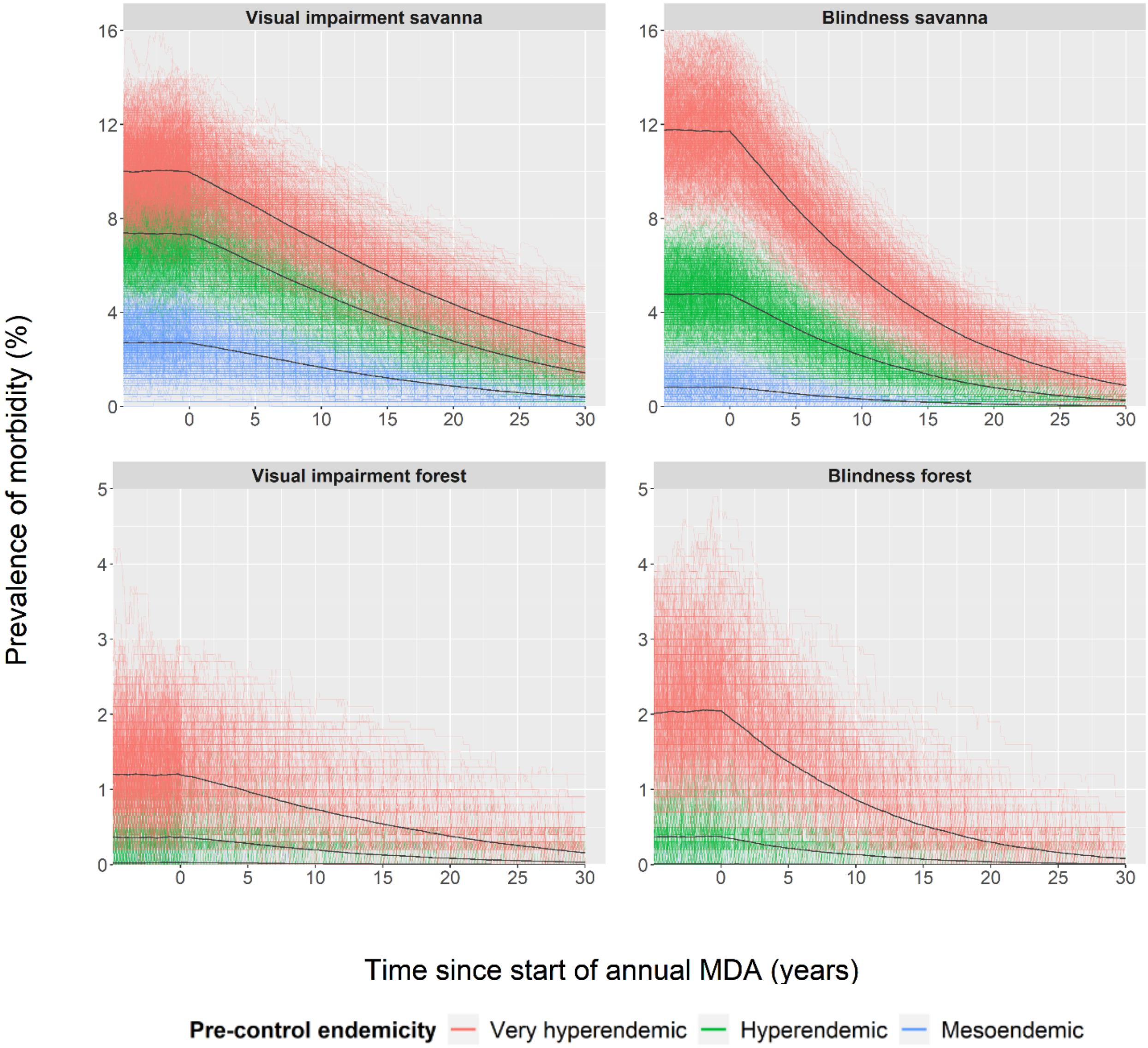

5.2 Re-quantification of the model using alternative biological assumptions

We assessed the sensitivity of our results to two biological assumptions: 1) (ir)reversibility of OED and 2) the level of excess mortality due to blindness. Under several alternative assumptions, we re-quantified disease-specific parameters (S4 Table) and visualise model predictions vs. the data (S14-S16 Figs). Next, we describe each of the alternative assumptions and associated results in more detail.

S4 Table. The fitted values of the damage thresholds for diseases processes for onchocercal eye disease (OED) after re-quantification of the model using alternative biological assumptions. The baseline assumption used in the main analysis is 50% excess mortality with irreversibility of blindness (zero regression). The shape parameter of the gamma distribution for individual susceptibility variability at baseline was estimated at 1.0902, which we rounded off to 1. As we expected that the shape parameter of the gamma distribution for individual susceptibility variability for the alternative assumptions would be the same, we manually set the individual susceptibility variability of the alternative assumptions also to 1.0. Disease thresholds are presented x1000.

Clinical manifestation	Bioclimate *	40% excess mortality due to blindness; zero regression	50% excess mortality due to blindness; zero regression (main analysis)	60% excess mortality due to blindness; zero regression	1% regression of tissue damage leading to blindness; 50% excess mortality
Visual impairment	Savanna	2.10	1.65	1.85	0.57
Blindness		3.30	3.05	2.75	0.85
Visual impairment	Forest	9.40	10.50	8.35	2.35
Blindness		11.00	12.50	9.45	2.50

* Damage threshold parameters for savanna and forest areas were fitted in parallel assuming that variation in individual susceptibility was the same for both bioclimates, disease stages, and as fitted in the main analysis.

Re-quantification of the regression of onchocercal eye disease (OED)

In the main analysis, we assumed that tissue damage in the eye does not regress (solid lines in S14 Fig). Based on this assumption, the model adequately reproduced the association between prevalence of infection and OED in the data. Because some early symptoms of OED like punctate keratitis are known to be (partially) reversible [41,42] in a sensitivity analysis we allowed for eye

damage to regress at an arbitrary rate of 1% per month. To capture that only early stages of OED may be reversible, we only allowed visual impairment – first stage of OED in the model – to be reversible. This meant that if an individual's total damage passed the threshold for visual impairment and then dropped below it again (i.e. because of a temporary stop in damage accrual due to treatment combined with regression of damage) that individual was considered to be free of visual impairment. However, if an individual's total damage passed the threshold for blindness, they were considered blind for the rest of their life, even if the total damage regressed under the threshold for blindness (or even below the threshold for visual impairment).

Impact on infection prevalence

There is a difference in the decline of worm prevalence in the population over time, particularly in very hyperendemic regions (third panel of S25 Fig). This can be explained by the higher prevalence of blindness in higher endemic areas when assuming 1% regression of OAE (based on quantification in forest areas). When there is higher blindness prevalence, there is more excess mortality due to blindness. People with high worm burdens will develop blindness more rapidly and will therefore die more rapidly due to excess mortality, and with the result that the overall prevalence of the worm prevalence declines over time.

Impact on subtypes of OSD prevalence

There are minimal differences between the assumption of no regression and 1% regression per month in the development of OED when assessing the pre-control predicted morbidity prevalence of various subtypes of OSD (based on quantification in forest areas). These little differences that are present are primarily due to stochasticity of the model output, especially when subtypes of OSD are rarer (hanging groin, atrophy) (S25 Fig).

Impact on the prevalence of OED

As expected, the assumption of (partially) reversible OED resulted in generally lower predicted prevalences of visual impairment (S26 Fig). It further resulted in higher predicted prevalences of blindness in hyperendemic areas. This pattern reflects that with increasing endemicity level, the regression rate of damage is outpaced by the speed of damage accrual such that more individuals accrue damage to a point where they develop irreversible blindness. At mesoendemic levels, damage

accrual is slower on average, allowing for more regression of damage and fewer cases of visual impairment, resulting slightly lower prevalences of OED than in the main analysis.

S14 Fig. Model-predicted association between the prevalence of infection and onchocercal eye disease (OED) with a 1% regression of tissue damage leading to blindness as compared to the baseline assumption of no regression.

The figure shows the pre-control association between the community-level prevalence of infection (x-axis) and prevalence of OED (y-axis) with the alternative assumption (in the absence of treatment) (dashed line) as compared to the baseline assumption of no regression in tissue accumulation (straight line) for savanna (panel A) and forest (panel B) areas. Disease parameters were newly fitted using the pre-control data on the association between the prevalence of infection and OED.

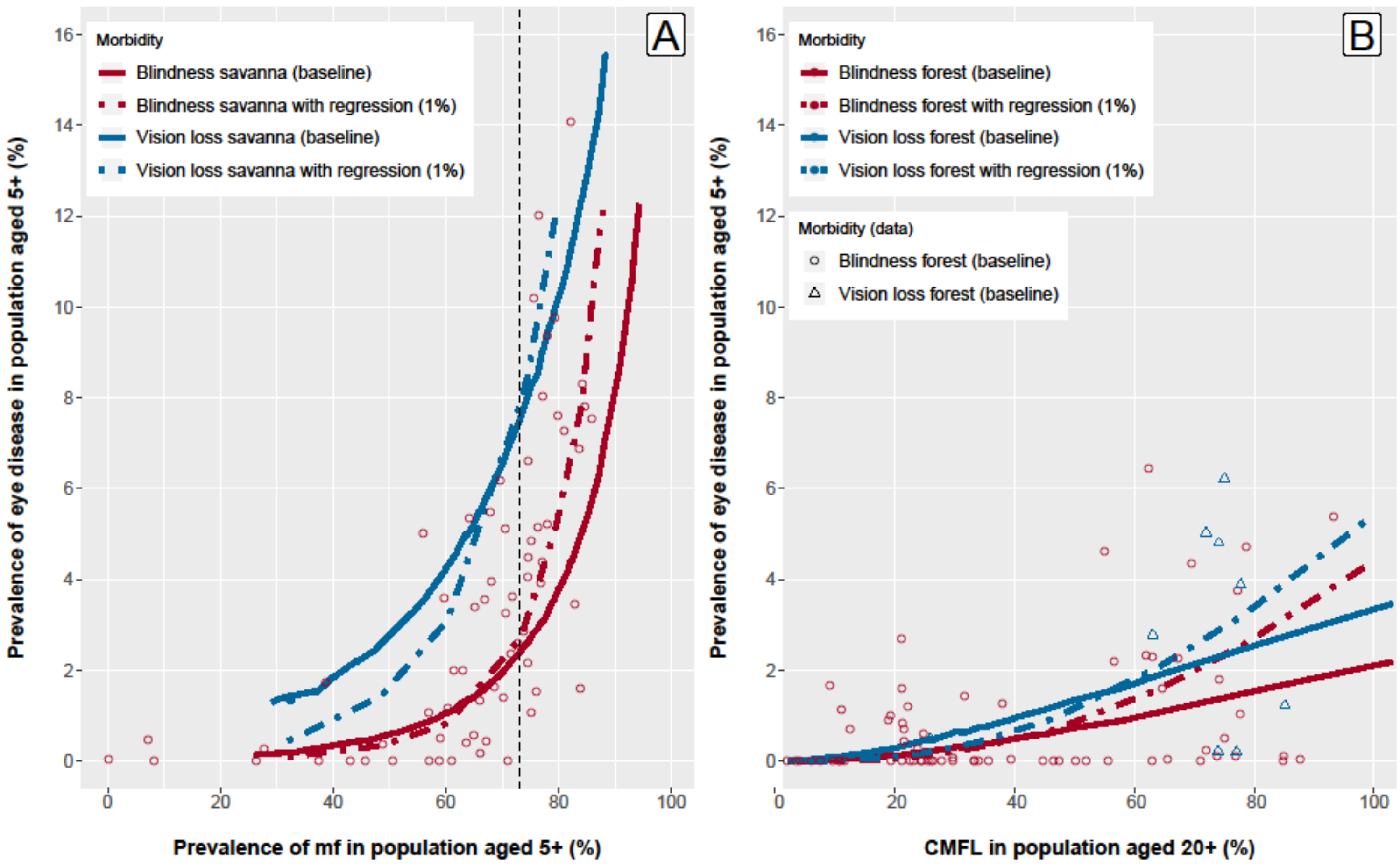

Re-quantification of excess mortality due to blindness

In the main analysis, we further assumed that individuals who turn blind lose 50% of their remaining life expectancy, which was validated against data on declining trends in prevalence in blindness in former OCP countries in a period when control consisted exclusively of vector control [5]. The sensitivity of this assumption was assessed by setting the reduction in remaining life expectancy to 40% (S15 Fig) or 60% (S16 Fig) instead of 50%. We performed the SSE grid with a large number of randomly chosen initial parameters in order to avoid the risk of a local minimum, yet the disease thresholds return repeatedly to the same disease thresholds used in this model. Both alternative assumptions increased the curvature of the association between prevalence of infection and OED, which is due to a shift in the estimated damage threshold for blindness (which compensates for the shorter or longer remaining lifespan of prevalent blind cases). The parameter for individual variation in susceptibility to disease was set to 1.0, similar to the baseline assumption to stabilise the curve of the association.

Impact on infection prevalence

The various assumptions on excess mortality do not influence the pre-control levels of subtypes of infection nor the trends over time, as we would expect (S27 Fig).

Impact on subtypes of OSD prevalence

Similarly, these assumptions barely impact the predicted trends in prevalence of subtypes of OSD except for hanging groin (S29 Fig). Hanging groin has the lowest predicted morbidity prevalence and is therefore subject to more stochastic variation in the model predictions (a high number of seed numbers are required to predict a somewhat stable morbidity prevalence over time). The lower prevalence of hanging groin with 60% excess mortality in very hyperendemic areas could therefore be a chance outcome.

Impact on vision loss

S29 Fig shows that the predicted pre-control OED prevalence of 40% and 60% excess mortality are quite similar (in most situations, the prevalence of OED is higher for both 40% and 60% excess mortality as compared to the baseline assumption, except for visual impairment in savanna areas). We would have expected throughout the four panels to see a chronological order in the pre-control OED prevalence and trends over time of 40% excess mortality (most surviving blind cases, thus

highest blindness prevalence), 50% excess mortality (average of 40% and 60% excess mortality), and then 60% excess mortality (most rapidly dying people that turned blind will die more rapidly, with an overall lower OAE prevalence). As explained, this is due to due to a shift in the estimated damage threshold for blindness.

S15 Fig. Model-predicted association between the prevalence of infection and onchocercal eye disease (OED) assuming a 40% or 50% (baseline) reduction in remaining life expectancy due to blindness.

The figure shows the pre-control association between the community-level prevalence of infection (x-axis) and prevalence of OED (y-axis) with the alternative assumption (dashed line) as compared to the baseline assumption of 50% reduction in remaining life expectancy due to onchocercal blindness (straight line) for savanna (panel A) and forest (panel B) areas. Disease parameters were newly fitted using the pre-control data on the association between the prevalence of infection and OED.

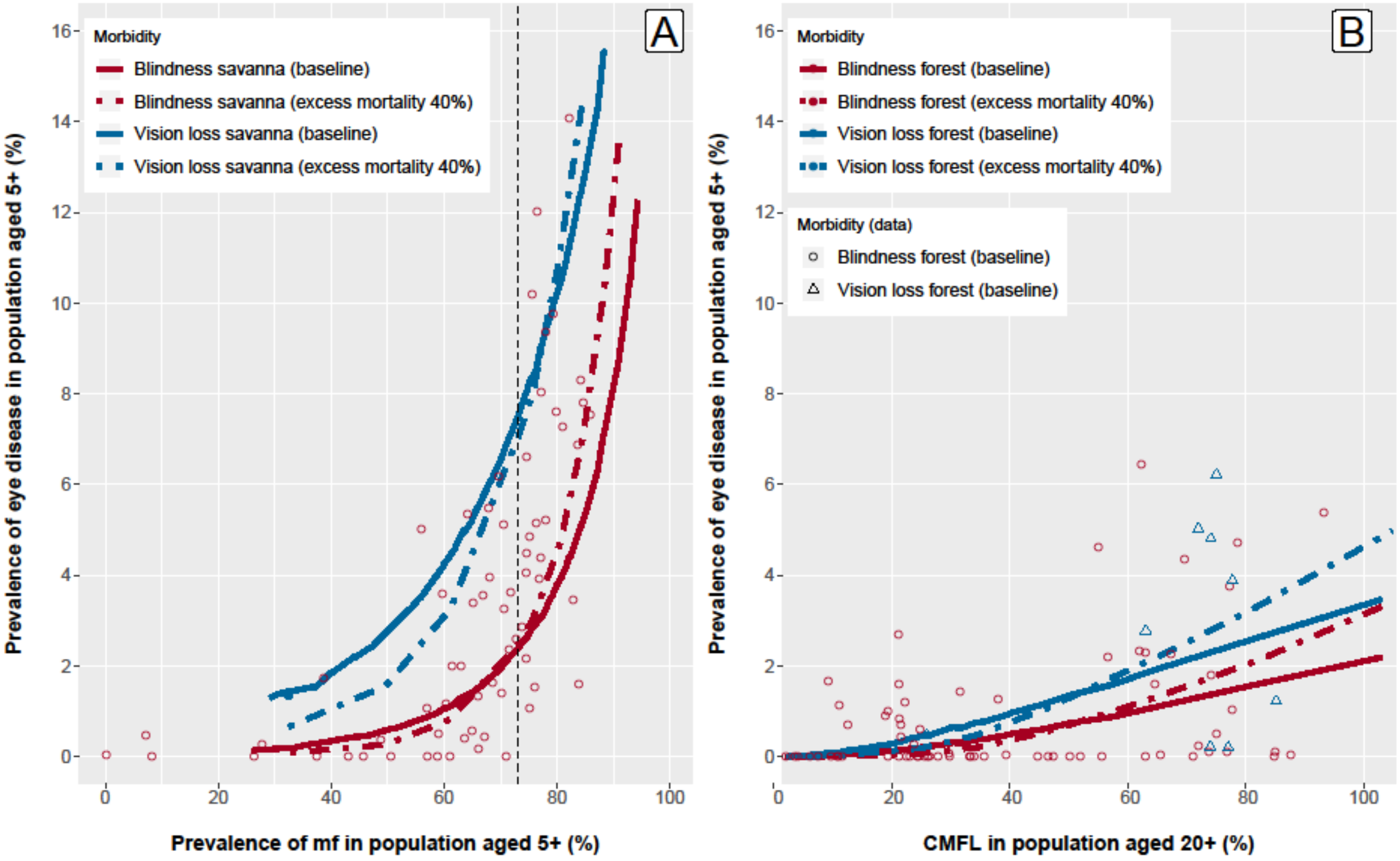

S16 Fig. Goodness-of-fit of the association between the prevalence of infection and onchocercal eye disease (OED) with a 60% reduction in remaining life expectancy due to blindness as compared to the baseline assumption of a 50% reduction in remaining life expectancy.

The figure shows the pre-control association between the community-level prevalence of infection (x-axis) and prevalence of OED (y-axis) with the alternative assumption (dashed line) as compared to the baseline assumption of 50% reduction in remaining life expectancy due to onchocercal blindness (straight line) for savanna (panel A) and forest (panel B) areas. Disease parameters were newly fitted using the pre-control data on the association between the prevalence of infection and OED.

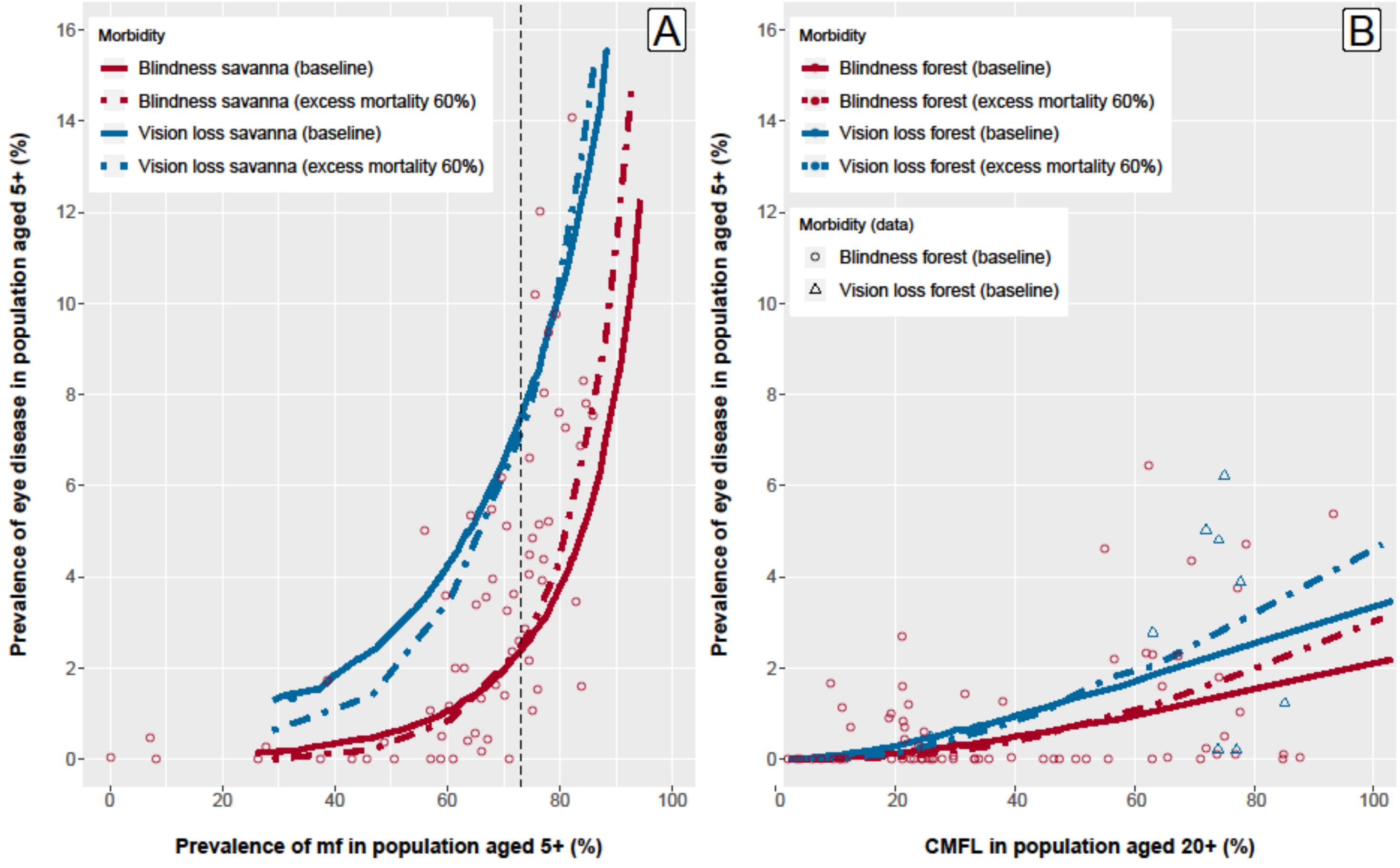

5.3 Results of the sensitivity analysis

We assessed the impact of various programmatic variations for a wide variety of epidemiological settings. The impact of MDA with varying MDA coverages on infection and morbidity prevalence (S17 Fig, and Fig 3 of the main manuscript) as well as the impact of MDA treatment frequency (annual versus semi-annual MDA) (S18-S20 Figs) was assessed. S17 and S18 Figs show that the prevalence of infection declines rapidly, but with considerable peaks, which is caused by resumption of mf reproduction by adult worms in infected individuals between treatments. Sufficient time is required for infection levels to drop to zero levels, due to the long lifespan of adult worms (an assumed ~10-15 years), in which time adult worms will recover and produce mf after an initial post-treatment arrest of mf production. Higher systematic non-participation of MDA barely influenced the prevalence of disease over time (S22-S24 Figs).

We also assessed how MDA influences the prevalence of morbidity over time with various model-inherent assumptions through univariate sensitivity analyses. In order to assess the biological assumption of reversibility of OED and excess mortality due to blindness, we quantified the model again to obtain disease-specific parameters fitting best to the available data. Changing such model-inherent assumptions (e.g. reversibility of blindness, excess mortality due to blindness) changes the pre-control association between infection and OED considerably (S14-S16 Figs). Consequently, time trends on the prevalence of infection and morbidity also vary much between the applied assumptions, as shown in S24-S29 Figs.

Alternative MDA population coverages

S17 Fig. Predicted impact of annual mass drug administration on the prevalence of infection with varying scenarios of treatment coverage ranging from 60%, 70% and 80%.

Different column panels represent the different endemicity strata, while the row panels represent the various outcome variables (infection matrices). The different coloured lines in the panels represent the various scenarios of treatment coverage. The predicted trends are based on the average of 750 simulations. For the first three types of infection matrices, the level of infection is the community-based prevalence in the population. For the last type of infection matric, the level of infection is the geometric mean number of mf per person (Community Microfilarial Load [CMFL]). Abbreviations: Mf prev. = Microfilariae prevalence, Mf prev. 5+ = Mf prevalence in age groups ≥5 years, Worm prev. = Adult worm prevalence, CMFL = Community microfilarial load.

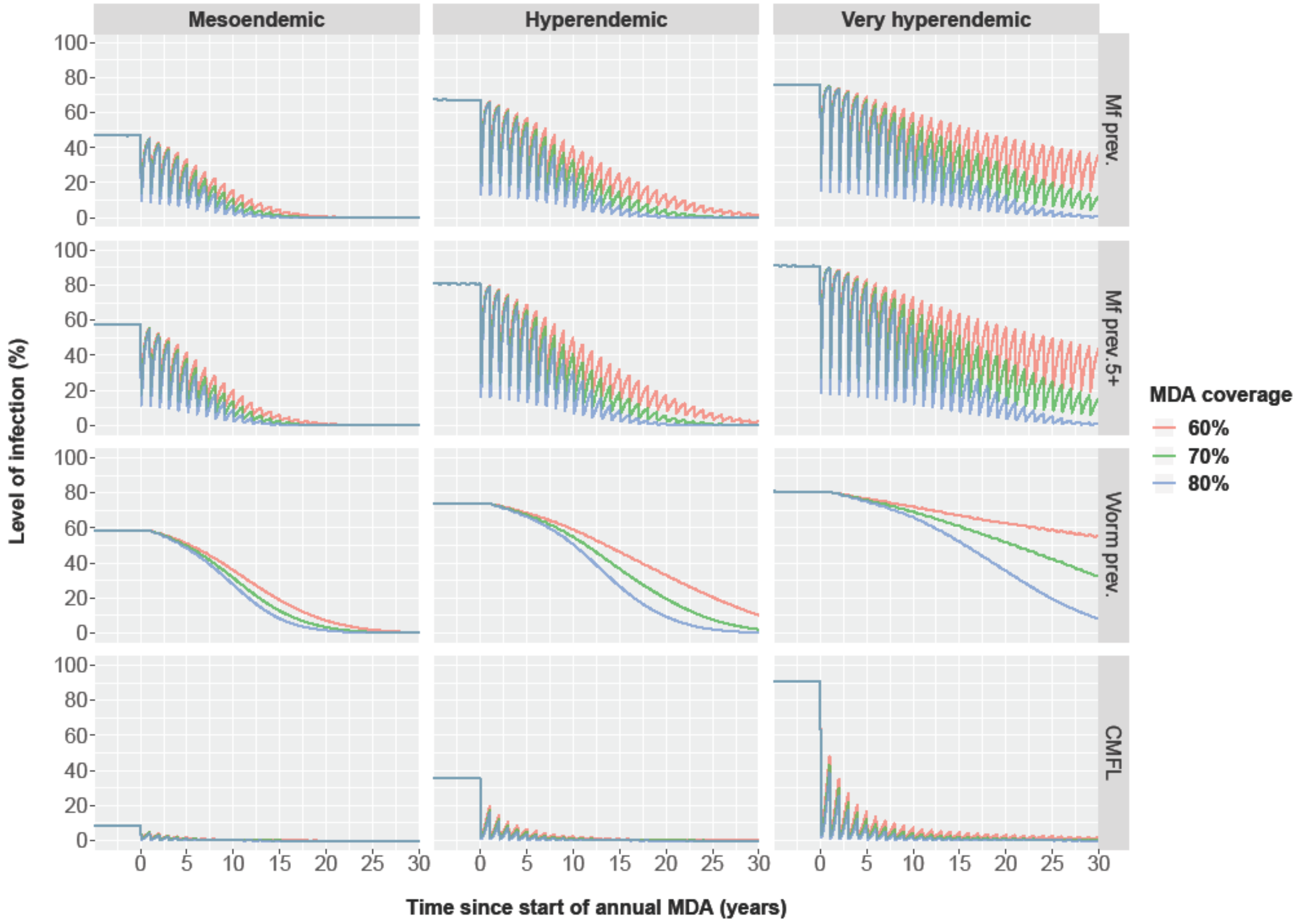

Alternative MDA implementation frequencies with 70% population coverage

S18 Fig. Predicted impact of annual versus semi-annual mass drug administration with 70% population coverage on the prevalence of infection.

Different column panels represent the different endemicity strata, while the row panels represent the various outcome variables (infection matrices). The different coloured lines in the panels represent the various treatment frequencies of MDA with 70% population treatment coverage. The predicted trends are based on the average of 750 simulations. For the first three types of infection matrices, the level of infection is the community-based prevalence in the population. For the last type of infection matric, the level of infection is the geometric mean number of mf per person (Community Microfilarial Load [CMFL]). Abbreviations: Mf prev. = Microfilariae prevalence, Mf prev. 5+ = Mf prevalence in age groups ≥5 years, Worm prev. = Adult worm prevalence, CMFL = Community microfilarial load.

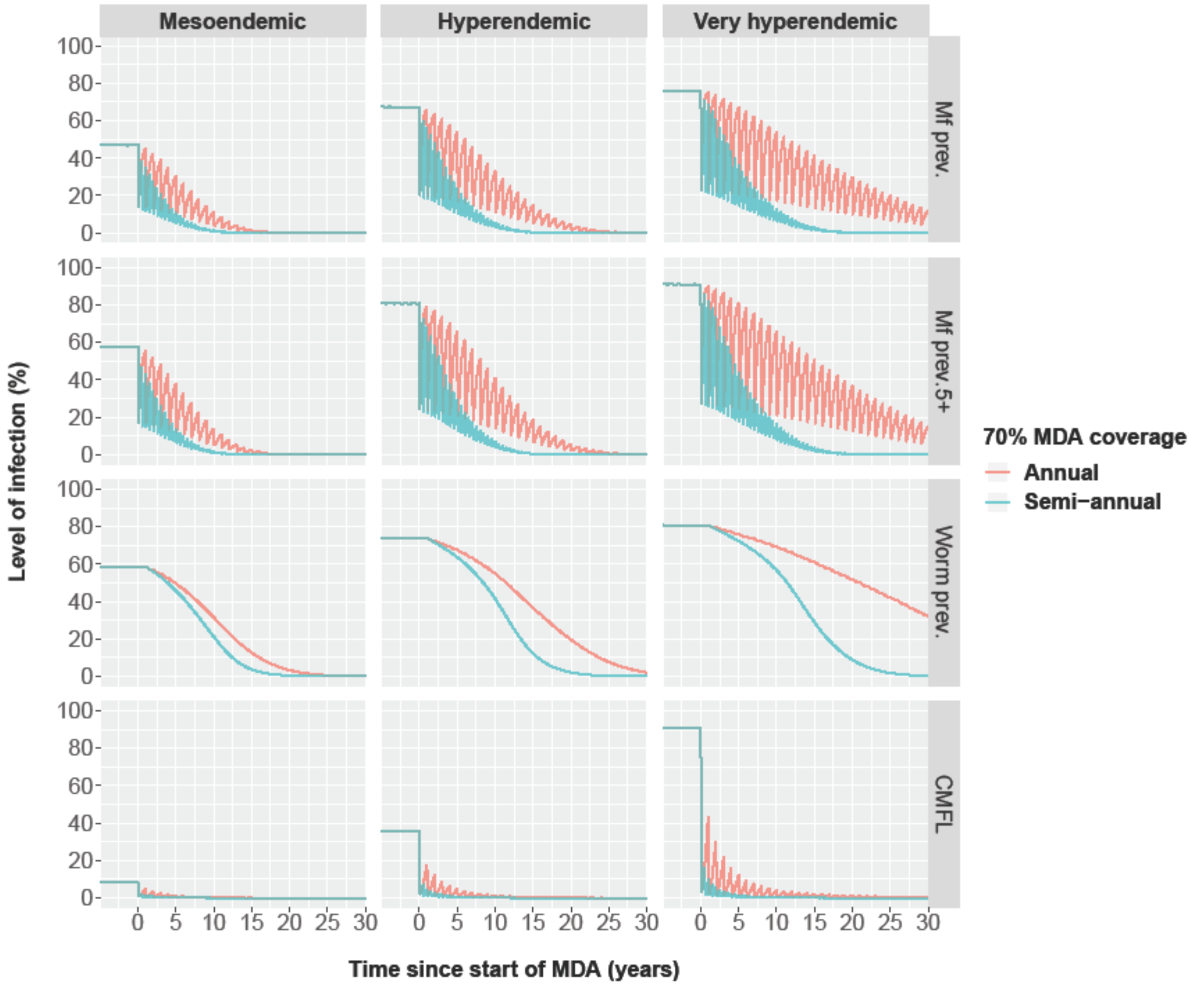

S19 Fig. Predicted impact of annual versus semi-annual mass drug administration with 70% population coverage on the prevalence of subtypes of OSD.

Coloured lines represent different treatment frequencies at 70% MDA population coverage, and different line types represent the various pre-control endemicity levels. Different panels represent the various subtypes of OSD. The predicted trends are based on the average of 750 simulations. Please note the different scales for the y-axes in the panels.

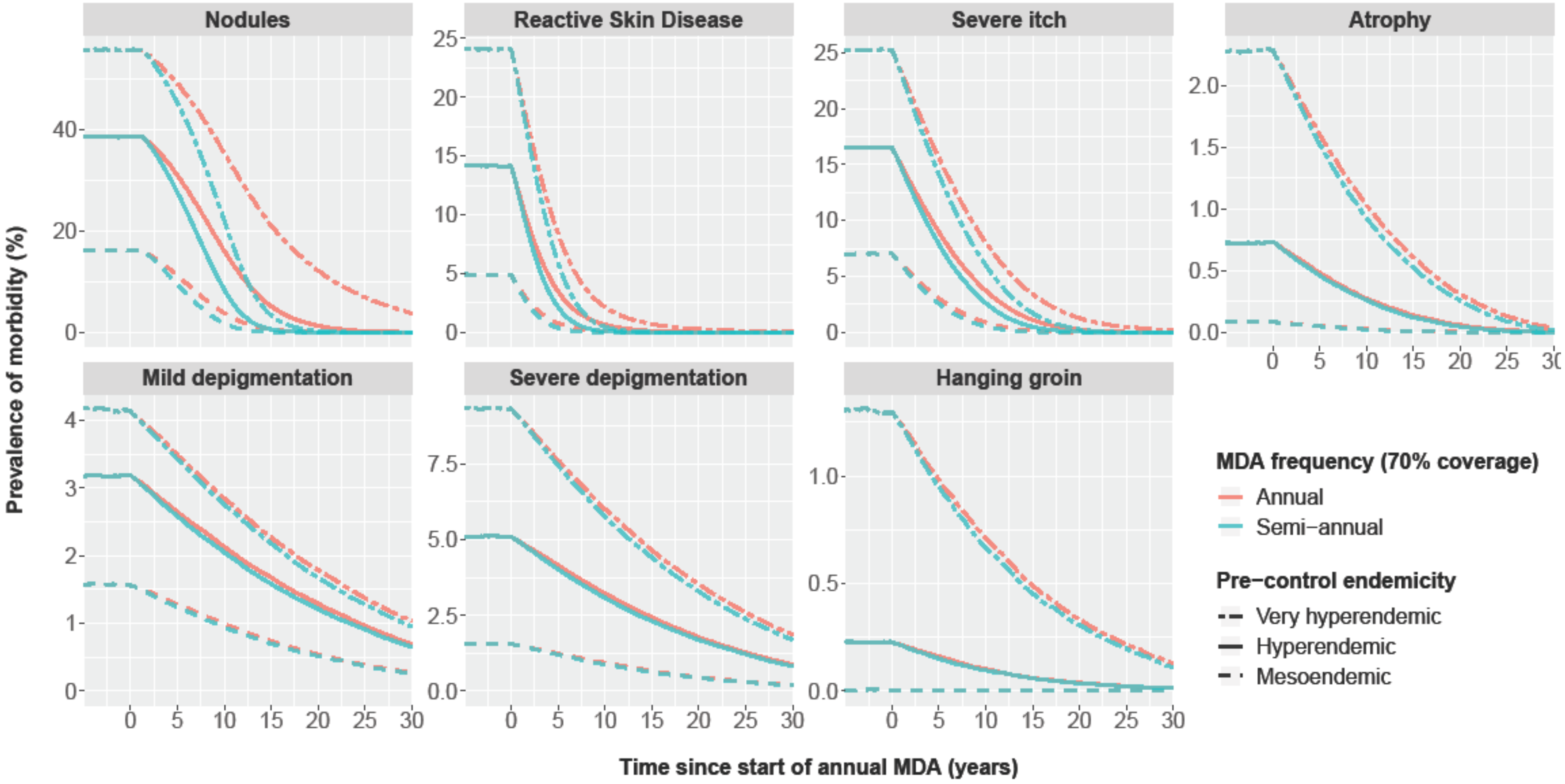

S20 Fig. Predicted impact of annual versus semi-annual MDA with 70% population coverage on the prevalence of onchocercal eye disease (OED) in savanna and forest areas.

Coloured lines represent different treatment frequencies at 70% MDA population coverage, and different line types represent the various pre-control endemicity levels. Different panels represent the various subtypes of OED in forest and savanna areas. The predicted trends are based on the average of 750 simulations. Please note the different scales for the y-axes in the panels.

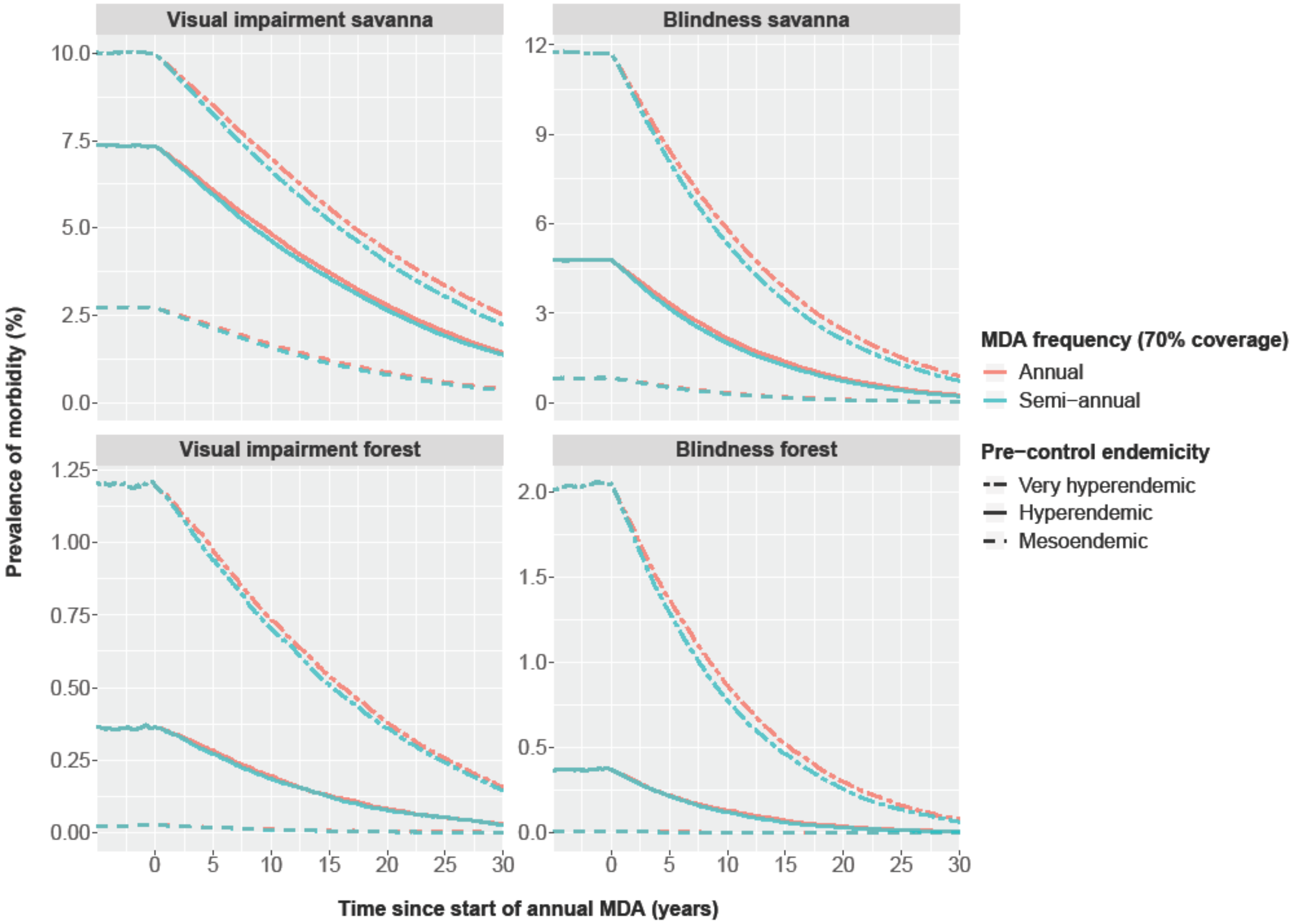

Alternative levels of systematic non-participation to MDA

S21 Fig. Predicted impact of annual mass drug administration (70% treatment coverage) on the prevalence of infection with varying systematic non-participation to ivermectin.
Different column panels represent the different endemicity strata, while the row panels represent the various outcome variables (infection metrics). The different coloured lines in the panels represent three scenarios for systematic non-compliance to ivermectin. As there are barely any differences in the predicted prevalence of infection with varying systematic non-participation, one line colour can best be seen. The predicted trends are based on the average of 750 simulations. For the first three types of infection matrices, the level of infection is the community-based prevalence in the population. For the last type of infection matric, the level of infection is the geometric mean number of mf per person (Community Microfilarial Load [CMFL]). Abbreviations: Mf prev. = Microfilariae prevalence, Mf prev. 5+ = Mf prevalence in age groups ≥5 years, Worm prev. = Adult worm prevalence, CMFL = Community microfilarial load.

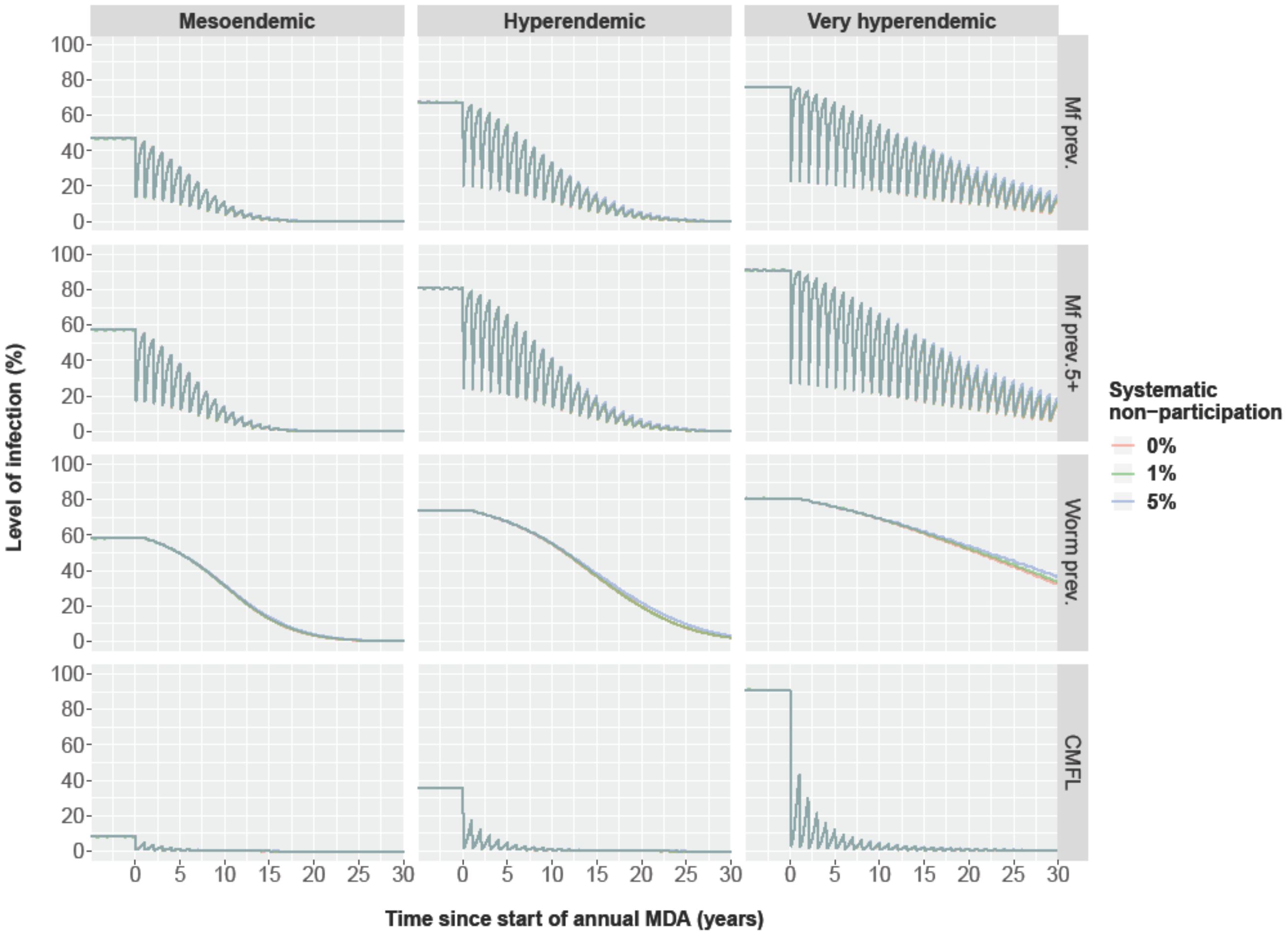

S22 Fig. Predicted impact of annual MDA on the prevalence of various subtypes of OSD with varying systematic non-participation to ivermectin.

Coloured lines represent different levels of systematic non-participation to annual MDA with 70% treatment coverage, and different line types represent the various pre-control endemicity levels. Different panels represent the various subtypes of OSD. The predicted trends are based on the average of 750 simulations. Please note the different scales for the y-axes in the panels.

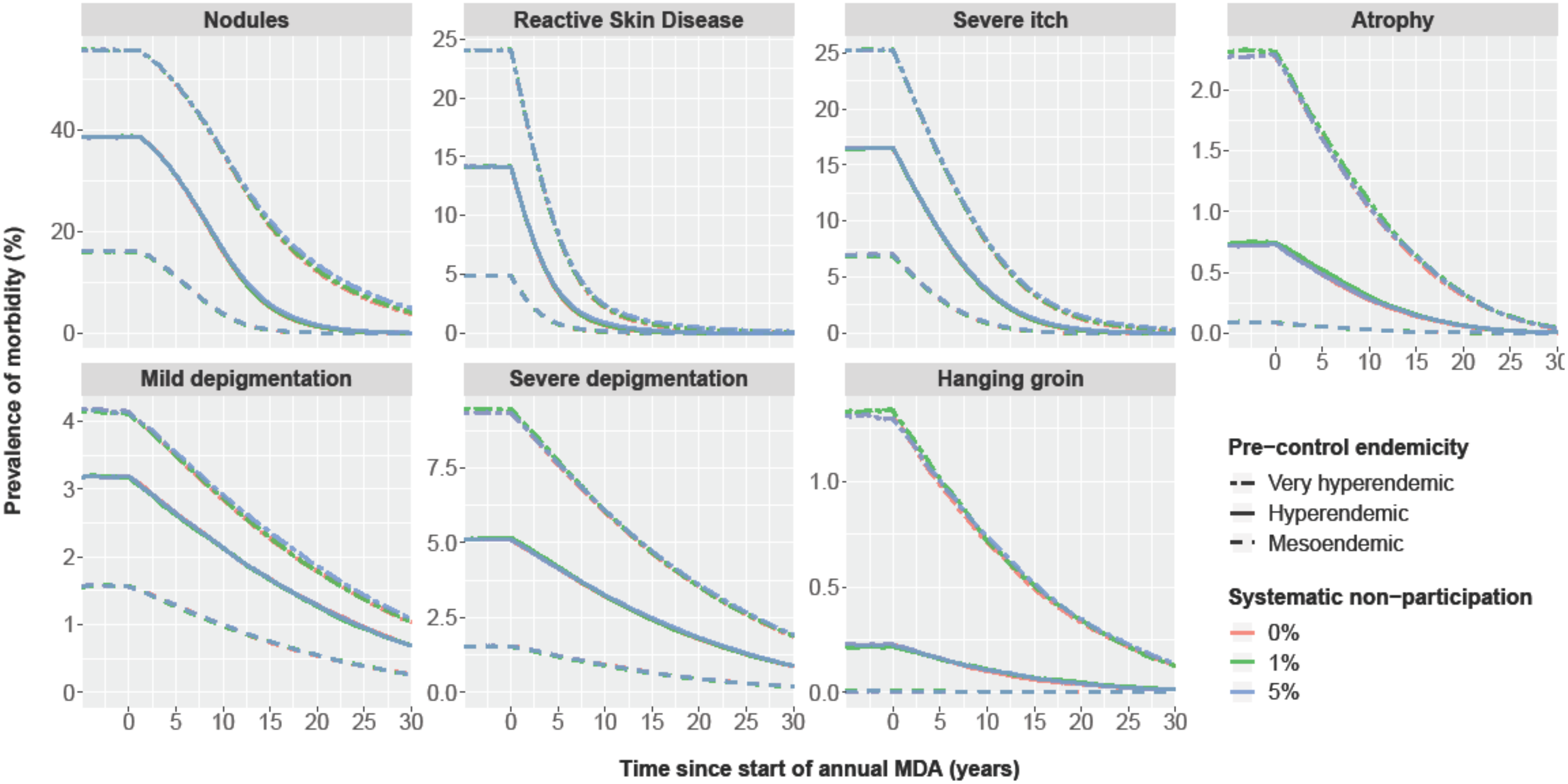

S23 Fig. Predicted impact of annual MDA on the prevalence of onchocercal eye disease (OED) due to onchocerciasis with varying systematic non-participation to ivermectin.

Coloured lines represent different levels of systematic non-participation to annual MDA with 70% treatment coverage, and different line types represent the various pre-control endemicity levels. Different panels represent the various subtypes of OED in forest and savanna areas. The predicted trends are based on the average of 750 simulations. Please note the different scales for the y-axes in the panels.

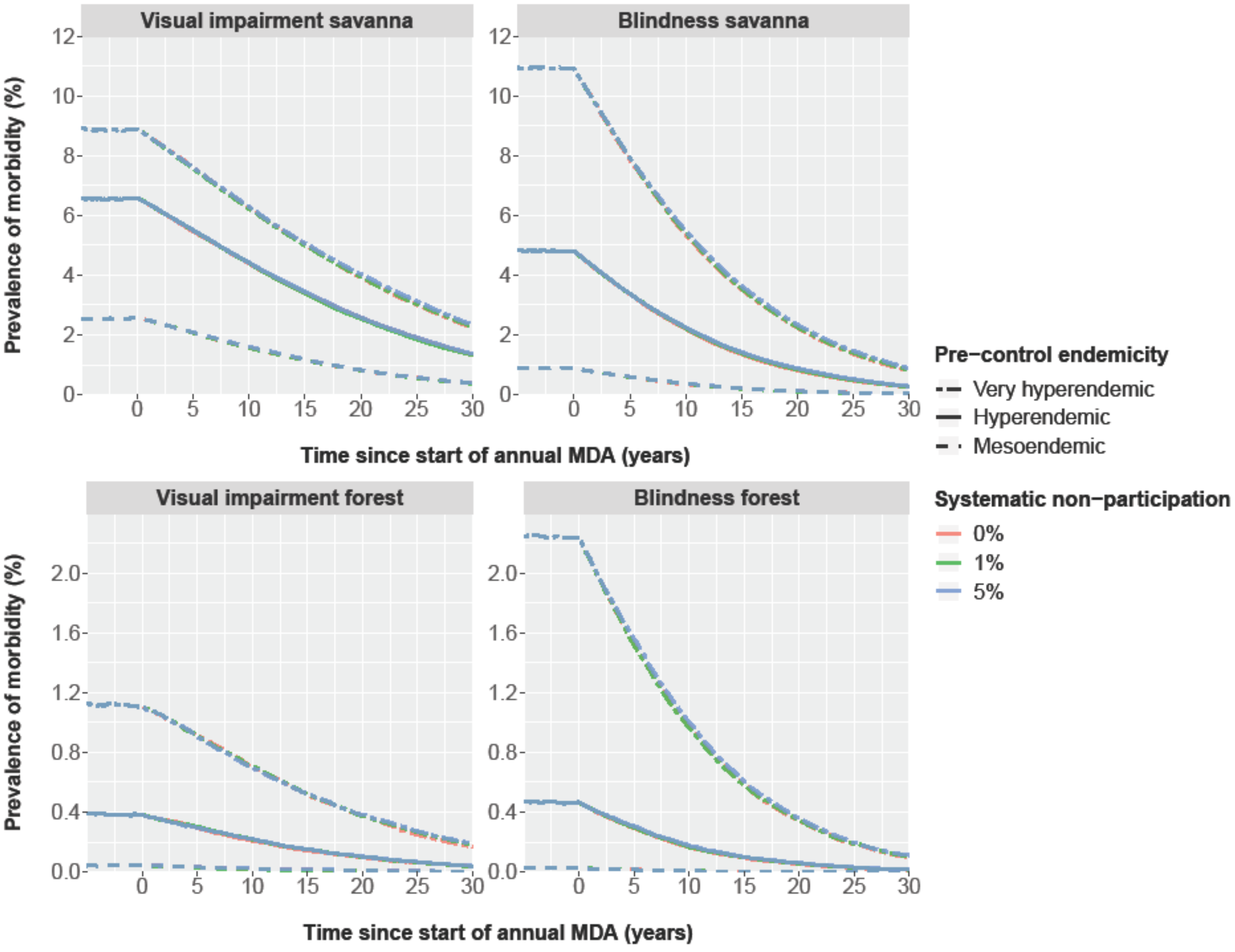

Alternative assumptions regarding the regression of onchocercal eye disease (OED)

S24 Fig. Predicted impact of annual MDA (70% treatment coverage) on the prevalence of infection with and without regression in tissue damage leading to blindness.

Different column panels represent the different endemicity strata, while the row panels represent the various outcome variables (infection metrics). The different coloured lines in the panels represent 1% reversibility (red) and irreversibility (blue, baseline assumption). As there are barely any differences in the predicted prevalence of infection with or without regression of tissue damage, one line colour can best be seen. The predicted trends are based on the average of 750 simulations. For the first three types of infection matrices, the level of infection is the community-based prevalence in the population. For the last type of infection matric, the level of infection is the geometric mean number of mf per person (Community Microfilarial Load [CMFL]). Abbreviations: Mf prev. = Microfilariae prevalence, Mf prev. 5+ = Mf prevalence in age groups ≥5 years, Worm prev. = Adult worm prevalence, CMFL = Community microfilarial load.

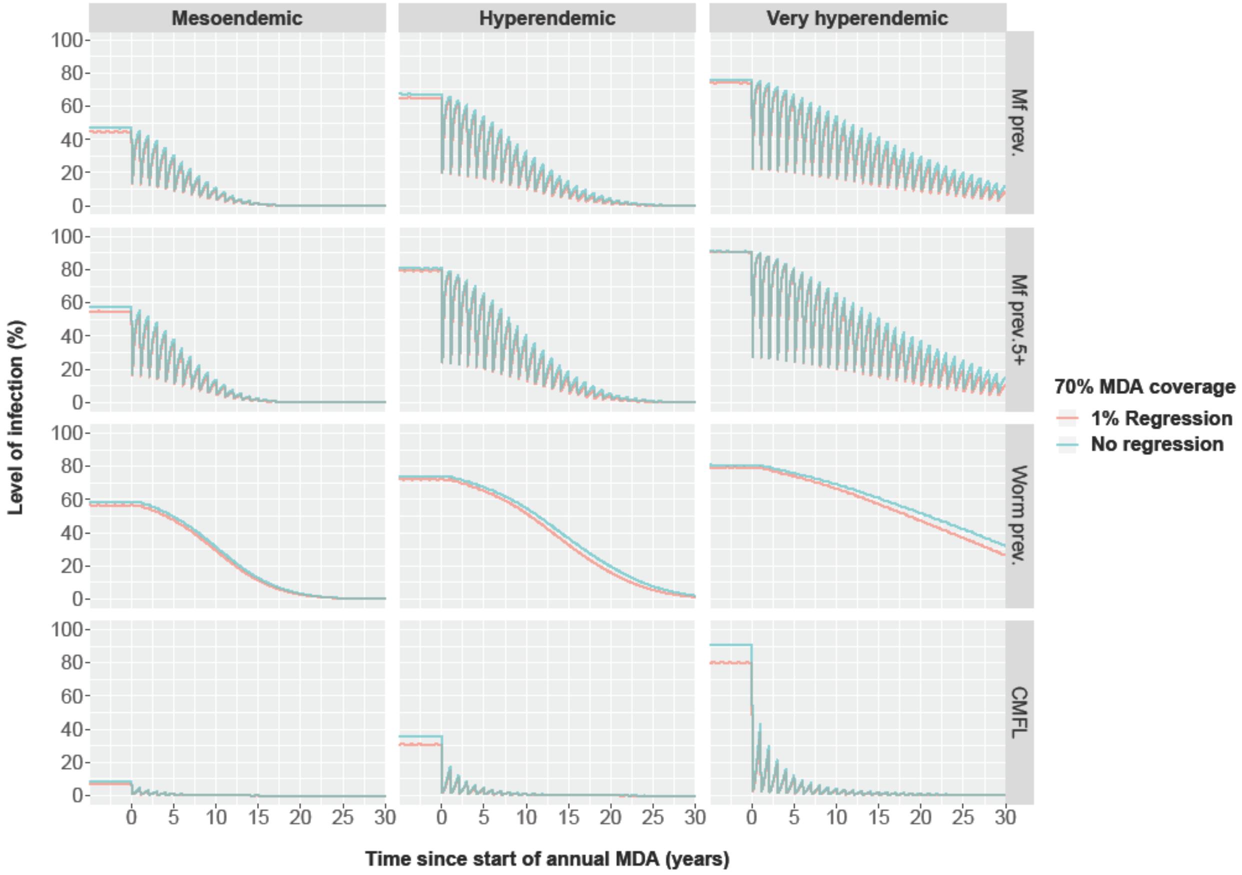

S25 Fig. Predicted impact of annual MDA (70% treatment coverage) on the prevalence of various subtypes of OSD with and without regression in tissue damage leading to blindness. Different column panels represent the different endemicity strata, while the row panels represent the various outcome variables (subtypes of OSD). The different coloured lines in the panels represent 1% reversibility (red) and irreversibility (blue, baseline assumption). The predicted trends are based on the average of 750 simulations. Due to some differences in pre-control morbidity levels (S12 Fig), the trends in prevalence of subtypes of OSD over time is somewhat different between the baseline and alternative assumption on regression in tissue damage leading to blindness. Please note the different scales for the y-axes in the panels.

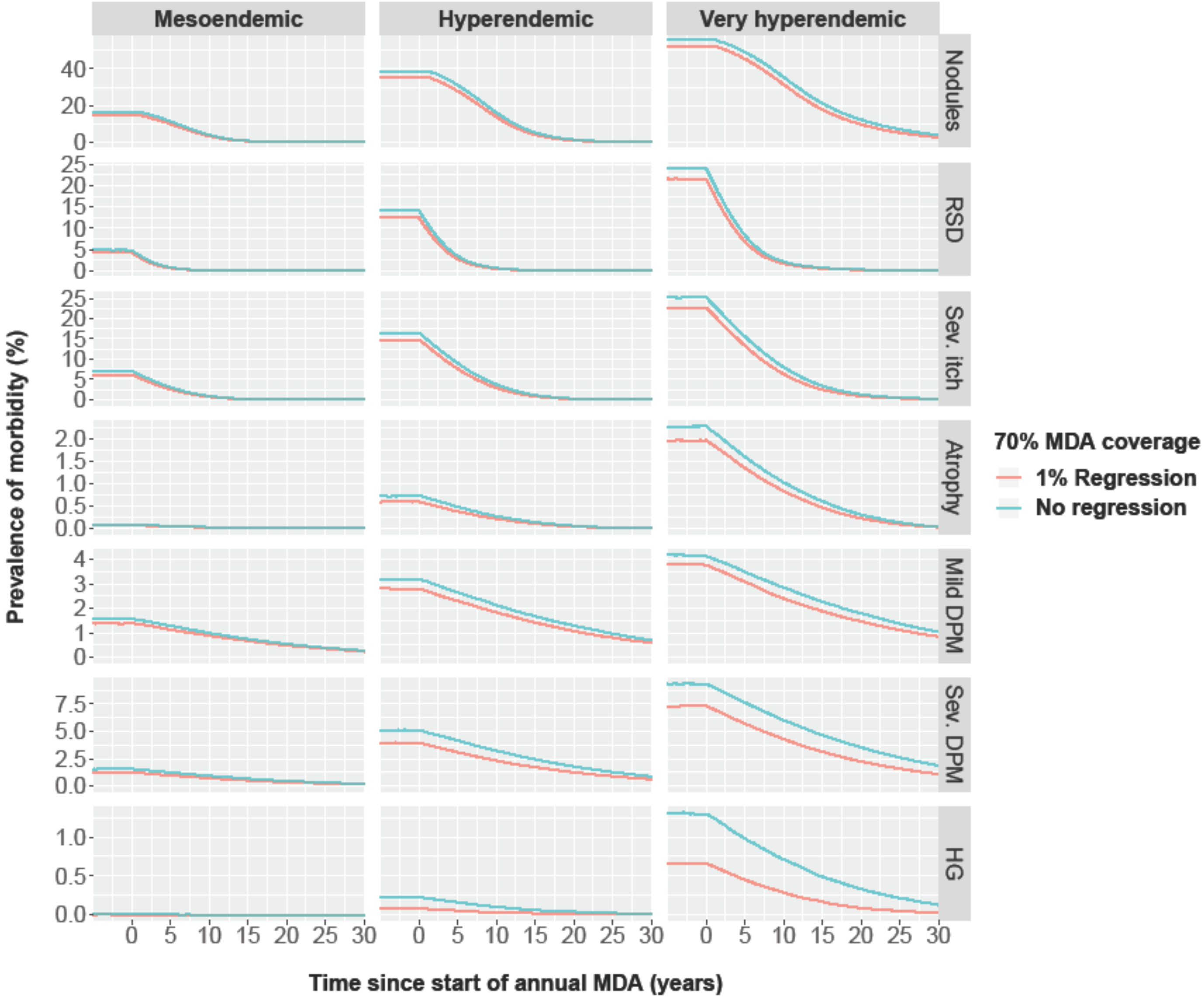

S26 Fig. Predicted impact of annual MDA (70% treatment coverage) on onchocercal eye disease (OED) with some level of regression in tissue damage leading to blindness.

The different coloured lines in the panels represent 1% reversibility (red) and irreversibility (blue, baseline assumption). Different line types represent the various pre-control morbidity prevalence levels. Different panels represent the various subtypes of OED in savanna (upper panels) and forest (lower panels) areas. The predicted trends are based on an average of 750 simulations. Please note the different scales for the y-axes in the panels.

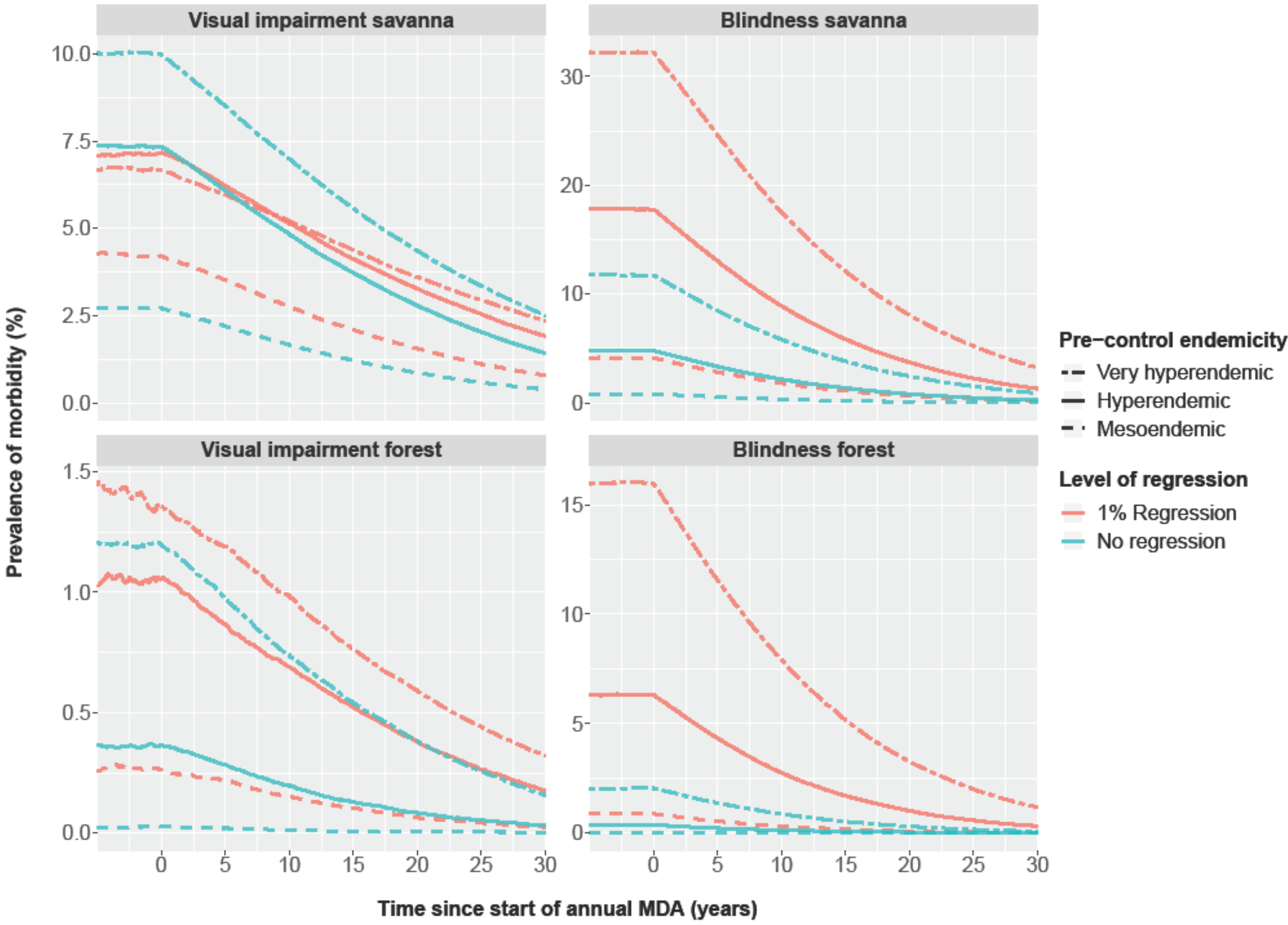

Alternative assumptions regarding excess mortality due to blindness

S27 Fig. Predicted impact of annual MDA (70% treatment coverage) on *O. volvulus* infection with varying excess mortality due to onchocercal blindness.

Different column panels represent the different endemicity strata, while the row panels represent the various outcome variables (infection metrics). The different coloured lines in the panels represent the various scenarios for excess mortality due to blindness (50% is our baseline assumption), but are barely seen as there are no differences at all in the predicted prevalence of morbidity with varying excess mortality. The predicted trends are based on the average of 750 simulations. For the first three types of infection matrices, the level of infection is the community-based prevalence in the population. For the last type of infection matric, the level of infection is the geometric mean number of mf per person (Community Microfilarial Load [CMFL]). Abbreviations: Mf prev. = Microfilariae prevalence, Mf prev. 5+ = Mf prevalence in age groups ≥5 years, Worm prev. = Adult worm prevalence, CMFL = Community microfilarial load.

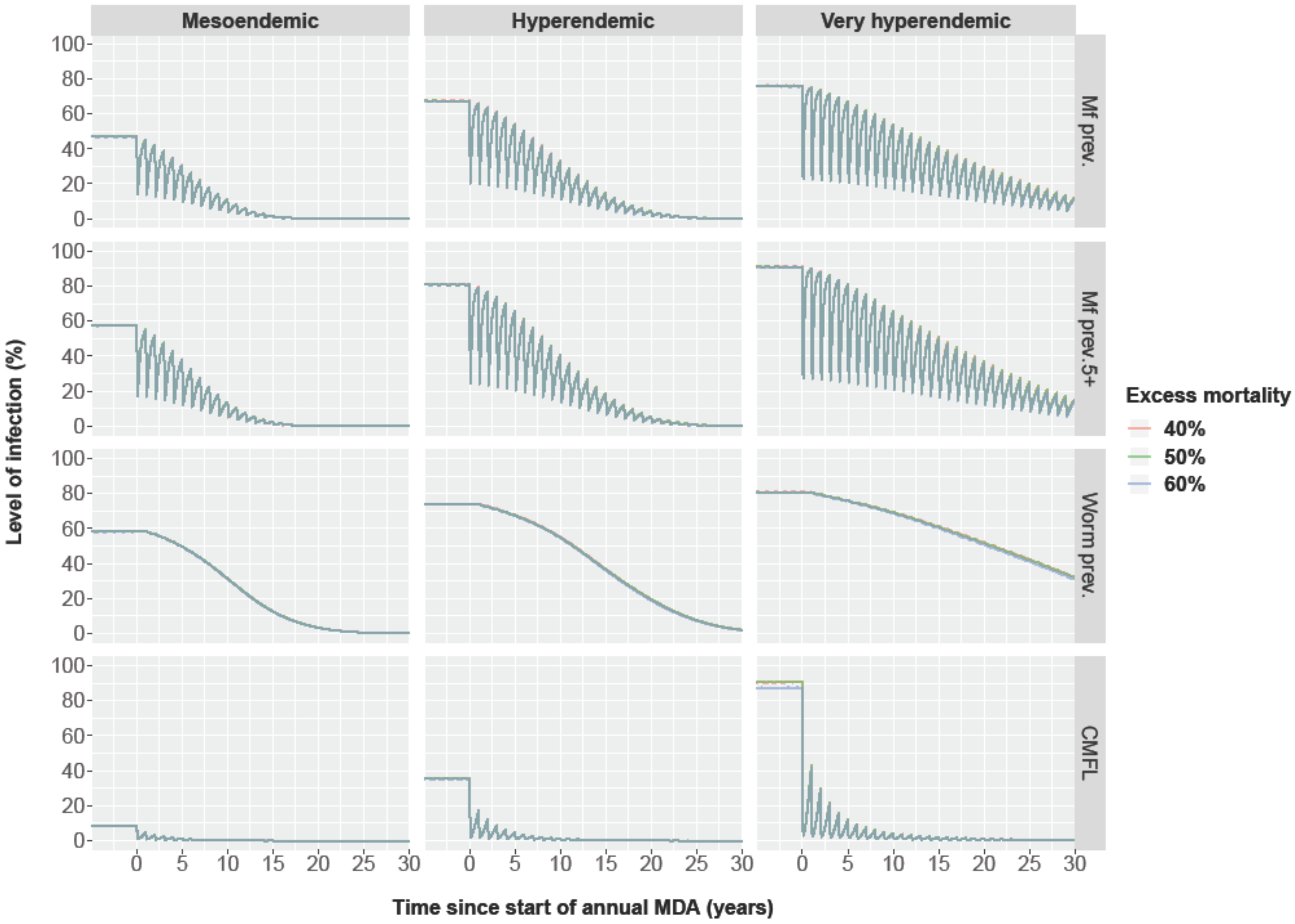

S28 Fig. Predicted impact of annual MDA (70% treatment coverage) on various subtypes of OSD with varying excess mortality due to onchocercal blindness.

Coloured lines represent different percentages of excess mortality at 70% population coverage of annual MDA, and different line types represent the various pre-control endemicity levels. Different panels represent the various subtypes of OSD. The predicted trends are based on the average of 750 simulations. Please note the different scales for the y-axes in the panels.

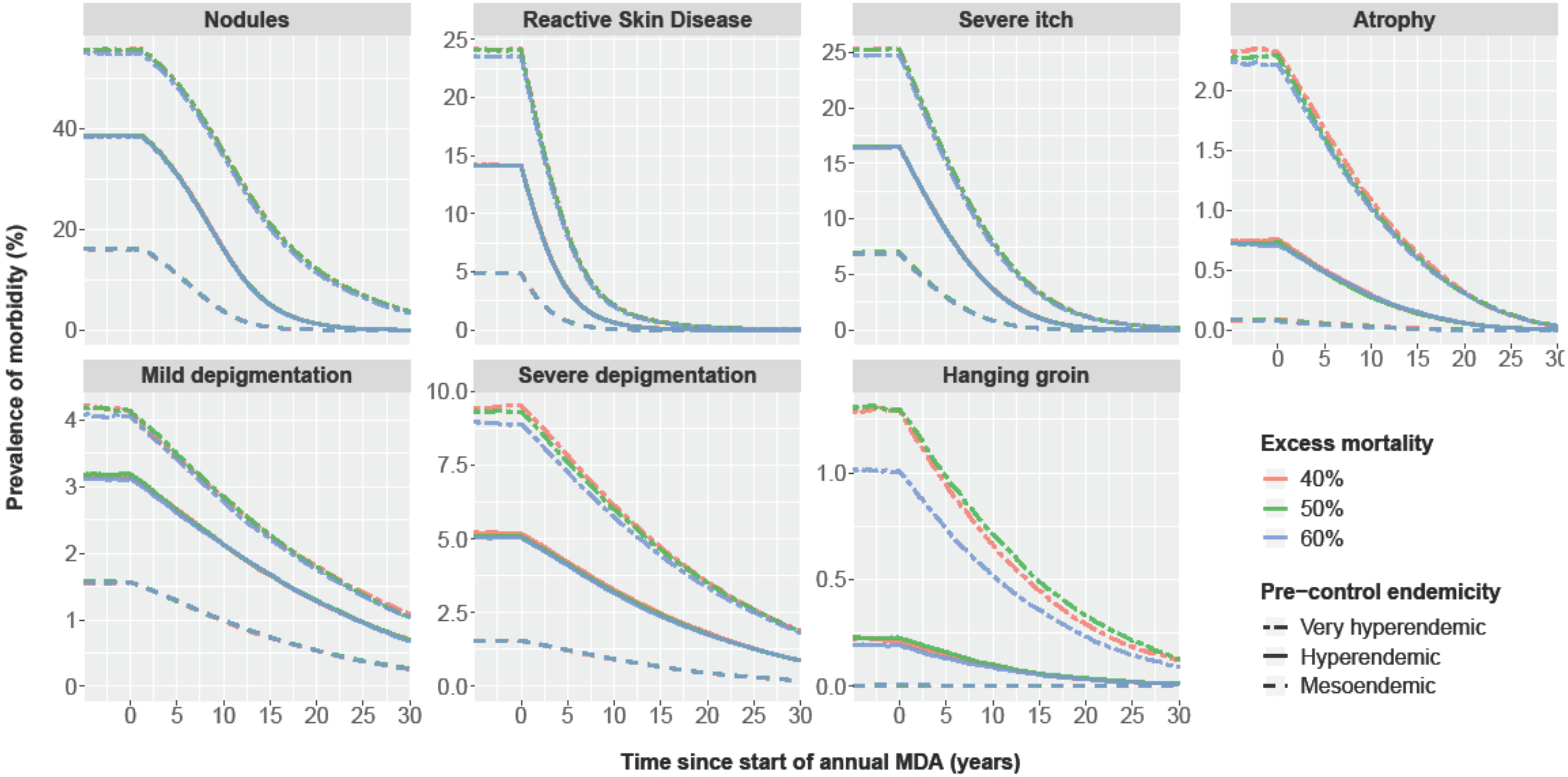

S29 Fig. Predicted impact of annual MDA (70% treatment coverage) on onchocercal eye disease (OED) with varying excess mortality due to onchocercal blindness.

Coloured lines represent different percentages of excess mortality at 70% population coverage of annual MDA, and different line types represent the various pre-control endemicity levels. Different panels represent the various subtypes of OED in forest and savanna areas. The predicted trends are based on the average of 750 simulations. Please note the different scales for the y-axes in the panels.

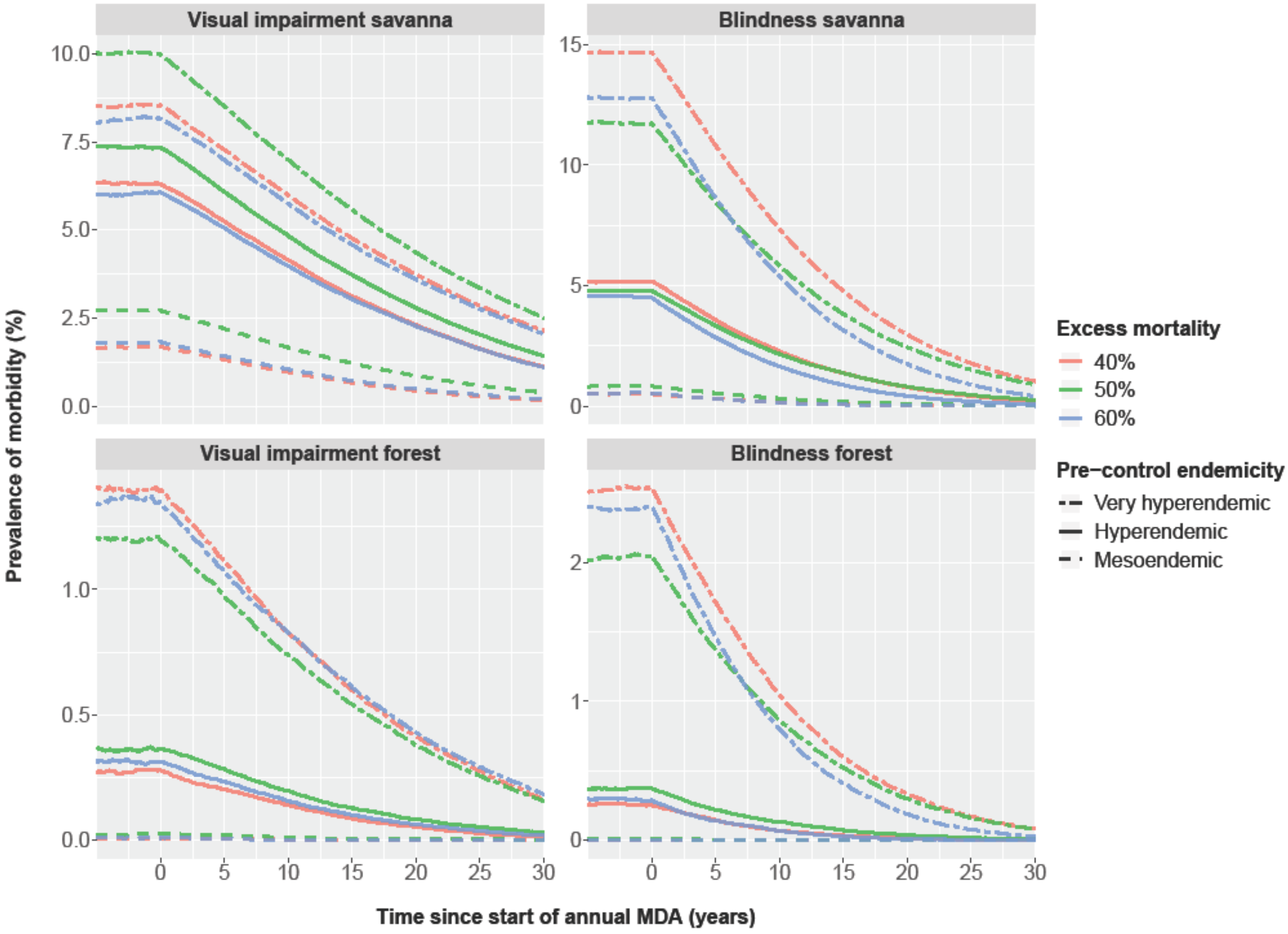

6. References

1. Plaisier A, van Oortmarssen G, Habbema J, Remme J, Alley E (1990) ONCHOSIM: a model and computer simulation program for the transmission and control of onchocerciasis. *Comput Methods Programs Biomed* **31**: 43–56.
2. Habbema J, van Oortmarssen G, Plaisier A (1996) The ONCHOSIM model and its use in decision support for river blindness control. *Cambridge University Press*: 360–380.
3. Alley WS, van Oortmarssen GJ, Boatin BA, Nagelkerke NJ, Plaisier AP, et al. (2001) Macrofilaricides and onchocerciasis control, mathematical modelling of the prospects for elimination. *BMC Public Health* **1**: 12.
4. Stolk WA, Walker M, Coffeng LE, Basáñez M-G, de Vlas SJ (2015) Required duration of mass ivermectin treatment for onchocerciasis elimination in Africa: a comparative modelling analysis. *Parasit Vectors* **8**(552).
5. Coffeng LE, Stolk WA, Hoerauf A, Habbema D, Bakker R, et al. (2014) Elimination of African onchocerciasis: modeling the impact of increasing the frequency of ivermectin mass treatment. *PLoS One* **9**: e115886.
6. Coffeng LE, Stolk WA, Zouré HGM, Veerman JL, Agblewonu KB, et al. (2013) African Programme for Onchocerciasis Control 1995-2015: model-estimated health impact and cost. *PLoS Negl Trop Dis* **7**: e2032.
7. Winnen M, Plaisier AP, Alley ES, Nagelkerke NJD, van Oortmarssen G, et al. (2002) Can ivermectin mass treatments eliminate onchocerciasis in Africa? *Bull World Health Organization* **80**: 384–391.
8. Coffeng LE, Bakker R, Montresor A, de Vlas SJ (2015) Feasibility of controlling hookworm infection through preventive chemotherapy: a simulation study using the individual-based WORMSIM modelling framework. *Parasit Vectors* **8**: 541.
9. Lont YL, Coffeng LE, de Vlas SJ, Goldon A, de los Santos T, et al. (2017) Modelling anti-Ov16 IgG4 antibody prevalence as an indicator for evaluation and decision making in onchocerciasis elimination programmes. *PLoS Negl Trop Dis.* **11**(1): e0005314
10. Duke BO (1993) The population dynamics of *Onchocerca volvulus* in the human host. *Trop Med Parasitol* **44**: 61–68.
11. Filipe JAN, Boussinesq M, Renz A, Collins RC, Vivas-Martinez S, et al. (2005) Human infection patterns and heterogeneous exposure in river blindness. *Proc Natl Acad Sci USA* **102**: 15265–15270.

12. Basanez M-G, Boussinesq M (1999) Population biology of human onchocerciasis. *Philos Trans R Soc B Biol Sci* **354**: 809–826.
13. Remme J, Ba O, Dadzie KY, Karam M (1986) A force-of-infection model for onchocerciasis and its applications in the epidemiological evaluation of the Onchocerciasis Control Programme in the Volta River basin area. *Bull World Health Organization* **64**: 667–681.
14. Remme JHF (2004) The Global Burden of Onchocerciasis in 1990. *World Health Organization*.
15. Plaisier AP, van Oortmarssen GJ, Remme J, Habbema JD (1991) The reproductive lifespan of *Onchocerca volvulus* in West African savanna. *Acta Trop* **48**: 271–284.
16. Duke BO (1980) Observations on *Onchocerca volvulus* in experimentally infected chimpanzees. *Tropenmed Parasitol* **31**: 41–54.
17. Prost A (1980) Latency period in onchocerciasis. *Bull World Health Organization* **58**: 923–925.
18. Kwarteng A, Ahuno ST, Akoto FO (2016) Killing filarial nematode parasites: role of treatment options and host immune response. *Infect Dis poverty* **5**: 86.
19. Plaisier AP, Alley ES, Boatin BA, Van Oortmarssen GJ, Remme H, et al. (1995) Irreversible effects of ivermectin on adult parasites in onchocerciasis patients in the Onchocerciasis Control Programme in West Africa. *J Infect Dis* **172**: 204–210.
20. Murdoch ME, Asuzu MC, Hagan M, Makunde WH, Ngoumou P, et al. (2002) Onchocerciasis: the clinical and epidemiological burden of skin disease in Africa. *Ann Trop Med Parasitol* **96**: 283–296.
21. Coffeng LE, Pion SDS, O'Hanlon S, Cousens S, Abiose AO, et al. (2013) Onchocerciasis: the pre-control association between prevalence of palpable nodules and skin microfilariae. *PLoS Negl Trop Dis* **7**: e2168.
22. Brieger WR, Awedoba AK, Eneanya CI, Hagan M, Ogbuagu KF, et al. (1998) The effects of ivermectin on onchocercal skin disease and severe itching: results of a multicentre trial. *Trop Med Int Health* **3**: 951–961.
23. Ozoh GA, Murdoch ME, Bissek A-C, Hagan M, Ogbuagu K, et al. (2011) The African Programme for Onchocerciasis Control: impact on onchocercal skin disease. *Trop Med Int Health* **16**: 875–883.
24. United Nations Department of Economic and Social Affairs Population Division. Accessed on: 19 Dec 2019. Available at: https://www.un.org/en/development/desa/publications/world-population-prospects-the-2012-revision.html. (2013) World Population Prospects: the 2012 revision, Volume I: Comprehensive Tables.

25. Alley ES, Plaisier AP, Boatin BA, Dadzie KY, Remme J, et al. (1994) The impact of five years of annual ivermectin treatment on skin microfilarial loads in the onchocerciasis focus of Asubende, Ghana. *Trans R Soc Trop Med Hyg* **88**: 581–584.
26. Plaisier A (1996) Modelling onchocerciasis transmission and control [PhD Thesis]. Accessed on: 17 Dec 2019. Available at: https://repub.eur.nl/pub/21404. *Erasmus University Rotterdam*.
27. Albiez EJ (1985) Calcification in adult *Onchocerca volvulus*. *Trop Med Parasitol* **36**: 180–181.
28. Karam M, Schulz-Key H, Remme J (1987) Population dynamics of *Onchocerca volvulus* after 7 to 8 years of vector control in West Africa. *Acta Trop* **44**: 445–457.
29. Schulz-Key H, Karam M (1986) Periodic reproduction of *Onchocerca volvulus*. *Parasitol Today* **2**: 284–286.
30. Schulz-Key H (1990) Observations on the reproductive biology of *Onchocerca volvulus*. *Acta Leiden* **59**: 27–44.
31. Prost A, Vaugelade J (1981) Excess mortality among blind persons in the West African savannah zone. *Bull World Health Organization* **59**: 773–776.
32. Dadzie KY, Remme J, Rolland A, Thylefors B (1986) The effect of 7-8 years of vector control on the evolution of ocular onchocerciasis in West African savanna. *Trop Med Parasitol* **37**: 263–270.
33. Plaisier AP, van Oortmarssen, Gerrit J. Remme JHF, Alley ES, Habbema JDF (1991) The risk and dynamics of onchocerciasis recrudescence after cessation of vector control. *Bull World Health Organization* **69**: 169–178.
34. Phillipon B (1977) Étude de la transmission d'*Onchocerca volvulus* (Leuckart, 1893) (Nematoda, Onchocercidae) par *Simulium damnosum* Theobald, 1903 (Diptera, Simuliidae) en Afrique tropicale. *ORSTOM: Paris*.
35. World Health Organization (1989) Onchocerciasis Control Programme in West Africa: report of the annual OCP research meeting, 20-24 March 1989.
36. Murdoch ME, Murdoch IE, Evans J, Yahaya H, Njepuome N, et al. (2017) Pre-control relationship of onchocercal skin disease with onchocercal infection in Guinea Savanna, Northern Nigeria. *PLoS Negl Trop Dis* **11**: e0005489.
37. Coffeng LE, Fobi G, Ozoh G, Bissek AC, Nlatté BO, et al. (2012) Concurrence of dermatological and ophthalmological morbidity in onchocerciasis. *Trans R Soc Trop Med Hyg* **106**: 243–251.
38. Alonso L, Murdoch M, Jofre-Bonet M (2009) Psycho-social and economical evaluation of

onchocerciasis: a literature review. *Soc Med* **4**: 8–31.

39. Prost A, Hervouet J, Thylefors B (1979) The degrees of endemicity of onchocerciasis. *Bull World Health Organization* **57**: 655–662.

40. Remme J, Ba O, Dadzie KY, Karam M (1986) A force-of-infection model for onchocerciasis and its applications in the epidemiological evaluation of the Onchocerciasis Control Programme in the Volta River basin area. *Bull World Health Organization* **64**: 667–681.

41. Bird AC, Anderson J, Fuglsang H (1976) Morphology of posterior segment lesions of the eye in patients with onchocerciasis. *Br J Ophthalmol* **60**: 2–20.

42. Anderson J, Fuglsang H (1977) Ocular onchocerciasis. *Trop Dis Bull* **74**: 257–272.
