# Supplementary material for: How does onchocerciasis-related skin and eye disease in Africa depend on cumulative exposure to infection and mass treatment?
Source: PLoS Negl Trop Dis. 2021 Jun 11;15(6):e0009489. doi: 10.1371/journal.pntd.0009489 (PMC8221783; doi:10.1371/journal.pntd.0009489)
Supplement: Attachment — Submitted filename: Reaction to the reviewers_2021.04.28.docx [file pntd.0009489.s002.docx]

**Reaction to the reviewers**

**Manuscript: PNTD-D-20-01988**

**How does onchocerciasis-related skin and eye disease in Africa depend on cumulative exposure to infection and mass treatment**

**Natalie V.S. Vinkeles Melchers, Wilma A. Stolk, Michele E. Murdoch, Belén Pedrique, Marielle Kloek, Roel Bakker, Sake J. de Vlas, Luc E. Coffeng**

We would like to thank all reviewers for their comments and suggestions for improvement. We numbered the comments of the reviewers consecutively for ease of reference. The line numbers in our answers correspond to the PDF version of the manuscript with track changes.

**Reviewer's Responses to Questions**

**Methods**

**Reviewer #1**

While the methods are clearly stated and easy to follow, I do not have sufficient transmission modelling experience to comment on whether the models used are appropriate.

**Reviewer #2**

The paper submitted by Vinkeles Melchers et al. describes how new modules were developed within the general Onchosim model to represent the progression of clinical manifestations of onchocerciasis, and their regression after ivermectin treatment. Once the parameters defined, the authors applied the model to assess the long-term impact of repeated ivermectin treatments on the prevalence of the main cutaneous and ocular signs of the disease.
The work to reach these objectives is very impressive and all the methods used are very well described in the paper and in the detailed supplementary material. This is a major strength of this paper.

1. **However, the authors certainly did not use all the published or potentially available data to “feed” the model and refine the various parameters. The parameters used to describe the relationships between the pre-treatment prevalence of infection and the prevalence of the various manifestations were evaluated using data from several thousands of people but those used for the cutaneous signs come from only four articles. One understands that the authors wished to include only papers where the cutaneous signs are defined according to Murdoch et al. (1993). Notwithstanding, they could have performed sensitivity analyses using data published in other articles which are not cited in this manuscript: for example, Edungbola et al., 1987, Carme et al., 1993, or Kollo et al., 1995, for depigmentation. What is the best approach to develop a model? relatively few precise data, or more less precise data? This has to be discussed.**

We have added a paragraph in the Discussion to discuss the reviewer’s comment: “As there is significant variability in the characterisation of cutaneous signs, such as of depigmentation, as well as potential variability between study designs or screening approaches, we have only used data where the cutaneous signs are defined according to Murdoch et al (Murdoch et al., 1993), for both the model quantification as well as the external validation. The variability in methodological approaches as well as differences in bioclimatic and epidemiological settings, may lead to different associations between infection and disease. For example, in communities of southern Cameroon, a higher prevalence of any depigmentation was reported for a given nodule prevalence as compared to our association, i.e. for a nodule prevalence of 40%, they reported a ~30% prevalence of any depigmentation, and at 80% nodule prevalence a ~60% depigmentation prevalence (Kollo, Mather, & Cline, 1995). On the other hand, the associations between the prevalence of infection and depigmentation in rural villages in Kwara State in Nigeria (Edungbola et al., 1987) and the Republic of Congo (Carme, Ntsoumou-Madzou, Samba, & Yebakima, 1994) are of the same order of magnitude as our predictions.” [lines 535-546]

2. **Regarding the effects of ivermectin on cutaneous signs of onchocerciasis, the authors used data from about 350 subjects examined before and one year after a single dose of ivermectin (Brieger et al.) and about 5200 subjects examined before and 5-6 years after implementation of annual treatments (as part of a study funded by APOC). Even if the data on the effects of ivermectin on onchodermatitis is scarce, it is not the case for nodule prevalences, whose change over time has been published in a number of papers. Again, the authors should explain why they excluded these datasets.**

There are indeed several papers that assessed the impact of ivermectin MDA on the prevalence of palpable nodules and some other onchocercal skin diseases. We did not perform a (systematic) review to include all available longitudinal data of the impact of ivermectin on onchocercal skin disease as we wanted to avoid heterogeneity between studies in clinical screening and study designs. Therefore, we used the longitudinal studies by Brieger et al 1998 and Ozoh et al 2011. The paper by Ozoh et al. was performed by a subset of the same study team as the multi-county dataset used in this study (Murdoch et al 2002). The Brieger et al. study is one of the rare studies describing longitudinal impact of ivermectin on itch while distinguishing severe itch (i.e. attempting to make it more specific for onchocerciasis). We now explain this in the Discussion and devote some attention to the longitudinal results of two additional papers:

“For the quantification of the reversible clinical skin manifestations, we also used longitudinal trends. For longitudinal impact of ivermectin on itch, we used the Brieger et al. (Brieger et al., 1998) study, one of the few studies on the impact of ivermectin on itch that distinguishes severe itch (which is more specific for onchocerciasis). For the remaining morbidity patterns, we included longitudinal data collected by the same investigators using the same screening methods to limit heterogeneity between studies (Ozoh et al., 2011). In addition, our model predictions agree well with reported impact of eight to ten years of annual MDA on palpable nodules in selected hyperendemic villages in Nigeria, Cameroon and Uganda (Emukah et al., 2004; Katabarwa et al., 2008).” [lines 496-503].

**Reviewer #3**

3. **The objectives of the study are clearly articulated, and the study design is appropriate to address the stated objectives. The overall methodology seems sound although additional details of the mathematical formulations used in calculating tissue damage would be appreciated, for fully assessing the approach. I don't have any concerns about ethical or regulatory requirements.**

Tissue damage is simualted by applying the equation for change in total damage (${\Delta D}_{\mathrm{ix}}(t))$in Table S1 at every time step in the simulation. The equation captures how tissue accumulates ($S_{\mathrm{ix}}\times d_{i}\left( t \right)$) and disappears/heals ($-r_{x}\times D_{\mathrm{ix}}(t)$). There are no further equations or steps. We have added the following: “(see Supplementary Table S1 for mathematical equations and explanation of how tissue damage simulated)” [lines 137-138] for readers to be able to find the formulations easily.

4. **Other** **specific comments on the Methods section:
   Line 147:** **Why are mf killed through treatment considered to not cause tissue damage? Is their contribution to morbidity considered too transitory? This should be justified and referenced.**

We have added in the text: “There are some reports of adverse effects (generic symptoms, e.g. oedema, fever, pain) upon ivermectin treatment within 24-48 hours after intake, but these reactions were generally mild and self-limiting (Pacqué, Munoz, Greene, & Taylor, 1991; Tielsch & Beeche, 2004; Zea-Flores et al., 1992). There is evidence that *O. volvulus* mf in patients treated with ivermectin first migrate to regional lymph nodes where they degenerate and are encircled by eosinophils or macrophages. As a result, inflammatory cellular reactions due to the death of mf in the tissues upon ivermectin intake is minimal (B. O. L. Duke, Soula, Zea-Flores, Bratthauer, & Doumbo, 1991) (in contrast to diethylcarbamazine which gives a strong histological reaction within ocular tissue accelerating onchocercal blindness (Taylor et al., 1986)). Treatment is further assumed to only indirectly affect the development and presence of symptoms via removal of mf which would have caused damage if they would have died naturally.” [lines 154-162]

5. **Lines 154–160, 183-184: Similarly, these modelling decisions, while reasonable, should be justified either on the grounds that they provide flexibility in the model (i.e., require fewer constraining assumptions) or documentation of such variability from the literature.**

We added in the text: “There is evidence that there are various determinants that lead to variation in susceptibility to infectious disease susceptibility, including host and pathogen genetic variation and immune effectiveness (Baker & Antonovics, 2012; Canales & Walz, 2011).“ [lines 169-171]

6. **Line 133: Please explain here how tissue damage is quantified, i.e., is it a unitless quantity whose meaning is only relevant within this model, or does it have a broader interpretation?**

Tissue damage is proportional to the actual number of mf that ever died in a person, minus recovery from tissue damage. It has no broader meaning or interpretation.

7. **Line 293: Is this value of 0.5% prevalence consistent with programmatic goals as stated by WHO or individual country programs? If so, please cite. If not, please justify this choice.**

We have added to the methods section: “To assess how long it takes for the various disease outcomes to largely disappear from a population, we estimate after how many years of MDA the prevalence of clinical manifestations falls below an arbitrary threshold of 0.5%. We note that this may be longer than the duration of MDA required for interruption of transmission, as the latter does not require infection to be completely cleared from a population (Coffeng et al., 2019, 2014) and because chronic symptoms like blindness persist after clearing infection (WHO, 2001).” [lines 313-318]

**Results**

**Reviewer #1**

No Response

**Reviewer #2**

The analysis presented matches the analysis plan. The results are clearly and completely presented. The figures (Tables, Images) are of sufficient quality for clarity.

**Reviewer #3**

8. **The outline of the results matches the methods and the results are clearly and completely presented. The figures are useful and well-done, with minor suggestions below. My overarching suggestions for improvements are (1) to provide quantifications of in-sample and out-of-sample prediction error (currently only qualitative assessments of validation are presented).**

We have now added in-sample prediction errors by reporting the root mean squared error for each model fit (see Supplement S1, Table S3). We indeed did not perform any formal quantification of out-of-sample prediction errors, as there is no recent data available on morbidity reductions. To compensate for this, we performed a wide range of sensitivity analysis to examine which assumptions and model parameters have the most impact on predicted trends in morbidity.

9. **and (2) to incorporate measures of model uncertainty into the reporting of results, including figures (uncertainty is mentioned on lines 422–423 but otherwise is not addressed). These considerations are crucial to evaluating the robustness of the model estimates.**

We assess the robustness of our model by examining the extent to which our results are affected by changes in biological and programmatic assumptions. We have thus assessed uncertainty around our predictions rather than assessing uncertainty of the data we used. Section 5 of Supplement S1 describes the various sensitivity analyses performed and their results. We have shown that the model is robust for assumptions concerning programmatic variables, but that there are considerable biological uncertainties around the regression of onchocercal eye disease and excess mortality due to blindness. We have discussed the possible explanations and limitations in Supplement S1 (section 5.2) as well as in the Discussion of the main manuscript [lines 533-581].

10. **Other comments regarding the results:
    Line 326: I believe that the threshold values for savanna areas is reversed and should read “…(1.7 vs. 3.1 thousand, respectively)…” Also, please discuss the relevance of focusing on relative differences in disease thresholds versus absolute differences, as the absolute differences are actually larger in forest areas.**

Thank you for noting. The disease thresholds for savanna versus forest areas are correctly reported, but the sentence was not correctly phrased. This is now adapted: “The difference between the disease thresholds of visual impairment and blindness was higher in forest areas (10.5 versus 12.5 thousand, respectively) than in savanna areas (1.7 versus 3.1 thousand, respectively), which reflects that only a small additional amount of tissue damage for people with visual impairment in savanna areas is needed to become blind (Supplementary Table S3).” [lines 349-353] We also changed “relative difference” to just “difference” as the measures reported here are absolute differences.

11. **Figure 4: Please clarify what the three sets of estimates for each clinical manifestation represent (not the three MDA coverage levels indicated by color); presumably these reflect hypo-, meso- and hyperendemic starting conditions? This should be stated in the figure caption, at least.**

We have adapted this, see the current Figure 4.

**Conclusions**

**Reviewer #1**

No Response

**Reviewer #2**

12. **As mentioned above, the authors should clearly explain why they used data from a relatively short list of studies, which could be regarded as a limitation of the paper, and why others were excluded, even for sensitivity analyses.**

Please see response #1 of this document.

13. **Regarding the data on the cutaneous manifestations, figure 3 shows that their prevalence can vary widely for a given prevalence of infection. The authors hypothesize that this variability could be due to differences in pathogenicity between savanna and forest “strains”. They could cite the classical papers by Anderson et al. (TRSTMH 1974), where this difference was explored, to strengthen this hypothesis.**

We have added two more references, including the reference mentioned by the reviewer. We now refer to Anderson et al., 1974; Duke et al., 1966; Erttmann et al., 1987; Zimmerman et al., 1992 [line 554].

14. **The possibility that visual impairment could be reversible is considered in the Discussion section (from lines 512), and this is certainly a key point. More references on this possibility could be cited (for example Mabey et al., 1996; Chippaux et al., 1999; Banla et al., 2014 …).**

We have added these references. We now refer to Banla et al., 2014; Chippaux et al., 1999; Ejere et al., 2012; Mabey et al., 1996 [line 567].

**Reviewer #3**

15. **The conclusions are generally supported by the data presented. The discussion sufficiently addresses limitations of the analysis, although I would like to see some mention of the limitations of the chosen modelling approach to quantifying uncertainty in the underlying parameters and estimates (e.g., as compared with a Bayesian statistical model).**

We have added in the Discussion “Finally, we have used dynamic mechanistic processes of disease accumulation to quantify our model. Such a mechanistic mathematical model is more appropriate to forecast disease prevalence over time than statistical modelling. However, fitting such more sophisticated stochastic models with advanced techniques to quantify uncertainty (e.g. Bayesian frameworks) is technically highly challenging and computationally demanding, and we therefore opted for a simpler approach for model quantification and instead performed extensive sensitivity analyses.” [lines 588-594]

16. **The authors clearly discuss the public health relevance of this work. I would like to see some discussion of any existing targets for morbidity reduction, such as from WHO or individual country programs. This would be valuable in understanding the context of the 0.5% prevalence target considered negligible by the authors.**

There are no morbidity prevalence targets for onchocerciasis to our knowledge. WHO has not set any onchocerciasis-specific targets for 2030. The new roadmap contains a general target, to achieve a 75% reduction in DALYs related to NTDs by 2030. There are also some targets related to implementation of integrated skin NTD strategies, case management, and adequate support for people with chronic disease. We have also adapted the manuscript to avoid the qualitative statement “negligible” as this qualification depends on the symptom and this severity. See also response #7.

17. **Other comments:
    Lines 458–459: Please provide the results supporting this conclusion in a table in the main text.**

If the reviewer refers to “*In areas of very high pre-control onchocerciasis endemicity, the relative reduction in the prevalence of chronic morbidity ranged from 70% to 89% after 30 years of moderate annual MDA coverage (60%)*.” This is a summary statement based on the results described in the third to fifth paragraphs of “*Model-predicted impact of MDA on the prevalence of disease”* in the results section*.* We have added a summary table to the main text (Table 2).

18. **Line 507: Maybe replace “…related to the biology of different parasite species” with something like  “…related to genetic variation in O. volvulus.”**

Adapted, see lines 553.

19. **Line 545: Please define DALYs.**

Adapted, see line 604-606: “This disease module will be used to estimate trends in the onchocercal disease burden in terms of Disability Adjusted Life Years lost due to onchocerciasis in Africa with stratifications by age, sex, and country”.

**Editorial and Data Presentation Modifications**

**Reviewer #1**

No Response

**Reviewer #2**

20. **S1, page 14, below the formula: replace one “y_obs_frac” by “y_hat_frac”**

Thank you for noticing this relevant detail. This was adapted.

21. **Some references are incomplete (16, 64)**

Adapted. The remaining reference list was also cross-checked.

22. **The acknowledgments are restricted to the investigators of the Murdoch et al studies, and could be extended to the other investigators of the key papers used to develop the model, and to the organizations that funded these studies (TDR, APOC, …)**

We have only added the study collaborators of the Murdoch et al. multi-country dataset as we received the raw data from them. This was their specific requirement for sharing the data with us. All other data on the association between infection and morbidity used in this study was extracted from figures and tables in existing literature. We have however added that the multi-country study on OSD was financially supported by UNDP/World Bank/WHO Special Programme for Research and Training in Tropical Diseases (TDR). [lines 637-639]

**Reviewer #3**

23. **Comments on the Background: Line 88: The introduction is excellent. I suggest only adding a very brief description of past estimates of onchocerciasis burden to the end of the first paragraph (line 88) to provide context for the scale of infection and morbidity prevalence, e.g., referencing the following sources:**

We added the following short paragraph in the Background: “According to estimates, about 7.5 million people were infected with *O. volvulus* in West-Africa around 1974 (prior the implementation of the Onchocerciasis Control Programme [OCP]) (O’Hanlon et al., 2016). Another study estimated that 36 million people would have been infected in the APOC countries by 2011 if there had been no ivermectin treatment (Zouré et al., 2014).” [lines 88-91] We did not include the papers by Kelly-Hope et al (2018) as the papers concerns LF-loiasis. Neither did we include the paper by Sauerbrey et al (2018) as their work focuses on the Americas, which is outside the scope of our paper.

24. **Comments on the Supplementary Information:
    Page 32: It is claimed that Figure S5 “shows that the model predictions fit quite well with available external data,” but my impression from the figure is that the fit is often actually not very good except for “any DPM” (recognizing that the panel for any vs. severe itch is not comparing identical quantities). Can model fit be quantified and reported, with perhaps a more nuanced discussion of model prediction accuracy?**

We agree with the reviewer that the fit of model predictions to external data is not as good for all clinical manifestations as for “any depigmentation”. We have adapted the text in the Supplement to reflect this. In the Supplement S1, we adapted and added the following text: “Figure S5 shows that the fit of model predictions to external data is not as good for all clinical manifestations as for “any depigmentation”. The study sites from the multi-country dataset by Murdoch et al. (Murdoch et al., 2002) were all from forest and forest-savanna mixed bioclimates whereas the Kaduna (Nigeria) study site has a savanna bioclimate (Murdoch et al., 2017). The systematic differences in model predictions and the external data from Kaduna may arise from genetic variation in *O. volvulus* parasites.” (p32 of Supplement S1).

In the Discussion, we already describe the following: “Such geographical variation may occur by chance, although we cannot exclude the possibility of systematic differences in the prevalence of subtypes of OSD between savannah and forest areas, similar to variations measured in OED prevalence related to genetic variation in *O. volvulus* (Anderson et al., 1974; Duke et al., 1966; Erttmann et al., 1987; Zimmerman et al., 1992)*.“* We now have added: “For endemic areas previously under the APOC-mandate, the difference in the occurrence of skin disease between forest versus savanna areas is of less relevance as the majority of endemic areas are of the forest type. However, when implementing the model in other areas (e.g., countries formerly covered by OCP), one should be aware of potential differences in morbidity prevalence between the bioclimates.” [lines 554-558]

25. **Figures S11–S13: These are valuable figures, as they illustrate one important source of uncertainty in model estimates. However, the figures could be improved by either (1) reducing the opacity of each line, so that the central trends become more apparent, or (2) showing the median values with 95% UI displayed as a faint ribbon.**

The respective figures are adapted according to the suggestions made by the reviewer.

**Summary and General Comments**

**Reviewer #1**

No Response

**Reviewer #2**

26. **A weakness of this paper is probably that there is only one clinician, and no ophthalmologist, among the authors. Clinicians would have probably helped in the development of some parts of the model. However, the paper is very interesting because it shows for the first time how the prevalence of each manifestation of onchocerciasis will tend to decrease in the future. However, as the parameters were assessed using data from relatively low numbers of subjects, they will probably evolve if more data becomes available.**

We acknowledge that no ophthalmologist was involved in this work. However, an ophthalmologist (Dr. Grace Fobi, affiliated to APOC at the time) was involved in the development of an earlier, simpler version of ONCHOSIM that only included vision loss and itch (Coffeng et al 2013 PLOS NTDs). This simpler model is nested within the morbidity module presented in this paper, which needed to be more sophisticated to capture the development and dynamics of skin disease, for which we included one of the most experienced dermatologists in the field of onchocerciasis (Michele Murdoch). We have further added the following to the discussion:

“Our model has shown for the first time how the prevalence of various manifestations of onchocerciasis are likely to decrease with ongoing MDA with ivermectin. It is anticipated that with future input from a wider field of clinicians, including those with expertise in onchocerciasis-associated epilepsy, further refinements to the model may be developed.” [Lines 599-602]

**Reviewer #3**

This manuscript represents an important advance in the development of modeling tools to support public health programs for the reduction of onchocerciasis morbidity. I applaud the authors for an exceptionally well-written and thorough paper. The broad scope of the sensitivity analyses is especially appreciated. My suggestions for changes, as detailed in other portions of this review, are relatively minor and mainly serve to provide additional context for the analysis and to provide additional information with which to ascertain model predictive accuracy and uncertainty.

**References**

Anderson, J., Fuglsang, H., Hamilton, P. J. S., & dE C. Marshall, T. F. (1974). Studies on onchocerciasis in the United Cameroon Republic II. Comparison of onchocerciasis in rain-forest and Sudan-savanna. *Transactions of the Royal Society of Tropical Medicine and Hygiene*, *68*(3), 209–222. https://doi.org/10.1016/0035-9203(74)90117-5

Baker, C., & Antonovics, J. (2012). Evolutionary determinants of genetic variation in susceptibility to infectious diseases in humans. *PLoS ONE*, *7*(1), 29089. https://doi.org/10.1371/journal.pone.0029089

Banla, M., Tchalim, S., Karabou, P. K., Gantin, R. G., Agba, A. I., Kére-Banla, A., … Soboslay, P. T. (2014). Sustainable control of onchocerciasis: ocular pathology in onchocerciasis patients treated annually with ivermectin for 23 years: a cohort study. *PloS ONE*, *9*(6), e98411. https://doi.org/10.1371/journal.pone.0098411

Brieger, W. R., Awedoba, A. K., Eneanya, C. I., Hagan, M., Ogbuagu, K. F., Okello, D. O., … Remme, J. H. (1998). The effects of ivermectin on onchocercal skin disease and severe itching: results of a multicentre trial. *Trop Med Int Health*, *3*(12), 951–961. Retrieved from http://www.ncbi.nlm.nih.gov/pubmed/9892280

Canales, C. P., & Walz, K. (2011). Copy number variation and susceptibility to complex traits. *EMBO Molecular Medicine*, *3*(1), 1–4. https://doi.org/10.1002/emmm.201000111

Carme, B., Ntsoumou-Madzou, V., Samba, Y., & Yebakima, A. (1994). Prevalence of depigmentation of the shins: a simple and cheap way to screen for severe endemic onchocerciasis in Africa. *Bull World Health Organ*, *72*(2), 755–758.

Chippaux, J. P., Boussinesq, M., Fobi, G., Lafleur, C., Audugé, A., Banos, M. T., … Prod’hon, J. (1999). Effect of repeated ivermectin treatments on ocular onchocerciasis: Evaluation after six to eight dosings. *Ophthalmic Epidemiology*, *6*(4), 229–246. https://doi.org/10.1076/opep.6.4.229.4185

Coffeng, L. E., Stolk, W. A., Golden, A., de Los Santos, T., Domingo, G. J., & de Vlas, S. J. (2019). Predictive Value of Ov16 Antibody Prevalence in Different Sub-Populations for Elimination of African Onchocerciasis. *Am J Epidemiol*, *351*(9105), 843. https://doi.org/10.1093/aje/kwz109

Coffeng, L. E., Stolk, W. A., Hoerauf, A., Habbema, D., Bakker, R., Hopkins, A. D., & de Vlas, S. J. (2014). Elimination of African onchocerciasis: modeling the impact of increasing the frequency of ivermectin mass treatment. *PloS ONE*, *9*(12), e115886. https://doi.org/10.1371/journal.pone.0115886

Duke, B. O. L., Soula, G., Zea-Flores, G., Bratthauer, G. L., & Doumbo, O. (1991). Migration and death of skin-dwelling Onchocerca volvulus microfilariae after treatment with ivermectin. *Tropical Medicine and Parasitology*, *42*(1), 25–30.

Duke, B. O., Lewis, D. J., & Moore, P. J. (1966). Onchocerca-Simulium complexes. I. Transmission of forest and Sudan-savanna strains of Onchocerca volvulus, from Cameroon, by Simulium damnosum from various West African bioclimatic zones. *Annals of Tropical Medicine and Parasitology*, *60*(3), 318–326.

Edungbola, L., Alabi, T., Oni, G., Asaolu, S., Ogunbanjo, B., & Parakoyi, B. (1987). “Leopard Skin” as a rapid diagnostic index for estimating the endemicity of African onchocerciasis. *International Journal of Epidemiology*, *16*(4), 590–594.

Ejere, H. O., Schwartz, E., Wormald, R., & Evans, J. R. (2001). Ivermectin for onchocercal eye disease (river blindness). *Cochrane Database Syst Rev*, (8), CD002219. https://doi.org/10.1002/14651858.CD002219.pub2

Emukah, E. C., Osuoha, E., Miri, E. S., Onyenama, J., Amazigo, U., Obijuru, C., … Richards, F. O. (2004). A longitudinal study of impact of repeated mass ivermectin treatment on clinical manifestations of onchocerciasis in Imo State, Nigeria. *American Journal of Tropical Medicine and Hygiene*, *70*(5), 556–561. https://doi.org/10.4269/ajtmh.2004.70.556

Erttmann, K. D., Unnasch, T. R., Greene, B. M., Albiez, E. J., Boateng, J., Denke, A. M., … Williams, P. N. (1987). A DNA sequence specific for forest form Onchocerca volvulus. *Nature*, *327*(6121), 415–417. https://doi.org/10.1038/327415a0

Katabarwa, M., Eyamba, A., Habomugisha, P., Lakwo, T., Ekobo, S., Kamgno, J., … Richards, F. O. (2008). After a decade of annual dose mass ivermectin treatment in Cameroon and Uganda, onchocerciasis transmission continues. *Tropical Medicine and International Health*, *13*(9), 1196–1203. https://doi.org/10.1111/j.1365-3156.2008.02126.x

Kollo, B., Mather, F. J., & Cline, B. L. (1995). Evaluation of alternate methods of rapid assessment of endemicity of Onchocerca volvulus in communities in southern Cameroon. *American Journal of Tropical Medicine and Hygiene*, *53*(3), 243–247. https://doi.org/10.4269/ajtmh.1995.53.243

Mabey, D., Eckstein, M., Gilbert, C., & Whitworth, J. (1996). Ocular onchocerciasis - the effects of six years of treatment with ivermectin. *IOVS*, *37*, ARVO Abstract 1672.

Murdoch, M. E., Hay, R. J., Mackenzie, C. D., Williams, J. F., Ghalib, H. W., Cousens, S., … Jones, B. R. (1993). A clinical classification and grading system of the cutaneous changes in onchocerciasis. *Br J Derm*, *129*(3), 260–269. Retrieved from http://www.ncbi.nlm.nih.gov/pubmed/8286222

O’Hanlon, S. J., Slater, H. C., Cheke, R. A., Boatin, B. A., Coffeng, L. E., Pion, S. D. S., … Basáñez, M.-G. (2016). Model-Based Geostatistical Mapping of the Prevalence of Onchocerca volvulus in West Africa. *PLoS Med*, *10*(1), e0004328. https://doi.org/10.1371/journal.pntd.0004328

Ozoh, G. A., Murdoch, M. E., Bissek, A.-C., Hagan, M., Ogbuagu, K., Shamad, M., … Amazigo, U. V. (2011). The African Programme for Onchocerciasis Control: impact on onchocercal skin disease. *Trop Med Int Health*, *16*(7), 875–883. https://doi.org/10.1111/j.1365-3156.2011.02783.x

Pacqué, M., Munoz, B., Greene, B. M., & Taylor, H. R. (1991). Community-based treatment of onchocerciasis with ivermectin: Safety, efficacy, and acceptability of yearly treatment. *Journal of Infectious Diseases*, *163*(2), 381–385. https://doi.org/10.1093/infdis/163.2.381

Taylor, H. R., Murphy, R. P., Newland, H. S., White, A. T., D’Anna, S. A., Keyvan-Larijani, E., … Greene, B. M. (1986). Treatment of onchocerciasis. The ocular effects of ivermectin and diethylcarbamazine. *Archives of Ophthalmology*, *104*(6), 863–870. https://doi.org/10.1001/archopht.1986.01050180097039

Tielsch, J. M., & Beeche, A. (2004). Impact of ivermectin on illness and disability associated with onchocerciasis. *Trop Med Int Health*, *9*(4), A45-56. https://doi.org/10.1111/j.1365-3156.2004.01213.x

WHO. (2001). Certification of elimination of human onchocerciasis: criteria and procedures. World Health Organization. WHO/CDS/CPE/CEE/2001.18a. Accessed on: 17.02.2021. Available at: http://www.oepa.net/Documentos/CriteriosCertificacionOMS/WHO_CDS_CPE_CEE_2001.18b.pd.

Zea-Flores, R., Richards, F. O., González-Peralta, C., Ramirez, J. C., Zea-Flores, G., Collins, R. C., & Cupp, E. (1992). Adverse reactions after community treatment of onchocerciasis with ivermectin in guatemala. *Transactions of the Royal Society of Tropical Medicine and Hygiene*, *86*(6), 663–666. https://doi.org/10.1016/0035-9203(92)90182-C

Zimmerman, P. A., Dadzie, K. Y., De Sole, G., Remme, J., Alley, E. S., & Unnasch, T. R. (1992). Onchocerca volvulus DNA probe classification correlates with epidemiologic patterns of blindness. *The Journal of Infectious Diseases*, *165*(5), 964–968.

Zouré, H. H. G. M., Noma, M., Tekle, A. H., Amazigo, U. V., Diggle, P. J., Giorgi, E., … Remme, J. H. F. (2014). The geographic distribution of onchocerciasis in the 20 participating countries of the African Programme for Onchocerciasis Control: (2) pre-control endemicity levels and estimated number infected. *Parasit Vectors*, *7*(326), 326. https://doi.org/10.1186/1756-3305-7-326
